# Supplementary material for: A green garlic (Allium sativum L.) based intercropping system reduces the strain of continuous monocropping in cucumber (Cucumis sativus L.) by adjusting the micro-ecological environment of soil
Source: PeerJ. 2019 Jul 15;7:e7267. doi: 10.7717/peerj.7267 (PMC6637937; doi:10.7717/peerj.7267)
Supplement: Data S1 [file peerj-07-7267-s001.zip › supplemental_Data_S1/15 days after interplanted/CB-1.rtf]

Volume: DATA            File: E131084.29A        Samp Ctr: 23                ID Number: 1002 
Type: Samp                   Bottle: 3                        Method: TSBA6 
Created: 1/8/2013 7:10:21 PM 
Sample ID: 57 


RT	Response	Ar/Ht	RFact	ECL	Peak Name	Percent	Comment1	Comment2	
1.645	4.515E+8	0.029	----	7.002	SOLVENT PEAK	----	< min rt		
1.777	2634	0.023	----	7.261		----	< min rt		
2.285	170	0.020	----	8.258		----	< min rt		
3.349	232	0.021	----	10.254		----			
4.907	1538	0.038	1.021	12.098	11:0 iso 3OH	0.60	ECL deviates  0.009		
5.112	2980	0.036	----	12.275		----			
5.504	296	0.029	1.002	12.612	13:0 iso	0.11	ECL deviates -0.002	Reference -0.006	
6.807	1527	0.038	0.975	13.620	14:0 iso	0.57	ECL deviates  0.001	Reference -0.002	
7.329	1700	0.035	0.967	14.000	14:0	0.63	ECL deviates  0.000	Reference -0.003	
7.779	7628	0.049	----	14.291		----			
8.007	653	0.034	0.960	14.439	15:1 iso G	0.24	ECL deviates -0.001		
8.293	14331	0.039	0.958	14.623	15:0 iso	5.22	ECL deviates  0.000	Reference -0.002	
8.433	8057	0.040	0.957	14.714	15:0 anteiso	2.93	ECL deviates  0.001	Reference -0.001	
8.877	1663	0.038	0.953	15.001	15:0	----	ECL deviates  0.001		
8.966	747	0.042	----	15.055		----			
9.619	1975	0.073	0.949	15.445	16:1 iso G	0.71	ECL deviates  0.003		
9.922	7004	0.040	0.948	15.627	16:0 iso	2.53	ECL deviates  0.000	Reference -0.002	
10.158	2067	0.047	0.947	15.768	16:1 w9c	0.74	ECL deviates -0.006		
10.240	21177	0.043	0.947	15.817	Sum In Feature 3	7.63	ECL deviates -0.005	16:1 w7c/16:1 w6c	
10.390	6489	0.040	0.947	15.907	16:1 w5c	2.34	ECL deviates -0.002		
10.544	35481	0.042	0.946	15.999	16:0	12.77	ECL deviates -0.001	Reference -0.003	
11.082	97554	0.058	----	16.310		----			
11.289	42276	0.079	0.945	16.430	Sum In Feature 9	15.20	ECL deviates -0.002	16:0 10-methyl	
11.429	8864	0.075	0.945	16.511	15:0 3OH	3.19	ECL deviates  0.008		
11.635	11065	0.058	0.944	16.630	17:0 iso	3.98	ECL deviates  0.000	Reference -0.003	
11.796	9308	0.057	0.944	16.723	17:0 anteiso	3.34	ECL deviates  0.000	Reference -0.003	
11.921	3272	0.057	0.944	16.795	17:1 w8c	1.18	ECL deviates  0.003		
12.085	7182	0.052	0.944	16.889	17:0 cyclo	2.58	ECL deviates  0.001		
12.276	1813	0.047	0.944	17.000	17:0	0.65	ECL deviates  0.000	Reference -0.003	
12.345	3055	0.044	0.944	17.039	16:1 2OH	1.10	ECL deviates -0.009		
12.989	1440	0.040	0.944	17.405	17:0 10-methyl	0.52	ECL deviates -0.004		
13.146	714	0.034	----	17.494		----			
13.547	4957	0.047	0.945	17.722	Sum In Feature 5	1.78	ECL deviates  0.002	18:2 w6,9c/18:0 ante	
13.635	20444	0.057	0.945	17.772	18:1 w9c	7.35	ECL deviates  0.003		
13.726	19264	0.048	0.945	17.823	Sum In Feature 8	6.92	ECL deviates  0.000	18:1 w7c	
13.873	2727	0.058	0.945	17.907	18:1 w5c	0.98	ECL deviates -0.012		
14.035	7471	0.046	0.945	17.999	18:0	2.69	ECL deviates -0.001	Reference -0.005	
14.179	1491	0.038	0.945	18.081	18:1 w7c 11-methyl	0.54	ECL deviates  0.000		
14.603	20331	0.066	----	18.324		----			
14.723	9890	0.058	0.946	18.393	18:0 10-methyl, TBSA	3.56	ECL deviates  0.001		
14.783	4924	0.048	----	18.427		----			
15.341	1113	0.048	0.946	18.747	Sum In Feature 6	0.40	ECL deviates -0.009	19:1 w11c/19:1 w9c	
15.619	15533	0.052	0.947	18.906	19:0 cyclo w8c	5.59	ECL deviates  0.004		
15.885	278306	0.163	----	19.059		----	> max ar/ht		
16.478	1478	0.043	0.947	19.402	20:4 w6,9,12,15c	0.53	ECL deviates  0.007		
17.121	1520	0.050	0.948	19.774	20:1 w9c	0.55	ECL deviates  0.004		
17.513	999	0.046	0.948	20.001	20:0	0.36	ECL deviates  0.001	Reference -0.008	
17.845	939	0.044	----	20.193		----	> max rt		
----	21177	---	----	----	Summed Feature 3	7.63	16:1 w7c/16:1 w6c	16:1 w6c/16:1 w7c	
----	4957	---	----	----	Summed Feature 5	1.78	18:2 w6,9c/18:0 ante	18:0 ante/18:2 w6,9c	
----	1113	---	----	----	Summed Feature 6	0.40	19:1 w11c/19:1 w9c	19:1 w9c/19:1 w11c	
----	19264	---	----	----	Summed Feature 8	6.92	18:1 w7c	18:1 w6c	
----	42276	---	----	----	Summed Feature 9	15.20	17:1 iso w9c	16:0 10-methyl	

ECL Deviation: 0.004                            Reference ECL Shift: 0.004      Number Reference Peaks: 12
Total Response: 690874                         Total Named: 277459
Percent Named: 40.16%                         Total Amount: 264413
Profile Comment:   Percent named is less than 85.00.

*** Library match not attempted
