# Supplementary material for: A green garlic (Allium sativum L.) based intercropping system reduces the strain of continuous monocropping in cucumber (Cucumis sativus L.) by adjusting the micro-ecological environment of soil
Source: PeerJ. 2019 Jul 15;7:e7267. doi: 10.7717/peerj.7267 (PMC6637937; doi:10.7717/peerj.7267)
Supplement: Data S1 [file peerj-07-7267-s001.zip › supplemental_Data_S1/15 days after interplanted/CB-2.rtf]

Volume: DATA            File: E131084.29A        Samp Ctr: 24                ID Number: 1003 
Type: Samp                   Bottle: 4                        Method: TSBA6 
Created: 1/8/2013 7:34:29 PM 
Sample ID: 58 


RT	Response	Ar/Ht	RFact	ECL	Peak Name	Percent	Comment1	Comment2	
1.645	4.542E+8	0.029	----	7.008	SOLVENT PEAK	----	< min rt		
1.778	3248	0.024	----	7.267		----	< min rt		
3.353	372	0.026	----	10.262		----			
4.776	267	0.032	----	11.978		----			
4.907	1149	0.033	1.021	12.094	11:0 iso 3OH	0.46	ECL deviates  0.005		
5.113	2477	0.035	----	12.273		----			
6.806	1394	0.036	0.975	13.619	14:0 iso	0.53	ECL deviates  0.000	Reference -0.002	
7.330	1884	0.038	0.967	14.000	14:0	0.71	ECL deviates  0.000	Reference -0.003	
7.778	6843	0.045	----	14.290		----			
8.009	661	0.033	0.960	14.440	15:1 iso G	0.25	ECL deviates  0.000		
8.293	15116	0.038	0.958	14.624	15:0 iso	5.64	ECL deviates  0.001	Reference -0.002	
8.433	8386	0.039	0.957	14.714	15:0 anteiso	3.12	ECL deviates  0.001	Reference -0.002	
8.877	1656	0.040	0.953	15.002	15:0	----	ECL deviates  0.002		
8.970	716	0.036	----	15.058		----			
9.380	495	0.038	----	15.303		----			
9.621	1741	0.054	0.949	15.447	16:1 iso G	0.64	ECL deviates  0.005		
9.920	7384	0.040	0.948	15.626	16:0 iso	2.73	ECL deviates -0.001	Reference -0.003	
10.157	2746	0.053	0.947	15.768	16:1 w9c	1.01	ECL deviates -0.006		
10.239	22626	0.044	0.947	15.817	Sum In Feature 3	8.34	ECL deviates -0.005	16:1 w7c/16:1 w6c	
10.390	7197	0.043	0.947	15.908	16:1 w5c	2.65	ECL deviates -0.001		
10.542	37473	0.042	0.946	15.999	16:0	13.80	ECL deviates -0.001	Reference -0.004	
10.629	648	0.035	----	16.049		----			
11.084	110713	0.059	----	16.312		----			
11.289	44915	0.081	0.945	16.430	Sum In Feature 9	16.52	ECL deviates -0.002	16:0 10-methyl	
11.437	10227	0.108	0.945	16.515	17:1 anteiso w9c	----	> max ar/ht		
11.634	12466	0.060	0.944	16.629	17:0 iso	4.58	ECL deviates -0.001	Reference -0.004	
11.796	10114	0.056	0.944	16.722	17:0 anteiso	3.72	ECL deviates -0.001	Reference -0.003	
11.916	4024	0.062	0.944	16.792	17:1 w8c	1.48	ECL deviates  0.000		
12.083	8825	0.058	0.944	16.888	17:0 cyclo	3.24	ECL deviates  0.000		
12.276	2108	0.044	0.944	16.999	17:0	0.77	ECL deviates -0.001	Reference -0.003	
12.343	3629	0.044	----	17.037		----			
12.463	404	0.039	----	17.105		----			
12.992	1290	0.039	0.944	17.406	17:0 10-methyl	0.47	ECL deviates -0.003		
13.151	469	0.032	----	17.496		----			
13.547	5070	0.043	0.945	17.720	Sum In Feature 5	1.86	ECL deviates  0.000	18:2 w6,9c/18:0 ante	
13.635	21119	0.057	0.945	17.770	18:1 w9c	7.77	ECL deviates  0.001		
13.723	22024	0.050	0.945	17.819	Sum In Feature 8	8.10	ECL deviates -0.004	18:1 w7c	
13.873	3872	0.060	----	17.905		----			
14.034	8848	0.048	0.945	17.996	18:0	3.25	ECL deviates -0.004	Reference -0.005	
14.177	1724	0.047	0.945	18.078	18:1 w7c 11-methyl	0.63	ECL deviates -0.003		
14.602	30412	0.067	----	18.320		----			
14.726	19613	0.088	0.946	18.391	18:0 10-methyl, TBSA	----	> max ar/ht		
15.339	1413	0.055	----	18.742		----		Reference  0.009	
15.436	337	0.039	----	18.797		----			
15.621	16573	0.052	0.947	18.902	19:0 cyclo w8c	6.11	ECL deviates  0.000		
15.868	258378	0.144	----	19.044		----	> max ar/ht		
16.477	1712	0.046	0.947	19.395	20:4 w6,9,12,15c	0.63	ECL deviates  0.000		
17.116	1637	0.051	0.948	19.764	20:1 w9c	0.60	ECL deviates -0.006		
17.518	1018	0.041	0.948	19.996	20:0	0.38	ECL deviates -0.004	Reference -0.005	
17.851	1013	0.040	----	20.188		----	> max rt		
----	22626	---	----	----	Summed Feature 3	8.34	16:1 w7c/16:1 w6c	16:1 w6c/16:1 w7c	
----	5070	---	----	----	Summed Feature 5	1.86	18:2 w6,9c/18:0 ante	18:0 ante/18:2 w6,9c	
----	22024	---	----	----	Summed Feature 8	8.10	18:1 w7c	18:1 w6c	
----	44915	---	----	----	Summed Feature 9	16.52	17:1 iso w9c	16:0 10-methyl	

ECL Deviation: 0.003                            Reference ECL Shift: 0.004      Number Reference Peaks: 12
Total Response: 722510                         Total Named: 271225
Percent Named: 37.54%                         Total Amount: 286695
Profile Comment:   Percent named is less than 85.00.

*** Library match not attempted
