# Supplementary material for: A green garlic (Allium sativum L.) based intercropping system reduces the strain of continuous monocropping in cucumber (Cucumis sativus L.) by adjusting the micro-ecological environment of soil
Source: PeerJ. 2019 Jul 15;7:e7267. doi: 10.7717/peerj.7267 (PMC6637937; doi:10.7717/peerj.7267)
Supplement: Data S1 [file peerj-07-7267-s001.zip › supplemental_Data_S1/15 days after interplanted/CB-3.rtf]

Volume: DATA            File: E131084.29A        Samp Ctr: 25                ID Number: 1004 
Type: Samp                   Bottle: 5                        Method: TSBA6 
Created: 1/8/2013 7:58:40 PM 
Sample ID: 59 


RT	Response	Ar/Ht	RFact	ECL	Peak Name	Percent	Comment1	Comment2	
1.646	4.529E+8	0.028	----	7.007	SOLVENT PEAK	----	< min rt		
1.777	2499	0.024	----	7.265		----	< min rt		
3.360	270	0.028	----	10.272		----			
4.908	642	0.027	1.021	12.096	11:0 iso 3OH	0.26	ECL deviates  0.007		
5.117	1872	0.037	----	12.277		----			
6.807	1467	0.037	0.975	13.620	14:0 iso	0.58	ECL deviates  0.001	Reference -0.002	
7.330	1736	0.035	0.967	14.000	14:0	0.68	ECL deviates  0.000	Reference -0.002	
7.784	5274	0.049	----	14.294		----			
8.011	809	0.035	0.960	14.441	15:1 iso G	0.31	ECL deviates  0.001		
8.293	14976	0.037	0.958	14.623	15:0 iso	5.79	ECL deviates  0.000	Reference -0.002	
8.434	8250	0.039	0.957	14.714	15:0 anteiso	3.19	ECL deviates  0.001	Reference -0.001	
8.876	1735	0.039	0.953	15.000	15:0	----	ECL deviates  0.000		
8.966	704	0.036	----	15.054		----			
9.396	382	0.049	----	15.312		----			
9.616	1714	0.055	0.949	15.443	16:1 iso G	0.66	ECL deviates  0.001		
9.922	7481	0.039	0.948	15.627	16:0 iso	2.86	ECL deviates  0.000	Reference -0.002	
10.156	2515	0.055	0.947	15.767	16:1 w9c	0.96	ECL deviates -0.007		
10.240	22781	0.041	0.947	15.817	Sum In Feature 3	8.71	ECL deviates -0.005	16:1 w7c/16:1 w6c	
10.391	6725	0.041	0.947	15.907	16:1 w5c	2.57	ECL deviates -0.002		
10.543	36492	0.040	0.946	15.998	16:0	13.94	ECL deviates -0.002	Reference -0.003	
11.079	65723	0.064	----	16.308		----			
11.290	36878	0.081	0.945	16.429	Sum In Feature 9	14.06	ECL deviates -0.003	16:0 10-methyl	
11.452	8565	0.086	0.945	16.523	17:1 anteiso w9c	----	> max ar/ht		
11.635	9342	0.050	0.944	16.629	17:0 iso	3.56	ECL deviates -0.001	Reference -0.003	
11.797	9636	0.059	0.944	16.722	17:0 anteiso	3.67	ECL deviates -0.001	Reference -0.002	
11.917	3020	0.058	0.944	16.791	17:1 w8c	1.15	ECL deviates -0.001		
12.084	7532	0.052	0.944	16.888	17:0 cyclo	2.87	ECL deviates  0.000		
12.276	2093	0.054	0.944	16.998	17:0	0.80	ECL deviates -0.002	Reference -0.003	
12.345	3672	0.045	----	17.038		----			
12.994	1867	0.049	0.944	17.406	17:0 10-methyl	0.71	ECL deviates -0.003		
13.150	973	0.042	----	17.494		----			
13.548	4753	0.046	0.945	17.720	Sum In Feature 5	1.81	ECL deviates  0.000	18:2 w6,9c/18:0 ante	
13.634	20393	0.059	0.945	17.768	18:1 w9c	7.78	ECL deviates -0.001		
13.724	21438	0.050	0.945	17.820	Sum In Feature 8	8.17	ECL deviates -0.003	18:1 w7c	
13.875	3521	0.059	----	17.905		----			
14.038	8276	0.047	0.945	17.998	18:0	3.16	ECL deviates -0.002	Reference -0.003	
14.180	2086	0.055	0.945	18.079	18:1 w7c 11-methyl	0.80	ECL deviates -0.002		
14.606	11569	0.063	----	18.322		----			
14.727	8668	0.056	0.946	18.392	18:0 10-methyl, TBSA	3.31	ECL deviates  0.000		
14.786	4428	0.055	----	18.425		----			
15.340	1028	0.046	----	18.742		----		Reference  0.009	
15.620	15589	0.050	0.947	18.901	19:0 cyclo w8c	5.96	ECL deviates -0.001		
15.875	259679	0.151	----	19.048		----	> max ar/ht		
16.478	1403	0.042	0.947	19.396	20:4 w6,9,12,15c	0.54	ECL deviates  0.001		
17.120	1908	0.054	0.948	19.767	20:1 w9c	0.73	ECL deviates -0.003		
17.516	1108	0.047	0.948	19.995	20:0	0.42	ECL deviates -0.005	Reference -0.005	
17.848	1003	0.036	----	20.187		----	> max rt		
18.186	1255	0.081	----	20.382		----	> max rt		
----	22781	---	----	----	Summed Feature 3	8.71	16:1 w7c/16:1 w6c	16:1 w6c/16:1 w7c	
----	4753	---	----	----	Summed Feature 5	1.81	18:2 w6,9c/18:0 ante	18:0 ante/18:2 w6,9c	
----	21438	---	----	----	Summed Feature 8	8.17	18:1 w7c	18:1 w6c	
----	36878	---	----	----	Summed Feature 9	14.06	17:1 iso w9c	16:0 10-methyl	

ECL Deviation: 0.003                            Reference ECL Shift: 0.004      Number Reference Peaks: 12
Total Response: 629236                         Total Named: 261576
Percent Named: 41.57%                         Total Amount: 257501
Profile Comment:   Percent named is less than 85.00.

*** Library match not attempted
