# Supplementary material for: A green garlic (Allium sativum L.) based intercropping system reduces the strain of continuous monocropping in cucumber (Cucumis sativus L.) by adjusting the micro-ecological environment of soil
Source: PeerJ. 2019 Jul 15;7:e7267. doi: 10.7717/peerj.7267 (PMC6637937; doi:10.7717/peerj.7267)
Supplement: Data S1 [file peerj-07-7267-s001.zip › supplemental_Data_S1/15 days after interplanted/CR-1.rtf]

Volume: DATA            File: E131084.29A        Samp Ctr: 20                ID Number: 1008 
Type: Samp                   Bottle: 6                        Method: TSBA6 
Created: 1/8/2013 5:57:36 PM 
Sample ID: 54 


RT	Response	Ar/Ht	RFact	ECL	Peak Name	Percent	Comment1	Comment2	
1.646	4.514E+8	0.029	----	7.001	SOLVENT PEAK	----	< min rt		
1.777	473	0.010	----	7.260		----	< min rt		
2.794	224	0.023	----	9.256		----			
3.356	434	0.030	----	10.262		----			
4.908	1123	0.028	1.021	12.097	11:0 iso 3OH	0.51	ECL deviates  0.008		
5.115	2719	0.039	----	12.275		----			
6.806	1584	0.035	0.975	13.619	14:0 iso	0.68	ECL deviates  0.000	Reference -0.003	
7.329	2198	0.035	0.967	13.999	14:0	0.94	ECL deviates -0.001	Reference -0.003	
7.779	6229	0.049	----	14.291		----			
8.011	825	0.039	0.960	14.441	15:1 iso G	0.35	ECL deviates  0.001		
8.293	14726	0.038	0.958	14.624	15:0 iso	6.23	ECL deviates  0.001	Reference -0.002	
8.433	8438	0.039	0.957	14.714	15:0 anteiso	3.57	ECL deviates  0.001	Reference -0.001	
8.637	482	0.039	0.955	14.846	15:1 w6c	0.20	ECL deviates -0.010		
8.877	2812	0.038	0.953	15.001	15:0	----	ECL deviates  0.001		
8.969	769	0.035	----	15.056		----			
9.616	1647	0.055	0.949	15.444	16:1 iso G	0.69	ECL deviates  0.002		
9.921	7309	0.041	0.948	15.627	16:0 iso	3.06	ECL deviates  0.000	Reference -0.003	
10.161	3161	0.047	0.947	15.770	16:1 w9c	1.32	ECL deviates -0.004		
10.240	25645	0.042	0.947	15.818	Sum In Feature 3	10.73	ECL deviates -0.004	16:1 w7c/16:1 w6c	
10.391	7462	0.041	0.947	15.908	16:1 w5c	3.12	ECL deviates -0.001		
10.543	56472	0.042	0.946	15.999	16:0	23.62	ECL deviates -0.001	Reference -0.003	
11.081	75212	0.060	----	16.310		----			
11.289	35093	0.083	0.945	16.430	Sum In Feature 9	14.65	ECL deviates -0.002	16:0 10-methyl	
11.634	5747	0.040	0.944	16.629	17:0 iso	2.40	ECL deviates -0.001	Reference -0.003	
11.796	5868	0.043	0.944	16.723	17:0 anteiso	2.45	ECL deviates  0.000	Reference -0.003	
11.919	1985	0.043	0.944	16.794	17:1 w8c	0.83	ECL deviates  0.002		
12.084	6375	0.051	0.944	16.889	17:0 cyclo	2.66	ECL deviates  0.001		
12.278	2350	0.050	0.944	17.001	17:0	0.98	ECL deviates  0.001	Reference -0.002	
12.346	3007	0.041	0.944	17.040	16:1 2OH	1.25	ECL deviates -0.008		
12.992	2115	0.048	0.944	17.406	17:0 10-methyl	0.88	ECL deviates -0.003		
13.141	889	0.050	----	17.491		----			
13.548	7499	0.048	0.945	17.722	Sum In Feature 5	3.13	ECL deviates  0.002	18:2 w6,9c/18:0 ante	
13.677	84466	0.074	----	17.795		----			
13.880	3223	0.053	0.945	17.911	18:1 w5c	1.35	ECL deviates -0.008		
14.038	8928	0.048	0.945	18.000	18:0	3.73	ECL deviates  0.000	Reference -0.004	
14.178	1938	0.047	0.945	18.080	18:1 w7c 11-methyl	0.81	ECL deviates -0.001		
14.603	14832	0.066	----	18.323		----			
14.723	13143	0.084	0.946	18.392	18:0 10-methyl, TBSA	----	> max ar/ht		
15.349	915	0.046	0.946	18.750	Sum In Feature 6	0.38	ECL deviates -0.006	19:1 w11c/19:1 w9c	
15.619	15446	0.050	0.947	18.905	19:0 cyclo w8c	6.46	ECL deviates  0.003		
15.864	265408	0.152	----	19.045		----	> max ar/ht		
16.479	3012	0.050	0.947	19.401	20:4 w6,9,12,15c	1.26	ECL deviates  0.006		
16.606	923	0.039	----	19.475		----			
17.116	3037	0.072	0.948	19.769	20:1 w9c	1.27	ECL deviates -0.001		
17.514	1116	0.043	0.948	20.000	20:0	0.47	ECL deviates  0.000	Reference -0.007	
17.846	1084	0.043	----	20.191		----	> max rt		
18.178	892	0.059	----	20.384		----	> max rt		
----	25645	---	----	----	Summed Feature 3	10.73	16:1 w7c/16:1 w6c	16:1 w6c/16:1 w7c	
----	7499	---	----	----	Summed Feature 5	3.13	18:2 w6,9c/18:0 ante	18:0 ante/18:2 w6,9c	
----	915	---	----	----	Summed Feature 6	0.38	19:1 w11c/19:1 w9c	19:1 w9c/19:1 w11c	
----	35093	---	----	----	Summed Feature 9	14.65	17:1 iso w9c	16:0 10-methyl	

ECL Deviation: 0.004                            Reference ECL Shift: 0.003      Number Reference Peaks: 11
Total Response: 703973                         Total Named: 238725
Percent Named: 33.91%                         Total Amount: 241377
Profile Comment:   Percent named is less than 85.00.

*** Library match not attempted
