# Supplementary material for: A green garlic (Allium sativum L.) based intercropping system reduces the strain of continuous monocropping in cucumber (Cucumis sativus L.) by adjusting the micro-ecological environment of soil
Source: PeerJ. 2019 Jul 15;7:e7267. doi: 10.7717/peerj.7267 (PMC6637937; doi:10.7717/peerj.7267)
Supplement: Data S1 [file peerj-07-7267-s001.zip › supplemental_Data_S1/15 days after interplanted/CR-2.rtf]

Volume: DATA            File: E131084.29A        Samp Ctr: 21                ID Number: 1009 
Type: Samp                   Bottle: 7                        Method: TSBA6 
Created: 1/8/2013 6:21:47 PM 
Sample ID: 55 


RT	Response	Ar/Ht	RFact	ECL	Peak Name	Percent	Comment1	Comment2	
1.646	4.518E+8	0.028	----	7.006	SOLVENT PEAK	----	< min rt		
1.778	2635	0.023	----	7.265		----	< min rt		
4.910	821	0.033	1.021	12.104	11:0 iso 3OH	0.36	ECL deviates  0.015		
5.128	1152	0.041	----	12.292		----			
5.499	363	0.031	1.002	12.611	13:0 iso	0.15	ECL deviates -0.003	Reference -0.010	
6.806	1716	0.035	0.975	13.621	14:0 iso	0.71	ECL deviates  0.002	Reference -0.002	
7.329	2024	0.037	0.967	14.001	14:0	0.83	ECL deviates  0.001	Reference -0.003	
7.794	3229	0.049	----	14.301		----			
8.011	1025	0.040	0.960	14.442	15:1 iso G	0.42	ECL deviates  0.002		
8.293	15591	0.038	0.958	14.624	15:0 iso	6.35	ECL deviates  0.001	Reference -0.002	
8.433	8949	0.039	0.957	14.714	15:0 anteiso	3.64	ECL deviates  0.001	Reference -0.001	
8.646	644	0.047	0.955	14.852	15:1 w6c	0.26	ECL deviates -0.004		
8.877	2818	0.038	0.953	15.002	15:0	----	ECL deviates  0.002		
8.965	675	0.036	----	15.054		----			
9.624	2033	0.061	0.949	15.448	16:1 iso G	0.82	ECL deviates  0.006		
9.921	7746	0.040	0.948	15.626	16:0 iso	3.12	ECL deviates -0.001	Reference -0.003	
10.160	3439	0.052	0.947	15.769	16:1 w9c	1.39	ECL deviates -0.005		
10.241	26328	0.043	0.947	15.818	Sum In Feature 3	10.61	ECL deviates -0.004	16:1 w7c/16:1 w6c	
10.391	8075	0.043	0.947	15.908	16:1 w5c	3.25	ECL deviates -0.001		
10.544	59528	0.042	0.946	15.999	16:0	23.96	ECL deviates -0.001	Reference -0.003	
10.638	549	0.042	----	16.053		----			
11.084	31879	0.063	----	16.311		----			
11.289	26762	0.068	0.945	16.429	Sum In Feature 9	10.76	ECL deviates -0.003	16:0 10-methyl	
11.635	5993	0.042	0.944	16.629	17:0 iso	2.41	ECL deviates -0.001	Reference -0.003	
11.796	6163	0.043	0.944	16.722	17:0 anteiso	2.48	ECL deviates -0.001	Reference -0.003	
11.917	2161	0.046	0.944	16.792	17:1 w8c	0.87	ECL deviates  0.000		
12.086	6999	0.052	0.944	16.890	17:0 cyclo	2.81	ECL deviates  0.002		
12.278	2247	0.042	0.944	17.001	17:0	0.90	ECL deviates  0.001	Reference -0.002	
12.347	3453	0.045	0.944	17.040	16:1 2OH	1.39	ECL deviates -0.008		
12.993	2074	0.044	0.944	17.407	17:0 10-methyl	0.83	ECL deviates -0.002		
13.150	1277	0.059	----	17.496		----			
13.548	8082	0.046	0.945	17.722	Sum In Feature 5	3.25	ECL deviates  0.002	18:2 w6,9c/18:0 ante	
13.677	89920	0.075	----	17.795		----			
13.876	3725	0.060	0.945	17.908	18:1 w5c	1.50	ECL deviates -0.011		
14.034	9661	0.045	0.945	17.998	18:0	3.88	ECL deviates -0.002	Reference -0.005	
14.181	2097	0.043	0.945	18.082	18:1 w7c 11-methyl	0.84	ECL deviates  0.001		
14.615	4747	0.062	----	18.331		----			
14.727	7132	0.057	0.946	18.395	18:0 10-methyl, TBSA	2.87	ECL deviates  0.003		
14.788	3371	0.043	----	18.430		----			
15.343	813	0.038	0.946	18.747	Sum In Feature 6	0.33	ECL deviates -0.009	19:1 w11c/19:1 w9c	
15.622	16292	0.051	0.947	18.907	19:0 cyclo w8c	6.56	ECL deviates  0.005		
15.887	281395	0.150	----	19.060		----	> max ar/ht		
16.478	2842	0.047	0.947	19.402	20:4 w6,9,12,15c	1.15	ECL deviates  0.007		
16.609	791	0.035	----	19.478		----			
17.118	1936	0.056	0.948	19.773	20:1 w9c	0.78	ECL deviates  0.003		
17.512	1288	0.041	0.948	20.001	20:0	0.52	ECL deviates  0.001	Reference -0.008	
17.856	889	0.051	----	20.200		----	> max rt		
----	26328	---	----	----	Summed Feature 3	10.61	16:1 w7c/16:1 w6c	16:1 w6c/16:1 w7c	
----	8082	---	----	----	Summed Feature 5	3.25	18:2 w6,9c/18:0 ante	18:0 ante/18:2 w6,9c	
----	813	---	----	----	Summed Feature 6	0.33	19:1 w11c/19:1 w9c	19:1 w9c/19:1 w11c	
----	26762	---	----	----	Summed Feature 9	10.76	17:1 iso w9c	16:0 10-methyl	

ECL Deviation: 0.005                            Reference ECL Shift: 0.004      Number Reference Peaks: 12
Total Response: 666989                         Total Named: 248004
Percent Named: 37.18%                         Total Amount: 237754
Profile Comment:   Percent named is less than 85.00.

*** Library match not attempted
