# Supplementary material for: A green garlic (Allium sativum L.) based intercropping system reduces the strain of continuous monocropping in cucumber (Cucumis sativus L.) by adjusting the micro-ecological environment of soil
Source: PeerJ. 2019 Jul 15;7:e7267. doi: 10.7717/peerj.7267 (PMC6637937; doi:10.7717/peerj.7267)
Supplement: Data S1 [file peerj-07-7267-s001.zip › supplemental_Data_S1/15 days after interplanted/CR-3.rtf]

Volume: DATA            File: E131084.29A        Samp Ctr: 22                ID Number: 1001 
Type: Samp                   Bottle: 2                        Method: TSBA6 
Created: 1/8/2013 6:45:57 PM 
Sample ID: 56 


RT	Response	Ar/Ht	RFact	ECL	Peak Name	Percent	Comment1	Comment2	
1.646	4.512E+8	0.029	----	7.000	SOLVENT PEAK	----	< min rt		
1.778	2746	0.025	----	7.259		----	< min rt		
3.058	247	0.025	----	9.775		----			
4.907	1117	0.033	1.021	12.095	11:0 iso 3OH	0.49	ECL deviates  0.006		
6.807	1692	0.034	0.975	13.619	14:0 iso	0.70	ECL deviates  0.000	Reference -0.002	
7.330	2002	0.036	0.967	13.999	14:0	0.83	ECL deviates -0.001	Reference -0.003	
7.811	1822	0.042	----	14.311		----			
8.008	779	0.036	0.960	14.438	15:1 iso G	0.32	ECL deviates -0.002		
8.293	14741	0.038	0.958	14.623	15:0 iso	6.03	ECL deviates  0.000	Reference -0.002	
8.435	8574	0.040	0.957	14.715	15:0 anteiso	3.50	ECL deviates  0.002	Reference  0.000	
8.877	2473	0.039	0.953	15.001	15:0	----	ECL deviates  0.001		
8.971	761	0.038	----	15.057		----			
9.625	1843	0.060	0.949	15.449	16:1 iso G	0.75	ECL deviates  0.007		
9.921	7603	0.039	0.948	15.627	16:0 iso	3.08	ECL deviates  0.000	Reference -0.003	
10.158	2621	0.049	0.947	15.768	16:1 w9c	1.06	ECL deviates -0.006		
10.240	24935	0.045	0.947	15.817	Sum In Feature 3	10.09	ECL deviates -0.005	16:1 w7c/16:1 w6c	
10.392	7486	0.042	0.947	15.908	16:1 w5c	3.03	ECL deviates -0.001		
10.543	54758	0.041	0.946	15.999	16:0	22.13	ECL deviates -0.001	Reference -0.003	
11.107	8608	0.075	----	16.325		----			
11.289	29654	0.073	0.945	16.430	Sum In Feature 9	11.97	ECL deviates -0.002	16:0 10-methyl	
11.444	4348	0.070	0.945	16.520	17:1 anteiso w9c	1.75	ECL deviates -0.004		
11.636	7271	0.046	0.944	16.630	17:0 iso	2.93	ECL deviates  0.000	Reference -0.002	
11.797	7174	0.050	0.944	16.724	17:0 anteiso	2.89	ECL deviates  0.001	Reference -0.002	
11.919	2860	0.052	0.944	16.794	17:1 w8c	1.15	ECL deviates  0.002		
12.087	6966	0.053	0.944	16.891	17:0 cyclo	2.81	ECL deviates  0.003		
12.276	2077	0.046	0.944	17.000	17:0	0.84	ECL deviates  0.000	Reference -0.003	
12.346	2651	0.041	0.944	17.040	16:1 2OH	1.07	ECL deviates -0.008		
12.998	1550	0.040	0.944	17.410	17:0 10-methyl	0.63	ECL deviates  0.001		
13.145	759	0.042	----	17.494		----			
13.547	7604	0.046	0.945	17.721	Sum In Feature 5	3.07	ECL deviates  0.001	18:2 w6,9c/18:0 ante	
13.678	81842	0.075	----	17.796		----			
13.881	3883	0.057	0.945	17.911	18:1 w5c	1.57	ECL deviates -0.008		
14.037	9275	0.046	0.945	18.000	18:0	3.74	ECL deviates  0.000	Reference -0.004	
14.178	1754	0.043	0.945	18.080	18:1 w7c 11-methyl	0.71	ECL deviates -0.001		
14.726	5963	0.060	0.946	18.394	18:0 10-methyl, TBSA	2.41	ECL deviates  0.002		
14.785	2854	0.045	----	18.428		----			
15.346	1672	0.062	0.946	18.749	Sum In Feature 6	0.68	ECL deviates -0.007	19:1 w11c/19:1 w9c	
15.621	16998	0.051	0.947	18.906	19:0 cyclo w8c	6.87	ECL deviates  0.004		
15.874	260495	0.149	----	19.051		----	> max ar/ht		
16.478	3262	0.046	0.947	19.401	20:4 w6,9,12,15c	1.32	ECL deviates  0.006		
16.607	863	0.039	----	19.475		----			
17.118	2860	0.077	0.948	19.771	20:1 w9c	1.16	ECL deviates  0.001		
17.515	1024	0.033	0.948	20.000	20:0	0.41	ECL deviates  0.000	Reference -0.006	
17.851	780	0.038	----	20.195		----	> max rt		
----	24935	---	----	----	Summed Feature 3	10.09	16:1 w7c/16:1 w6c	16:1 w6c/16:1 w7c	
----	7604	---	----	----	Summed Feature 5	3.07	18:2 w6,9c/18:0 ante	18:0 ante/18:2 w6,9c	
----	1672	---	----	----	Summed Feature 6	0.68	19:1 w11c/19:1 w9c	19:1 w9c/19:1 w11c	
----	29654	---	----	----	Summed Feature 9	11.97	17:1 iso w9c	16:0 10-methyl	

ECL Deviation: 0.004                            Reference ECL Shift: 0.003      Number Reference Peaks: 11
Total Response: 605249                         Total Named: 246999
Percent Named: 40.81%                         Total Amount: 236440
Profile Comment:   Percent named is less than 85.00.

*** Library match not attempted
