# Supplementary material for: A green garlic (Allium sativum L.) based intercropping system reduces the strain of continuous monocropping in cucumber (Cucumis sativus L.) by adjusting the micro-ecological environment of soil
Source: PeerJ. 2019 Jul 15;7:e7267. doi: 10.7717/peerj.7267 (PMC6637937; doi:10.7717/peerj.7267)
Supplement: Data S1 [file peerj-07-7267-s001.zip › supplemental_Data_S1/15 days after interplanted/GB-1.rtf]

Volume: DATA            File: E131094.42A        Samp Ctr: 7                  ID Number: 1009 
Type: Samp                   Bottle: 7                        Method: TSBA6 
Created: 1/9/2013 1:00:19 PM 
Sample ID: 65 


RT	Response	Ar/Ht	RFact	ECL	Peak Name	Percent	Comment1	Comment2	
1.645	4.531E+8	0.029	----	7.007	SOLVENT PEAK	----	< min rt		
1.779	12095	0.023	----	7.269		----	< min rt		
1.833	814	0.036	----	7.376		----	< min rt		
2.033	410	0.021	----	7.768		----	< min rt		
2.396	178	0.022	----	8.480		----	< min rt		
3.056	204	0.023	----	9.778		----			
3.356	287	0.028	----	10.267		----			
4.406	345	0.034	----	11.581		----			
4.782	562	0.045	1.020	11.987	12:0	0.21	ECL deviates -0.013		
4.907	2423	0.033	1.017	12.098	11:0 iso 3OH	0.92	ECL deviates  0.009		
5.113	2261	0.036	----	12.276		----			
5.308	291	0.028	1.004	12.444	11:0 3OH	0.11	ECL deviates  0.006		
5.504	412	0.032	0.998	12.613	13:0 iso	0.15	ECL deviates -0.001	Reference -0.005	
6.806	1460	0.035	0.972	13.620	14:0 iso	0.53	ECL deviates  0.001	Reference -0.002	
7.329	2057	0.039	0.965	14.000	14:0	0.74	ECL deviates  0.000	Reference -0.002	
7.778	6375	0.047	----	14.290		----			
8.008	967	0.042	0.958	14.439	15:1 iso G	0.35	ECL deviates -0.001		
8.293	15525	0.040	0.956	14.624	15:0 iso	5.56	ECL deviates  0.001	Reference -0.001	
8.432	8867	0.039	0.955	14.714	15:0 anteiso	3.17	ECL deviates  0.001	Reference -0.001	
8.873	1805	0.038	0.952	14.999	15:0	----	ECL deviates -0.001		
8.967	643	0.031	----	15.056		----			
9.631	1912	0.065	0.949	15.453	16:1 iso H	0.68	ECL deviates -0.008		
9.921	7880	0.041	0.948	15.627	16:0 iso	2.80	ECL deviates  0.000	Reference -0.002	
10.084	449	0.029	0.948	15.724	16:0 anteiso	0.16	ECL deviates  0.006		
10.164	2495	0.043	0.948	15.772	16:1 w9c	0.88	ECL deviates -0.002		
10.240	20958	0.046	0.947	15.818	Sum In Feature 3	7.43	ECL deviates -0.004	16:1 w7c/16:1 w6c	
10.391	6700	0.041	0.947	15.909	16:1 w5c	2.38	ECL deviates  0.000		
10.542	39009	0.040	0.947	15.999	16:0	13.83	ECL deviates -0.001	Reference -0.003	
11.085	102151	0.059	----	16.312		----			
11.289	39864	0.083	0.946	16.430	Sum In Feature 9	14.12	ECL deviates -0.002	16:0 10-methyl	
11.636	5951	0.042	0.946	16.631	17:0 iso	2.11	ECL deviates  0.001	Reference -0.001	
11.797	6102	0.045	0.946	16.724	17:0 anteiso	2.16	ECL deviates  0.001	Reference -0.001	
11.921	1783	0.044	0.946	16.795	17:1 w8c	0.63	ECL deviates  0.003		
12.084	6220	0.049	0.946	16.890	17:0 cyclo	2.20	ECL deviates  0.002		
12.274	1365	0.036	0.946	17.000	17:0	0.48	ECL deviates  0.000	Reference -0.002	
12.344	2708	0.038	0.946	17.039	16:1 2OH	0.96	ECL deviates -0.009		
12.995	1585	0.044	0.947	17.408	17:0 10-methyl	0.56	ECL deviates -0.001		
13.145	903	0.042	----	17.493		----			
13.548	12333	0.046	0.948	17.722	Sum In Feature 5	4.38	ECL deviates  0.002	18:2 w6,9c/18:0 ante	
13.633	22525	0.059	0.948	17.770	18:1 w9c	7.99	ECL deviates  0.001		
13.724	23035	0.050	0.948	17.821	Sum In Feature 8	8.17	ECL deviates -0.002	18:1 w7c	
13.878	2908	0.060	0.948	17.908	18:1 w5c	1.03	ECL deviates -0.011		
14.035	7706	0.044	0.948	17.998	18:0	2.74	ECL deviates -0.002	Reference -0.005	
14.180	1831	0.044	0.949	18.081	18:1 w7c 11-methyl	0.65	ECL deviates  0.000		
14.603	29058	0.064	----	18.323		----			
14.727	12193	0.054	0.949	18.393	18:0 10-methyl, TBSA	4.33	ECL deviates  0.001		
14.785	6225	0.044	----	18.427		----			
15.344	1052	0.048	0.950	18.747	Sum In Feature 6	0.37	ECL deviates -0.009	19:1 w11c/19:1 w9c	
15.620	16942	0.050	0.951	18.905	19:0 cyclo w8c	6.03	ECL deviates  0.003		
15.880	264471	0.156	----	19.054		----	> max ar/ht		
16.477	1168	0.041	0.952	19.399	20:4 w6,9,12,15c	0.42	ECL deviates  0.004		
17.120	959	0.033	0.952	19.771	20:1 w9c	0.34	ECL deviates  0.001		
17.518	1141	0.049	0.952	20.001	20:0	0.41	ECL deviates  0.001	Reference -0.003	
17.852	938	0.043	----	20.194		----	> max rt		
18.397	3865	0.112	----	20.509		----	> max rt		
----	20958	---	----	----	Summed Feature 3	7.43	16:1 w7c/16:1 w6c	16:1 w6c/16:1 w7c	
----	12333	---	----	----	Summed Feature 5	4.38	18:2 w6,9c/18:0 ante	18:0 ante/18:2 w6,9c	
----	1052	---	----	----	Summed Feature 6	0.37	19:1 w11c/19:1 w9c	19:1 w9c/19:1 w11c	
----	23035	---	----	----	Summed Feature 8	8.17	18:1 w7c	18:1 w6c	
----	39864	---	----	----	Summed Feature 9	14.12	17:1 iso w9c	16:0 10-methyl	

ECL Deviation: 0.004                            Reference ECL Shift: 0.003      Number Reference Peaks: 12
Total Response: 694262                         Total Named: 281337
Percent Named: 40.52%                         Total Amount: 268824
Profile Comment:   Percent named is less than 85.00.

*** Library match not attempted
