# Supplementary material for: A green garlic (Allium sativum L.) based intercropping system reduces the strain of continuous monocropping in cucumber (Cucumis sativus L.) by adjusting the micro-ecological environment of soil
Source: PeerJ. 2019 Jul 15;7:e7267. doi: 10.7717/peerj.7267 (PMC6637937; doi:10.7717/peerj.7267)
Supplement: Data S1 [file peerj-07-7267-s001.zip › supplemental_Data_S1/15 days after interplanted/GB-2.rtf]

Volume: DATA            File: E131094.42A        Samp Ctr: 9                  ID Number: 1012 
Type: Samp                   Bottle: 8                        Method: TSBA6 
Created: 1/9/2013 1:49:00 PM 
Sample ID: 66 


RT	Response	Ar/Ht	RFact	ECL	Peak Name	Percent	Comment1	Comment2	
1.646	4.542E+8	0.029	----	7.010	SOLVENT PEAK	----	< min rt		
1.779	12431	0.022	----	7.272		----	< min rt		
1.835	492	0.043	----	7.381		----	< min rt		
2.033	454	0.022	----	7.769		----	< min rt		
2.156	201	0.024	----	8.011		----	< min rt		
2.286	221	0.027	----	8.266		----	< min rt		
2.394	175	0.019	----	8.479		----	< min rt		
3.060	538	0.023	----	9.787		----			
3.352	368	0.024	----	10.262		----			
4.006	330	0.029	1.059	11.151	10:0 2OH	0.13	ECL deviates -0.002		
4.408	784	0.041	----	11.585		----			
4.780	597	0.036	1.020	11.985	12:0	0.22	ECL deviates -0.015		
4.908	2555	0.034	1.017	12.099	11:0 iso 3OH	0.95	ECL deviates  0.010		
5.113	3474	0.035	----	12.276		----			
5.314	201	0.024	----	12.450		----			
5.502	388	0.027	0.998	12.612	13:0 iso	0.14	ECL deviates -0.002	Reference -0.006	
6.806	1708	0.037	0.972	13.620	14:0 iso	0.61	ECL deviates  0.001	Reference -0.001	
7.331	2104	0.035	0.965	14.001	14:0	0.74	ECL deviates  0.001	Reference -0.001	
7.778	8584	0.046	----	14.291		----			
8.008	832	0.035	0.958	14.439	15:1 iso G	0.29	ECL deviates -0.001		
8.293	15891	0.038	0.956	14.624	15:0 iso	5.55	ECL deviates  0.001	Reference -0.001	
8.433	9375	0.037	0.955	14.714	15:0 anteiso	3.27	ECL deviates  0.001	Reference -0.001	
8.876	1974	0.036	0.952	15.001	15:0	----	ECL deviates  0.001		
8.965	611	0.032	----	15.054		----			
9.635	1753	0.055	0.949	15.455	16:1 iso H	0.61	ECL deviates -0.006		
9.922	8312	0.041	0.948	15.627	16:0 iso	2.88	ECL deviates  0.000	Reference -0.001	
10.160	3043	0.054	0.948	15.770	16:1 w9c	1.05	ECL deviates -0.004		
10.239	22491	0.042	0.947	15.817	Sum In Feature 3	7.79	ECL deviates -0.005	16:1 w7c/16:1 w6c	
10.391	7254	0.042	0.947	15.908	16:1 w5c	2.51	ECL deviates -0.001		
10.543	42636	0.042	0.947	15.999	16:0	14.76	ECL deviates -0.001	Reference -0.002	
10.628	549	0.037	----	16.048		----			
11.084	112792	0.057	----	16.312		----			
11.287	44278	0.086	0.946	16.428	Sum In Feature 9	15.31	ECL deviates -0.004	16:0 10-methyl	
11.634	6168	0.042	0.946	16.629	17:0 iso	2.13	ECL deviates -0.001	Reference -0.002	
11.796	6335	0.047	0.946	16.723	17:0 anteiso	2.19	ECL deviates  0.000	Reference -0.001	
11.920	1926	0.048	0.946	16.794	17:1 w8c	0.67	ECL deviates  0.002		
12.085	5963	0.045	0.946	16.889	17:0 cyclo	2.06	ECL deviates  0.001		
12.275	1323	0.040	0.946	16.999	17:0	0.46	ECL deviates -0.001	Reference -0.002	
12.345	3143	0.045	0.946	17.039	16:1 2OH	1.09	ECL deviates -0.009		
12.995	1783	0.046	0.947	17.407	17:0 10-methyl	0.62	ECL deviates -0.002		
13.146	844	0.039	----	17.492		----			
13.547	13223	0.047	0.948	17.720	Sum In Feature 5	4.58	ECL deviates  0.000	18:2 w6,9c/18:0 ante	
13.637	24881	0.060	0.948	17.771	18:1 w9c	8.62	ECL deviates  0.002		
13.725	25176	0.048	0.948	17.821	Sum In Feature 8	8.72	ECL deviates -0.002	18:1 w7c	
13.876	3133	0.053	----	17.906		----			
14.035	8695	0.048	0.948	17.996	18:0	3.01	ECL deviates -0.004	Reference -0.005	
14.182	2927	0.060	0.949	18.080	18:1 w7c 11-methyl	1.02	ECL deviates -0.001		
14.604	27034	0.063	----	18.322		----			
14.727	18540	0.081	0.949	18.392	18:0 10-methyl, TBSA	----	> max ar/ht		
15.329	948	0.048	0.950	18.737	19:0 anteiso	0.33	ECL deviates  0.006	Reference  0.004	
15.622	17868	0.051	0.951	18.904	19:0 cyclo w8c	6.21	ECL deviates  0.002		
15.875	282438	0.144	----	19.049		----	> max ar/ht		
16.477	1331	0.043	0.952	19.398	20:4 w6,9,12,15c	0.46	ECL deviates  0.003		
16.997	196	0.021	----	19.698		----			
17.119	1702	0.055	0.952	19.768	20:1 w9c	0.59	ECL deviates -0.002		
17.518	1191	0.043	0.952	19.999	20:0	0.41	ECL deviates -0.001	Reference -0.003	
17.849	867	0.037	----	20.190		----	> max rt		
----	22491	---	----	----	Summed Feature 3	7.79	16:1 w7c/16:1 w6c	16:1 w6c/16:1 w7c	
----	13223	---	----	----	Summed Feature 5	4.58	18:2 w6,9c/18:0 ante	18:0 ante/18:2 w6,9c	
----	25176	---	----	----	Summed Feature 8	8.72	18:1 w7c	18:1 w6c	
----	44278	---	----	----	Summed Feature 9	15.31	17:1 iso w9c	16:0 10-methyl	

ECL Deviation: 0.004                            Reference ECL Shift: 0.003      Number Reference Peaks: 13
Total Response: 748214                         Total Named: 288130
Percent Named: 38.51%                         Total Amount: 293042
Profile Comment:   Percent named is less than 85.00.

*** Library match not attempted
