# Supplementary material for: A green garlic (Allium sativum L.) based intercropping system reduces the strain of continuous monocropping in cucumber (Cucumis sativus L.) by adjusting the micro-ecological environment of soil
Source: PeerJ. 2019 Jul 15;7:e7267. doi: 10.7717/peerj.7267 (PMC6637937; doi:10.7717/peerj.7267)
Supplement: Data S1 [file peerj-07-7267-s001.zip › supplemental_Data_S1/15 days after interplanted/GB-3.rtf]

Volume: DATA            File: E131095.94A        Samp Ctr: 4                  ID Number: 1012 
Type: Samp                   Bottle: 2                        Method: TSBA6 
Created: 1/9/2013 3:26:22 PM 
Sample ID: 67 


RT	Response	Ar/Ht	RFact	ECL	Peak Name	Percent	Comment1	Comment2	
1.646	4.54E+8	0.029	----	7.007	SOLVENT PEAK	----	< min rt		
1.778	6273	0.023	----	7.267		----	< min rt		
2.032	280	0.028	----	7.766		----	< min rt		
2.801	193	0.025	----	9.275		----			
3.058	306	0.029	----	9.780		----			
3.358	414	0.029	----	10.272		----			
4.406	546	0.036	----	11.580		----			
4.797	597	0.040	1.022	12.002	12:0	0.23	ECL deviates  0.002	Reference -0.001	
4.906	2117	0.032	1.019	12.096	11:0 iso 3OH	0.83	ECL deviates  0.007		
5.115	2486	0.037	----	12.277		----			
5.502	454	0.033	1.000	12.610	13:0 iso	0.17	ECL deviates -0.004	Reference -0.007	
6.409	461	0.046	----	13.332		----			
6.805	1541	0.034	0.974	13.619	14:0 iso	0.57	ECL deviates  0.000	Reference -0.003	
7.330	2165	0.038	0.966	14.000	14:0	0.80	ECL deviates  0.000	Reference -0.002	
7.782	6508	0.051	----	14.293		----			
8.012	839	0.033	0.959	14.442	15:1 iso G	0.31	ECL deviates  0.002		
8.292	15291	0.039	0.957	14.623	15:0 iso	5.61	ECL deviates  0.000	Reference -0.002	
8.433	8732	0.040	0.956	14.715	15:0 anteiso	3.20	ECL deviates  0.002	Reference -0.001	
8.633	368	0.036	----	14.844		----			
8.875	1933	0.039	0.953	15.000	15:0	----	ECL deviates  0.000		
8.967	765	0.032	----	15.056		----			
9.630	1951	0.068	0.949	15.453	16:1 iso H	0.71	ECL deviates -0.008		
9.921	7799	0.040	0.948	15.627	16:0 iso	2.83	ECL deviates  0.000	Reference -0.003	
10.082	350	0.029	0.948	15.723	16:0 anteiso	0.13	ECL deviates  0.005		
10.163	2705	0.044	0.947	15.772	16:1 w9c	0.98	ECL deviates -0.002		
10.239	21634	0.045	0.947	15.818	Sum In Feature 3	7.85	ECL deviates -0.004	16:1 w7c/16:1 w6c	
10.391	7083	0.042	0.947	15.908	16:1 w5c	2.57	ECL deviates -0.001		
10.543	39546	0.042	0.946	16.000	16:0	14.34	ECL deviates  0.000	Reference -0.003	
10.626	401	0.033	----	16.047		----			
11.081	80116	0.061	----	16.310		----			
11.290	38214	0.080	0.945	16.431	Sum In Feature 9	13.84	ECL deviates -0.001	16:0 10-methyl	
11.637	5770	0.040	0.945	16.631	17:0 iso	2.09	ECL deviates  0.001	Reference -0.002	
11.795	5713	0.044	0.945	16.722	17:0 anteiso	2.07	ECL deviates -0.001	Reference -0.003	
11.918	1690	0.039	0.945	16.793	17:1 w8c	0.61	ECL deviates  0.001		
12.084	5897	0.044	0.945	16.889	17:0 cyclo	2.14	ECL deviates  0.001		
12.276	1465	0.039	0.945	17.000	17:0	0.53	ECL deviates  0.000	Reference -0.003	
12.345	2997	0.040	0.945	17.039	16:1 2OH	1.09	ECL deviates -0.009		
12.452	298	0.036	----	17.100		----			
12.996	1714	0.044	0.945	17.408	17:0 10-methyl	0.62	ECL deviates -0.001		
13.146	770	0.037	----	17.494		----			
13.545	11280	0.048	0.946	17.720	Sum In Feature 5	4.09	ECL deviates  0.000	18:2 w6,9c/18:0 ante	
13.635	22124	0.055	0.946	17.771	18:1 w9c	8.02	ECL deviates  0.002		
13.725	23514	0.049	0.946	17.822	Sum In Feature 8	8.53	ECL deviates -0.001	18:1 w7c	
13.880	2947	0.055	0.946	17.910	18:1 w5c	1.07	ECL deviates -0.009		
14.036	8345	0.045	0.947	17.998	18:0	3.03	ECL deviates -0.002	Reference -0.005	
14.180	1826	0.039	0.947	18.081	18:1 w7c 11-methyl	0.66	ECL deviates  0.000		
14.610	14876	0.060	----	18.327		----			
14.724	8763	0.053	0.948	18.392	18:0 10-methyl, TBSA	3.18	ECL deviates  0.000		
14.781	5017	0.049	----	18.425		----			
15.345	1181	0.055	0.949	18.747	Sum In Feature 6	0.43	ECL deviates -0.009	19:1 w11c/19:1 w9c	
15.618	14934	0.048	0.949	18.904	19:0 cyclo w8c	5.43	ECL deviates  0.002		
16.474	1288	0.036	0.950	19.398	20:4 w6,9,12,15c	0.47	ECL deviates  0.003		
17.120	1485	0.052	0.951	19.771	20:1 w9c	0.54	ECL deviates  0.001		
17.517	1200	0.046	0.951	20.001	20:0	0.44	ECL deviates  0.001	Reference -0.004	
17.853	848	0.050	----	20.195		----	> max rt		
----	21634	---	----	----	Summed Feature 3	7.85	16:1 w7c/16:1 w6c	16:1 w6c/16:1 w7c	
----	11280	---	----	----	Summed Feature 5	4.09	18:2 w6,9c/18:0 ante	18:0 ante/18:2 w6,9c	
----	1181	---	----	----	Summed Feature 6	0.43	19:1 w11c/19:1 w9c	19:1 w9c/19:1 w11c	
----	23514	---	----	----	Summed Feature 8	8.53	18:1 w7c	18:1 w6c	
----	38214	---	----	----	Summed Feature 9	13.84	17:1 iso w9c	16:0 10-methyl	

ECL Deviation: 0.004                            Reference ECL Shift: 0.003      Number Reference Peaks: 13
Total Response: 388674                         Total Named: 275150
Percent Named: 70.79%                         Total Amount: 262834
Profile Comment:   Percent named is less than 85.00.

*** No Matches found in TSBA6
