# Supplementary material for: A green garlic (Allium sativum L.) based intercropping system reduces the strain of continuous monocropping in cucumber (Cucumis sativus L.) by adjusting the micro-ecological environment of soil
Source: PeerJ. 2019 Jul 15;7:e7267. doi: 10.7717/peerj.7267 (PMC6637937; doi:10.7717/peerj.7267)
Supplement: Data S1 [file peerj-07-7267-s001.zip › supplemental_Data_S1/15 days after interplanted/GR-1.rtf]

Volume: DATA            File: E131084.29A        Samp Ctr: 3                  ID Number: 1001 
Type: Samp                   Bottle: 2                        Method: TSBA6 
Created: 1/8/2013 11:05:05 AM 
Sample ID: 39 


RT	Response	Ar/Ht	RFact	ECL	Peak Name	Percent	Comment1	Comment2	
1.646	4.59E+8	0.029	----	7.014	SOLVENT PEAK	----	< min rt		
1.777	2967	0.022	----	7.271		----	< min rt		
1.923	151	0.021	----	7.558		----	< min rt		
3.059	567	0.027	----	9.787		----			
3.355	398	0.033	----	10.270		----			
4.406	700	0.025	----	11.586		----			
4.561	157	0.020	----	11.753		----			
4.907	1608	0.032	1.021	12.102	11:0 iso 3OH	0.76	ECL deviates  0.013		
5.111	3117	0.040	----	12.278		----			
5.497	374	0.031	1.003	12.610	13:0 iso	0.17	ECL deviates -0.004	Reference -0.011	
6.404	1077	0.034	----	13.331		----			
6.805	1287	0.035	0.976	13.622	14:0 iso	0.58	ECL deviates  0.003	Reference -0.003	
7.329	1585	0.034	0.969	14.002	14:0	0.71	ECL deviates  0.002	Reference -0.003	
7.775	7392	0.046	----	14.290		----			
8.006	713	0.036	0.962	14.440	15:1 iso G	0.32	ECL deviates  0.000		
8.292	11829	0.038	0.959	14.624	15:0 iso	5.24	ECL deviates  0.001	Reference -0.003	
8.432	7064	0.038	0.958	14.715	15:0 anteiso	3.13	ECL deviates  0.002	Reference -0.002	
8.815	1119	0.036	0.955	14.962	unknown 14.959	----	ECL deviates  0.003		
8.875	1764	0.037	0.955	15.001	15:0	----	ECL deviates  0.001		
8.958	688	0.041	----	15.051		----			
9.380	503	0.043	----	15.303		----			
9.619	1259	0.050	0.951	15.447	16:1 iso G	0.55	ECL deviates  0.005		
9.921	6370	0.041	0.949	15.627	16:0 iso	2.79	ECL deviates  0.000	Reference -0.003	
10.161	1751	0.040	0.949	15.771	16:1 w9c	0.77	ECL deviates -0.003		
10.239	17592	0.043	0.948	15.817	Sum In Feature 3	7.71	ECL deviates -0.005	16:1 w7c/16:1 w6c	
10.389	5473	0.040	0.948	15.907	16:1 w5c	2.40	ECL deviates -0.002		
10.543	36047	0.042	0.947	15.999	16:0	15.77	ECL deviates -0.001	Reference -0.004	
11.084	121430	0.059	----	16.312		----			
11.288	36954	0.082	0.946	16.430	Sum In Feature 9	16.15	ECL deviates -0.002	16:0 10-methyl	
11.425	13939	0.089	0.946	16.508	15:0 3OH	----	> max ar/ht		
11.634	10674	0.059	0.946	16.629	17:0 iso	4.66	ECL deviates -0.001	Reference -0.003	
11.795	9404	0.064	0.945	16.722	17:0 anteiso	4.11	ECL deviates -0.001	Reference -0.003	
11.915	4393	0.066	0.945	16.791	17:1 w8c	1.92	ECL deviates -0.001		
12.084	8146	0.061	0.945	16.889	17:0 cyclo	3.56	ECL deviates  0.001		
12.275	1875	0.040	0.945	16.999	17:0	0.82	ECL deviates -0.001	Reference -0.003	
12.344	2601	0.045	0.945	17.038	16:1 2OH	1.14	ECL deviates -0.010		
12.992	1613	0.040	0.945	17.406	17:0 10-methyl	0.70	ECL deviates -0.003		
13.151	959	0.049	----	17.496		----			
13.546	9121	0.047	0.946	17.720	Sum In Feature 5	3.98	ECL deviates  0.000	18:2 w6,9c/18:0 ante	
13.676	28403	0.059	----	17.794		----			
13.724	21262	0.045	0.946	17.821	Sum In Feature 8	9.29	ECL deviates -0.002	18:1 w7c	
13.877	2431	0.050	0.946	17.908	18:1 w5c	1.06	ECL deviates -0.011		
14.036	7620	0.048	0.946	17.998	18:0	3.33	ECL deviates -0.002	Reference -0.004	
14.178	1370	0.040	0.946	18.080	18:1 w7c 11-methyl	0.60	ECL deviates -0.001		
14.606	34738	0.063	----	18.324		----			
14.723	20790	0.092	0.947	18.391	18:0 10-methyl, TBSA	----	> max ar/ht		
15.283	1427	0.078	----	18.711		----			
15.620	12455	0.046	0.948	18.903	19:0 cyclo w8c	5.45	ECL deviates  0.001		
16.476	2313	0.046	0.949	19.397	20:4 w6,9,12,15c	1.01	ECL deviates  0.002		
16.626	1670	0.048	----	19.484		----			
17.009	1851	0.046	----	19.705		----			
17.115	1581	0.052	0.949	19.767	20:1 w9c	0.69	ECL deviates -0.003		
17.521	1473	0.063	0.950	20.002	20:0	0.65	ECL deviates  0.002	Reference -0.001	
17.840	673	0.035	----	20.186		----	> max rt		
18.485	1192	0.042	----	20.559		----	> max rt		
----	17592	---	----	----	Summed Feature 3	7.71	16:1 w7c/16:1 w6c	16:1 w6c/16:1 w7c	
----	9121	---	----	----	Summed Feature 5	3.98	18:2 w6,9c/18:0 ante	18:0 ante/18:2 w6,9c	
----	21262	---	----	----	Summed Feature 8	9.29	18:1 w7c	18:1 w6c	
----	36954	---	----	----	Summed Feature 9	16.15	17:1 iso w9c	16:0 10-methyl	

ECL Deviation: 0.004                            Reference ECL Shift: 0.004      Number Reference Peaks: 12
Total Response: 468043                         Total Named: 228236
Percent Named: 48.76%                         Total Amount: 252138
Profile Comment:   Percent named is less than 85.00.

*** No Matches found in TSBA6
