# Supplementary material for: A green garlic (Allium sativum L.) based intercropping system reduces the strain of continuous monocropping in cucumber (Cucumis sativus L.) by adjusting the micro-ecological environment of soil
Source: PeerJ. 2019 Jul 15;7:e7267. doi: 10.7717/peerj.7267 (PMC6637937; doi:10.7717/peerj.7267)
Supplement: Data S1 [file peerj-07-7267-s001.zip › supplemental_Data_S1/15 days after interplanted/GR-2.rtf]

Volume: DATA            File: E131084.29A        Samp Ctr: 4                  ID Number: 1002 
Type: Samp                   Bottle: 3                        Method: TSBA6 
Created: 1/8/2013 11:29:19 AM 
Sample ID: 40 


RT	Response	Ar/Ht	RFact	ECL	Peak Name	Percent	Comment1	Comment2	
1.645	4.595E+8	0.029	----	7.004	SOLVENT PEAK	----	< min rt		
1.777	1076	0.013	----	7.262		----	< min rt		
2.284	291	0.021	----	8.259		----	< min rt		
3.060	421	0.033	----	9.782		----			
4.407	450	0.030	----	11.578		----			
4.907	1137	0.032	1.021	12.096	11:0 iso 3OH	0.66	ECL deviates  0.007		
5.005	283	0.031	----	12.180		----			
6.404	521	0.033	----	13.326		----			
6.807	1073	0.038	0.976	13.620	14:0 iso	0.59	ECL deviates  0.001	Reference -0.001	
7.328	1355	0.037	0.969	13.999	14:0	0.74	ECL deviates -0.001	Reference -0.003	
7.810	986	0.039	----	14.311		----			
8.010	693	0.034	0.962	14.440	15:1 iso G	0.38	ECL deviates  0.000		
8.293	10563	0.038	0.959	14.623	15:0 iso	5.73	ECL deviates  0.000	Reference -0.002	
8.433	6418	0.041	0.958	14.714	15:0 anteiso	3.48	ECL deviates  0.001	Reference -0.002	
8.876	1543	0.038	0.955	15.000	15:0	----	ECL deviates  0.000		
9.618	1469	0.066	0.951	15.445	16:1 iso G	0.79	ECL deviates  0.003		
9.922	6596	0.042	0.949	15.627	16:0 iso	3.54	ECL deviates  0.000	Reference -0.002	
10.156	1638	0.044	0.949	15.767	16:1 w9c	0.88	ECL deviates -0.007		
10.239	15787	0.041	0.948	15.817	Sum In Feature 3	8.47	ECL deviates -0.005	16:1 w7c/16:1 w6c	
10.392	4971	0.042	0.948	15.909	16:1 w5c	2.67	ECL deviates  0.000		
10.543	34162	0.042	0.947	15.999	16:0	18.32	ECL deviates -0.001	Reference -0.003	
11.288	19389	0.070	0.946	16.430	Sum In Feature 9	10.38	ECL deviates -0.002	16:0 10-methyl	
11.441	2813	0.058	0.946	16.518	17:1 anteiso w9c	1.51	ECL deviates -0.006		
11.635	5632	0.049	0.946	16.630	17:0 iso	3.01	ECL deviates  0.000	Reference -0.003	
11.797	4923	0.043	0.945	16.724	17:0 anteiso	2.63	ECL deviates  0.001	Reference -0.002	
11.917	1700	0.044	0.945	16.793	17:1 w8c	0.91	ECL deviates  0.001		
12.083	4731	0.048	0.945	16.889	17:0 cyclo	2.53	ECL deviates  0.001		
12.277	1062	0.039	0.945	17.001	17:0	0.57	ECL deviates  0.001	Reference -0.002	
12.345	2111	0.038	0.945	17.040	16:1 2OH	1.13	ECL deviates -0.008		
12.996	1543	0.049	0.945	17.409	17:0 10-methyl	0.83	ECL deviates  0.000		
13.152	649	0.043	----	17.498		----			
13.546	9453	0.047	0.946	17.722	Sum In Feature 5	5.06	ECL deviates  0.002	18:2 w6,9c/18:0 ante	
13.676	26563	0.061	----	17.795		----			
13.722	17639	0.044	0.946	17.822	Sum In Feature 8	9.44	ECL deviates -0.001	18:1 w7c	
13.870	2858	0.055	----	17.905		----			
14.035	7237	0.049	0.946	17.999	18:0	3.87	ECL deviates -0.001	Reference -0.004	
14.178	1279	0.038	0.946	18.081	18:1 w7c 11-methyl	0.68	ECL deviates  0.000		
14.725	3933	0.050	0.947	18.394	18:0 10-methyl, TBSA	2.11	ECL deviates  0.002		
14.785	2160	0.044	----	18.427		----			
15.026	602	0.050	----	18.565		----			
15.343	945	0.042	0.948	18.746	Sum In Feature 6	0.51	ECL deviates -0.010	19:1 w11c/19:1 w9c	
15.617	12165	0.050	0.948	18.903	19:0 cyclo w8c	6.53	ECL deviates  0.001		
15.896	311817	0.150	----	19.063		----	> max ar/ht		
16.476	2143	0.049	0.949	19.398	20:4 w6,9,12,15c	1.15	ECL deviates  0.003		
16.620	898	0.054	----	19.482		----			
17.121	689	0.034	0.949	19.772	20:1 w9c	0.37	ECL deviates  0.002		
17.516	1004	0.042	0.950	20.000	20:0	0.54	ECL deviates  0.000	Reference -0.004	
17.846	745	0.039	----	20.191		----	> max rt		
18.486	3377	0.075	----	20.561		----	> max rt		
----	15787	---	----	----	Summed Feature 3	8.47	16:1 w7c/16:1 w6c	16:1 w6c/16:1 w7c	
----	9453	---	----	----	Summed Feature 5	5.06	18:2 w6,9c/18:0 ante	18:0 ante/18:2 w6,9c	
----	945	---	----	----	Summed Feature 6	0.51	19:1 w11c/19:1 w9c	19:1 w9c/19:1 w11c	
----	17639	---	----	----	Summed Feature 8	9.44	18:1 w7c	18:1 w6c	
----	19389	---	----	----	Summed Feature 9	10.38	17:1 iso w9c	16:0 10-methyl	

ECL Deviation: 0.003                            Reference ECL Shift: 0.003      Number Reference Peaks: 11
Total Response: 534463                         Total Named: 186254
Percent Named: 34.85%                         Total Amount: 178187
Profile Comment:   Percent named is less than 85.00.

*** Library match not attempted
