# Supplementary material for: A green garlic (Allium sativum L.) based intercropping system reduces the strain of continuous monocropping in cucumber (Cucumis sativus L.) by adjusting the micro-ecological environment of soil
Source: PeerJ. 2019 Jul 15;7:e7267. doi: 10.7717/peerj.7267 (PMC6637937; doi:10.7717/peerj.7267)
Supplement: Data S1 [file peerj-07-7267-s001.zip › supplemental_Data_S1/15 days after interplanted/GR-3.rtf]

Volume: DATA            File: E131084.29A        Samp Ctr: 5                  ID Number: 1003 
Type: Samp                   Bottle: 4                        Method: TSBA6 
Created: 1/8/2013 11:53:36 AM 
Sample ID: 41 


RT	Response	Ar/Ht	RFact	ECL	Peak Name	Percent	Comment1	Comment2	
1.645	4.584E+8	0.029	----	7.007	SOLVENT PEAK	----	< min rt		
1.777	3013	0.025	----	7.265		----	< min rt		
2.281	171	0.018	----	8.256		----	< min rt		
4.407	294	0.023	----	11.576		----			
4.908	1087	0.030	1.021	12.094	11:0 iso 3OH	0.59	ECL deviates  0.005		
5.511	641	0.046	1.003	12.616	13:0 iso	0.34	ECL deviates  0.002	Reference  0.001	
6.806	1193	0.038	0.976	13.618	14:0 iso	0.62	ECL deviates -0.001	Reference -0.002	
7.329	1372	0.037	0.969	13.999	14:0	0.71	ECL deviates -0.001	Reference -0.003	
7.803	1771	0.044	----	14.306		----			
8.006	833	0.041	0.962	14.437	15:1 iso G	0.43	ECL deviates -0.003		
8.293	10055	0.037	0.959	14.623	15:0 iso	5.12	ECL deviates  0.000	Reference -0.002	
8.433	6076	0.039	0.958	14.714	15:0 anteiso	3.09	ECL deviates  0.001	Reference -0.002	
8.629	489	0.039	----	14.841		----			
8.877	1455	0.035	0.955	15.001	15:0	----	ECL deviates  0.001		
8.964	496	0.033	----	15.053		----			
9.605	1517	0.056	0.951	15.437	16:1 iso G	0.77	ECL deviates -0.005		
9.921	5841	0.040	0.949	15.627	16:0 iso	2.95	ECL deviates  0.000	Reference -0.003	
10.160	1748	0.039	0.949	15.770	16:1 w9c	0.88	ECL deviates -0.004		
10.238	16401	0.042	0.948	15.817	Sum In Feature 3	8.26	ECL deviates -0.005	16:1 w7c/16:1 w6c	
10.391	5203	0.041	0.948	15.908	16:1 w5c	2.62	ECL deviates -0.001		
10.543	32391	0.040	0.947	15.999	16:0	16.31	ECL deviates -0.001	Reference -0.003	
11.093	16766	0.076	----	16.317		----			
11.289	19288	0.062	0.946	16.430	Sum In Feature 9	9.70	ECL deviates -0.002	16:0 10-methyl	
11.636	4360	0.045	0.946	16.630	17:0 iso	2.19	ECL deviates  0.000	Reference -0.002	
11.796	4372	0.044	0.945	16.723	17:0 anteiso	2.20	ECL deviates  0.000	Reference -0.003	
11.917	1500	0.042	0.945	16.792	17:1 w8c	0.75	ECL deviates  0.000		
12.084	5124	0.049	0.945	16.889	17:0 cyclo	2.57	ECL deviates  0.001		
12.275	1642	0.045	0.945	16.999	17:0	0.82	ECL deviates -0.001	Reference -0.003	
12.345	2498	0.046	0.945	17.039	16:1 2OH	1.25	ECL deviates -0.009		
12.994	1313	0.042	0.945	17.407	17:0 10-methyl	0.66	ECL deviates -0.002		
13.547	8438	0.045	0.946	17.721	Sum In Feature 5	4.24	ECL deviates  0.001	18:2 w6,9c/18:0 ante	
13.638	11069	0.038	0.946	17.772	18:1 w9c	5.56	ECL deviates  0.003		
13.676	13644	0.036	----	17.794		----			
13.724	18785	0.042	0.946	17.821	Sum In Feature 8	9.44	ECL deviates -0.002	18:1 w7c	
13.878	2125	0.051	0.946	17.908	18:1 w5c	1.07	ECL deviates -0.011		
14.035	6595	0.048	0.946	17.998	18:0	3.32	ECL deviates -0.002	Reference -0.004	
14.179	1454	0.039	0.946	18.080	18:1 w7c 11-methyl	0.73	ECL deviates -0.001		
14.629	2400	0.051	----	18.337		----			
14.726	8172	0.079	0.947	18.392	18:0 10-methyl, TBSA	4.11	ECL deviates  0.000		
15.336	679	0.034	0.948	18.740	19:0 anteiso	0.34	ECL deviates  0.009	Reference  0.008	
15.619	13104	0.050	0.948	18.901	19:0 cyclo w8c	6.60	ECL deviates -0.001		
15.900	322669	0.148	----	19.063		----	> max ar/ht		
16.475	1637	0.042	0.949	19.394	20:4 w6,9,12,15c	0.83	ECL deviates -0.001		
17.112	813	0.041	0.949	19.762	20:1 w9c	0.41	ECL deviates -0.008		
17.517	1025	0.049	0.950	19.996	20:0	0.52	ECL deviates -0.004	Reference -0.004	
17.847	740	0.045	----	20.186		----	> max rt		
18.482	1885	0.039	----	20.553		----	> max rt		
----	16401	---	----	----	Summed Feature 3	8.26	16:1 w7c/16:1 w6c	16:1 w6c/16:1 w7c	
----	8438	---	----	----	Summed Feature 5	4.24	18:2 w6,9c/18:0 ante	18:0 ante/18:2 w6,9c	
----	18785	---	----	----	Summed Feature 8	9.44	18:1 w7c	18:1 w6c	
----	19288	---	----	----	Summed Feature 9	9.70	17:1 iso w9c	16:0 10-methyl	

ECL Deviation: 0.004                            Reference ECL Shift: 0.003      Number Reference Peaks: 13
Total Response: 556881                         Total Named: 198351
Percent Named: 35.62%                         Total Amount: 189572
Profile Comment:   Percent named is less than 85.00.

*** Library match not attempted
