# Supplementary material for: A green garlic (Allium sativum L.) based intercropping system reduces the strain of continuous monocropping in cucumber (Cucumis sativus L.) by adjusting the micro-ecological environment of soil
Source: PeerJ. 2019 Jul 15;7:e7267. doi: 10.7717/peerj.7267 (PMC6637937; doi:10.7717/peerj.7267)
Supplement: Data S1 [file peerj-07-7267-s001.zip › supplemental_Data_S1/30 days after interplanted/CB-1.rtf]

Volume: DATA            File: E131084.29A        Samp Ctr: 26                ID Number: 1011 
Type: Samp                   Bottle: 6                        Method: TSBA6 
Created: 1/8/2013 8:23:05 PM 
Sample ID: 60 


RT	Response	Ar/Ht	RFact	ECL	Peak Name	Percent	Comment1	Comment2	
1.646	4.504E+8	0.028	----	7.007	SOLVENT PEAK	----	< min rt		
1.777	2410	0.022	----	7.265		----	< min rt		
2.796	296	0.033	----	9.266		----			
4.909	748	0.033	1.021	12.099	11:0 iso 3OH	0.31	ECL deviates  0.010		
5.121	1157	0.031	----	12.282		----			
5.505	302	0.030	1.002	12.614	13:0 iso	0.12	ECL deviates  0.000	Reference -0.005	
6.399	346	0.029	----	13.325		----			
6.806	1586	0.038	0.975	13.620	14:0 iso	0.62	ECL deviates  0.001	Reference -0.002	
7.330	1903	0.038	0.967	14.000	14:0	0.74	ECL deviates  0.000	Reference -0.003	
7.792	4252	0.053	----	14.300		----			
8.008	806	0.039	0.960	14.439	15:1 iso G	0.31	ECL deviates -0.001		
8.293	14865	0.037	0.958	14.623	15:0 iso	5.69	ECL deviates  0.000	Reference -0.002	
8.432	8595	0.039	0.957	14.713	15:0 anteiso	3.29	ECL deviates  0.000	Reference -0.002	
8.877	1480	0.031	0.953	15.001	15:0	----	ECL deviates  0.001		
8.968	646	0.036	----	15.056		----			
9.620	2083	0.056	0.949	15.446	16:1 iso G	0.79	ECL deviates  0.004		
9.922	7849	0.040	0.948	15.627	16:0 iso	2.98	ECL deviates  0.000	Reference -0.002	
10.158	2606	0.049	0.947	15.768	16:1 w9c	0.99	ECL deviates -0.006		
10.240	23320	0.043	0.947	15.817	Sum In Feature 3	8.83	ECL deviates -0.005	16:1 w7c/16:1 w6c	
10.391	6544	0.040	0.947	15.907	16:1 w5c	2.48	ECL deviates -0.002		
10.542	34436	0.040	0.946	15.998	16:0	13.03	ECL deviates -0.002	Reference -0.004	
10.631	721	0.043	----	16.049		----			
11.080	44674	0.066	----	16.308		----			
11.288	38241	0.079	0.945	16.428	Sum In Feature 9	14.45	ECL deviates -0.004	16:0 10-methyl	
11.453	6981	0.074	0.945	16.523	17:1 anteiso w9c	2.64	ECL deviates -0.001		
11.635	9219	0.054	0.944	16.628	17:0 iso	3.48	ECL deviates -0.002	Reference -0.003	
11.796	9123	0.053	0.944	16.721	17:0 anteiso	3.45	ECL deviates -0.002	Reference -0.003	
11.918	2913	0.050	0.944	16.792	17:1 w8c	1.10	ECL deviates  0.000		
12.083	8678	0.056	0.944	16.887	17:0 cyclo	3.28	ECL deviates -0.001		
12.279	1622	0.043	0.944	17.001	17:0	0.61	ECL deviates  0.001	Reference -0.001	
12.344	3544	0.043	----	17.037		----			
12.996	1951	0.049	0.944	17.407	17:0 10-methyl	0.74	ECL deviates -0.002		
13.147	952	0.045	----	17.493		----			
13.546	5021	0.046	0.945	17.720	Sum In Feature 5	1.90	ECL deviates  0.000	18:2 w6,9c/18:0 ante	
13.632	18357	0.051	0.945	17.768	18:1 w9c	6.94	ECL deviates -0.001		
13.723	22719	0.048	0.945	17.820	Sum In Feature 8	8.59	ECL deviates -0.003	18:1 w7c	
13.875	3220	0.050	----	17.906		----			
14.035	8127	0.047	0.945	17.997	18:0	3.07	ECL deviates -0.003	Reference -0.005	
14.179	2021	0.050	0.945	18.079	18:1 w7c 11-methyl	0.76	ECL deviates -0.002		
14.606	7794	0.060	----	18.323		----			
14.723	12621	0.084	0.946	18.390	18:0 10-methyl, TBSA	----	> max ar/ht		
15.338	841	0.036	----	18.742		----		Reference  0.008	
15.617	18405	0.051	0.947	18.902	19:0 cyclo w8c	6.97	ECL deviates  0.000		
15.891	289767	0.151	----	19.059		----	> max ar/ht		
16.475	1897	0.048	0.947	19.397	20:4 w6,9,12,15c	0.72	ECL deviates  0.002		
16.609	850	0.045	----	19.474		----			
17.120	1844	0.051	0.948	19.770	20:1 w9c	0.70	ECL deviates  0.000		
17.510	1134	0.042	0.948	19.995	20:0	0.43	ECL deviates -0.005	Reference -0.009	
17.847	1099	0.047	----	20.190		----	> max rt		
----	23320	---	----	----	Summed Feature 3	8.83	16:1 w7c/16:1 w6c	16:1 w6c/16:1 w7c	
----	5021	---	----	----	Summed Feature 5	1.90	18:2 w6,9c/18:0 ante	18:0 ante/18:2 w6,9c	
----	22719	---	----	----	Summed Feature 8	8.59	18:1 w7c	18:1 w6c	
----	38241	---	----	----	Summed Feature 9	14.45	17:1 iso w9c	16:0 10-methyl	

ECL Deviation: 0.003                            Reference ECL Shift: 0.004      Number Reference Peaks: 13
Total Response: 635574                         Total Named: 263892
Percent Named: 41.52%                         Total Amount: 263325
Profile Comment:   Percent named is less than 85.00.

*** Library match not attempted
