# Supplementary material for: A green garlic (Allium sativum L.) based intercropping system reduces the strain of continuous monocropping in cucumber (Cucumis sativus L.) by adjusting the micro-ecological environment of soil
Source: PeerJ. 2019 Jul 15;7:e7267. doi: 10.7717/peerj.7267 (PMC6637937; doi:10.7717/peerj.7267)
Supplement: Data S1 [file peerj-07-7267-s001.zip › supplemental_Data_S1/30 days after interplanted/CB-2.rtf]

Volume: DATA            File: E131094.42A        Samp Ctr: 4                  ID Number: 1002 
Type: Samp                   Bottle: 3                        Method: TSBA6 
Created: 1/9/2013 11:47:33 AM 
Sample ID: 61 


RT	Response	Ar/Ht	RFact	ECL	Peak Name	Percent	Comment1	Comment2	
1.645	4.547E+8	0.029	----	7.007	SOLVENT PEAK	----	< min rt		
1.777	3277	0.023	----	7.266		----	< min rt		
2.284	249	0.026	----	8.262		----	< min rt		
3.058	335	0.021	----	9.785		----			
3.349	306	0.027	----	10.260		----			
4.434	604	0.040	1.037	11.611	12:0 iso	0.21	ECL deviates  0.002	Reference -0.002	
4.907	1019	0.034	1.017	12.097	11:0 iso 3OH	0.35	ECL deviates  0.008		
5.110	3568	0.034	----	12.273		----			
5.504	275	0.033	0.998	12.612	13:0 iso	0.09	ECL deviates -0.002	Reference -0.005	
6.806	1605	0.037	0.972	13.619	14:0 iso	0.53	ECL deviates  0.000	Reference -0.002	
7.328	2064	0.035	0.965	13.998	14:0	0.68	ECL deviates -0.002	Reference -0.004	
7.776	10095	0.049	----	14.289		----			
8.004	768	0.030	0.958	14.437	15:1 iso G	0.25	ECL deviates -0.003		
8.292	16452	0.038	0.956	14.623	15:0 iso	5.38	ECL deviates  0.000	Reference -0.002	
8.433	9521	0.040	0.955	14.714	15:0 anteiso	3.11	ECL deviates  0.001	Reference -0.001	
8.875	1700	0.037	0.952	15.000	15:0	----	ECL deviates  0.000		
8.964	845	0.035	----	15.054		----			
9.614	1704	0.051	0.949	15.443	16:1 iso G	0.55	ECL deviates  0.001		
9.920	8491	0.041	0.948	15.626	16:0 iso	2.76	ECL deviates -0.001	Reference -0.003	
10.159	3094	0.053	0.948	15.769	16:1 w9c	1.00	ECL deviates -0.005		
10.237	27363	0.044	0.947	15.816	Sum In Feature 3	8.87	ECL deviates -0.006	16:1 w7c/16:1 w6c	
10.389	7798	0.044	0.947	15.907	16:1 w5c	2.53	ECL deviates -0.002		
10.542	39540	0.042	0.947	15.999	16:0	12.81	ECL deviates -0.001	Reference -0.003	
11.086	135858	0.058	----	16.313		----			
11.288	51729	0.083	0.946	16.430	Sum In Feature 9	16.75	ECL deviates -0.002	16:0 10-methyl	
11.633	6448	0.041	0.946	16.629	17:0 iso	2.09	ECL deviates -0.001	Reference -0.003	
11.795	6920	0.044	0.946	16.723	17:0 anteiso	2.24	ECL deviates  0.000	Reference -0.002	
11.915	1780	0.045	0.946	16.792	17:1 w8c	0.58	ECL deviates  0.000		
12.083	7012	0.047	0.946	16.889	17:0 cyclo	2.27	ECL deviates  0.001		
12.275	1537	0.042	0.946	17.000	17:0	0.50	ECL deviates  0.000	Reference -0.002	
12.343	3590	0.042	0.946	17.039	16:1 2OH	1.16	ECL deviates -0.009		
12.466	106	0.014	----	17.109		----	< min ar/ht		
12.994	2038	0.044	0.947	17.407	17:0 10-methyl	0.66	ECL deviates -0.002		
13.148	873	0.037	----	17.495		----			
13.546	6215	0.046	0.948	17.720	Sum In Feature 5	2.02	ECL deviates  0.000	18:2 w6,9c/18:0 ante	
13.631	20516	0.052	0.948	17.769	18:1 w9c	6.65	ECL deviates  0.000		
13.724	25780	0.050	0.948	17.822	Sum In Feature 8	8.36	ECL deviates -0.001	18:1 w7c	
13.878	3702	0.059	0.948	17.909	18:1 w5c	1.20	ECL deviates -0.010		
14.034	9391	0.045	0.948	17.997	18:0	3.05	ECL deviates -0.003	Reference -0.005	
14.179	2307	0.046	0.949	18.080	18:1 w7c 11-methyl	0.75	ECL deviates -0.001		
14.604	34469	0.063	----	18.324		----			
14.723	13877	0.059	0.949	18.391	18:0 10-methyl, TBSA	4.51	ECL deviates -0.001		
14.784	6885	0.043	----	18.426		----			
15.335	1299	0.044	----	18.742		----		Reference  0.008	
15.433	392	0.036	----	18.798		----			
15.619	20488	0.051	0.951	18.905	19:0 cyclo w8c	6.67	ECL deviates  0.003		
15.864	220941	0.153	----	19.045		----	> max ar/ht		
16.473	1694	0.043	0.952	19.397	20:4 w6,9,12,15c	0.55	ECL deviates  0.002		
16.605	533	0.033	----	19.473		----			
17.120	1306	0.043	0.952	19.771	20:1 w9c	0.43	ECL deviates  0.001		
17.506	1284	0.045	0.952	19.994	20:0	0.42	ECL deviates -0.006	Reference -0.010	
17.846	1071	0.043	----	20.191		----	> max rt		
18.180	1120	0.065	----	20.384		----	> max rt		
18.479	953	0.035	----	20.557		----	> max rt		
----	27363	---	----	----	Summed Feature 3	8.87	16:1 w7c/16:1 w6c	16:1 w6c/16:1 w7c	
----	6215	---	----	----	Summed Feature 5	2.02	18:2 w6,9c/18:0 ante	18:0 ante/18:2 w6,9c	
----	25780	---	----	----	Summed Feature 8	8.36	18:1 w7c	18:1 w6c	
----	51729	---	----	----	Summed Feature 9	16.75	17:1 iso w9c	16:0 10-methyl	

ECL Deviation: 0.003                            Reference ECL Shift: 0.004      Number Reference Peaks: 14
Total Response: 724416                         Total Named: 307910
Percent Named: 42.50%                         Total Amount: 293804
Profile Comment:   Percent named is less than 85.00.

*** No Matches found in TSBA6
