# Supplementary material for: A green garlic (Allium sativum L.) based intercropping system reduces the strain of continuous monocropping in cucumber (Cucumis sativus L.) by adjusting the micro-ecological environment of soil
Source: PeerJ. 2019 Jul 15;7:e7267. doi: 10.7717/peerj.7267 (PMC6637937; doi:10.7717/peerj.7267)
Supplement: Data S1 [file peerj-07-7267-s001.zip › supplemental_Data_S1/30 days after interplanted/CR-1.rtf]

Volume: DATA            File: E131094.42A        Samp Ctr: 3                  ID Number: 1001 
Type: Samp                   Bottle: 2                        Method: TSBA6 
Created: 1/9/2013 11:23:26 AM 
Sample ID: 45 


RT	Response	Ar/Ht	RFact	ECL	Peak Name	Percent	Comment1	Comment2	
1.645	4.594E+8	0.029	----	7.014	SOLVENT PEAK	----	< min rt		
1.777	2049	0.015	----	7.273		----	< min rt		
3.059	229	0.025	----	9.789		----			
3.353	366	0.025	----	10.277		----			
4.569	236	0.027	----	11.763		----			
4.786	425	0.037	1.020	11.997	12:0	0.15	ECL deviates -0.003	Reference -0.013	
4.906	1735	0.032	1.017	12.103	11:0 iso 3OH	0.63	ECL deviates  0.014		
5.011	443	0.033	----	12.192		----			
5.112	2731	0.036	----	12.280		----			
5.503	350	0.032	0.998	12.616	13:0 iso	0.12	ECL deviates  0.002	Reference -0.005	
6.398	243	0.027	----	13.327		----			
6.804	1405	0.035	0.972	13.621	14:0 iso	0.49	ECL deviates  0.002	Reference -0.003	
7.198	588	0.036	0.967	13.906	14:1 w5c	0.20	ECL deviates  0.005		
7.328	2228	0.040	0.965	14.001	14:0	0.77	ECL deviates  0.001	Reference -0.003	
7.778	7920	0.051	----	14.292		----			
8.007	901	0.041	0.958	14.440	15:1 iso G	0.31	ECL deviates  0.000		
8.292	13945	0.038	0.956	14.624	15:0 iso	4.76	ECL deviates  0.001	Reference -0.002	
8.431	8160	0.039	0.955	14.714	15:0 anteiso	2.79	ECL deviates  0.001	Reference -0.002	
8.875	1875	0.038	0.952	15.001	15:0	----	ECL deviates  0.001		
8.962	491	0.032	----	15.053		----			
9.615	1847	0.064	0.949	15.444	16:1 iso G	0.63	ECL deviates  0.002		
9.920	7391	0.042	0.948	15.626	16:0 iso	2.50	ECL deviates -0.001	Reference -0.003	
10.153	2762	0.046	0.948	15.766	16:1 w9c	0.94	ECL deviates -0.008		
10.238	32486	0.042	0.947	15.816	Sum In Feature 3	11.00	ECL deviates -0.006	16:1 w7c/16:1 w6c	
10.389	7226	0.044	0.947	15.907	16:1 w5c	2.45	ECL deviates -0.002		
10.542	38710	0.043	0.947	15.998	16:0	13.10	ECL deviates -0.002	Reference -0.003	
10.934	451	0.057	0.946	16.225	15:0 2OH	0.15	ECL deviates  0.006		
11.080	106326	0.058	----	16.309		----			
11.287	44592	0.090	0.946	16.429	Sum In Feature 9	15.08	ECL deviates -0.003	16:0 10-methyl	
11.435	12515	0.089	0.946	16.514	17:1 anteiso w9c	----	> max ar/ht		
11.633	11224	0.058	0.946	16.628	17:0 iso	3.79	ECL deviates -0.002	Reference -0.003	
11.794	10051	0.060	0.946	16.722	17:0 anteiso	3.40	ECL deviates -0.001	Reference -0.003	
11.915	4636	0.064	0.946	16.791	17:1 w8c	1.57	ECL deviates -0.001		
12.083	9909	0.063	0.946	16.889	17:0 cyclo	3.35	ECL deviates  0.001		
12.272	2118	0.046	0.946	16.998	17:0	0.72	ECL deviates -0.002	Reference -0.003	
12.343	3308	0.045	0.946	17.038	16:1 2OH	1.12	ECL deviates -0.010		
12.990	1872	0.046	0.947	17.405	17:0 10-methyl	0.63	ECL deviates -0.004		
13.143	1082	0.050	----	17.492		----			
13.544	4888	0.043	0.948	17.719	Sum In Feature 5	1.66	ECL deviates -0.001	18:2 w6,9c/18:0 ante	
13.632	18809	0.054	0.948	17.768	18:1 w9c	6.37	ECL deviates -0.001		
13.723	31771	0.050	0.948	17.820	Sum In Feature 8	10.76	ECL deviates -0.003	18:1 w7c	
13.873	3182	0.054	----	17.906		----			
14.033	7614	0.044	0.948	17.996	18:0	2.58	ECL deviates -0.004	Reference -0.006	
14.177	2499	0.046	0.949	18.078	18:1 w7c 11-methyl	0.85	ECL deviates -0.003		
14.599	23329	0.064	----	18.320		----			
14.720	16666	0.089	0.949	18.389	18:0 10-methyl, TBSA	----	> max ar/ht		
15.338	1131	0.048	----	18.743		----		Reference  0.009	
15.617	15305	0.048	0.951	18.903	19:0 cyclo w8c	5.20	ECL deviates  0.001		
16.473	2363	0.043	0.952	19.398	20:4 w6,9,12,15c	0.80	ECL deviates  0.003		
16.603	928	0.044	----	19.473		----			
17.118	2136	0.061	0.952	19.771	20:1 w9c	0.73	ECL deviates  0.001		
17.506	1216	0.043	0.952	19.996	20:0	0.41	ECL deviates -0.004	Reference -0.010	
17.849	1094	0.043	----	20.194		----	> max rt		
18.491	1419	0.046	----	20.566		----	> max rt		
----	32486	---	----	----	Summed Feature 3	11.00	16:1 w7c/16:1 w6c	16:1 w6c/16:1 w7c	
----	4888	---	----	----	Summed Feature 5	1.66	18:2 w6,9c/18:0 ante	18:0 ante/18:2 w6,9c	
----	31771	---	----	----	Summed Feature 8	10.76	18:1 w7c	18:1 w6c	
----	44592	---	----	----	Summed Feature 9	15.08	17:1 iso w9c	16:0 10-methyl	

ECL Deviation: 0.004                            Reference ECL Shift: 0.006      Number Reference Peaks: 14
Total Response: 472738                         Total Named: 294921
Percent Named: 62.39%                         Total Amount: 309286
Profile Comment:   Percent named is less than 85.00.

*** No Matches found in TSBA6
