# Supplementary material for: A green garlic (Allium sativum L.) based intercropping system reduces the strain of continuous monocropping in cucumber (Cucumis sativus L.) by adjusting the micro-ecological environment of soil
Source: PeerJ. 2019 Jul 15;7:e7267. doi: 10.7717/peerj.7267 (PMC6637937; doi:10.7717/peerj.7267)
Supplement: Data S1 [file peerj-07-7267-s001.zip › supplemental_Data_S1/30 days after interplanted/CR-2.rtf]

Volume: DATA            File: E131084.29A        Samp Ctr: 8                  ID Number: 1001 
Type: Samp                   Bottle: 2                        Method: TSBA6 
Created: 1/8/2013 1:06:22 PM 
Sample ID: 46 


RT	Response	Ar/Ht	RFact	ECL	Peak Name	Percent	Comment1	Comment2	
1.646	4.575E+8	0.029	----	7.006	SOLVENT PEAK	----	< min rt		
1.778	3475	0.024	----	7.266		----	< min rt		
2.285	332	0.023	----	8.261		----	< min rt		
4.907	1449	0.032	1.021	12.100	11:0 iso 3OH	0.51	ECL deviates  0.011		
5.120	1807	0.039	----	12.282		----			
5.503	285	0.030	1.003	12.612	13:0 iso	0.10	ECL deviates -0.002	Reference -0.006	
6.402	353	0.030	----	13.327		----			
6.806	1531	0.035	0.976	13.620	14:0 iso	0.52	ECL deviates  0.001	Reference -0.002	
7.329	1899	0.034	0.969	14.000	14:0	0.64	ECL deviates  0.000	Reference -0.003	
7.787	4919	0.054	----	14.296		----			
8.010	1027	0.038	0.962	14.440	15:1 iso G	0.34	ECL deviates  0.000		
8.294	15170	0.038	0.959	14.624	15:0 iso	5.04	ECL deviates  0.001	Reference -0.001	
8.434	8741	0.037	0.958	14.715	15:0 anteiso	2.90	ECL deviates  0.002	Reference -0.001	
8.630	509	0.038	----	14.841		----			
8.877	1930	0.039	0.955	15.001	15:0	----	ECL deviates  0.001		
8.968	824	0.036	----	15.056		----			
9.625	1767	0.053	0.951	15.449	16:1 iso G	0.58	ECL deviates  0.007		
9.921	7921	0.040	0.949	15.627	16:0 iso	2.60	ECL deviates  0.000	Reference -0.003	
10.158	3099	0.047	0.949	15.768	16:1 w9c	1.02	ECL deviates -0.006		
10.241	34091	0.044	0.948	15.818	Sum In Feature 3	11.19	ECL deviates -0.004	16:1 w7c/16:1 w6c	
10.391	7509	0.044	0.948	15.908	16:1 w5c	2.46	ECL deviates -0.001		
10.543	41808	0.042	0.947	15.999	16:0	13.72	ECL deviates -0.001	Reference -0.003	
11.081	55865	0.066	----	16.310		----			
11.289	37965	0.076	0.946	16.430	Sum In Feature 9	12.44	ECL deviates -0.002	16:0 10-methyl	
11.452	8765	0.091	0.946	16.524	17:1 anteiso w9c	----	> max ar/ht		
11.636	9229	0.052	0.946	16.630	17:0 iso	3.02	ECL deviates  0.000	Reference -0.002	
11.796	8532	0.055	0.945	16.722	17:0 anteiso	2.79	ECL deviates -0.001	Reference -0.003	
11.919	3101	0.046	0.945	16.794	17:1 w8c	1.02	ECL deviates  0.002		
12.085	8341	0.052	0.945	16.890	17:0 cyclo	2.73	ECL deviates  0.002		
12.275	2311	0.052	0.945	16.999	17:0	0.76	ECL deviates -0.001	Reference -0.003	
12.346	3304	0.046	0.945	17.040	16:1 2OH	1.08	ECL deviates -0.008		
12.456	356	0.032	----	17.102		----			
12.996	1858	0.045	0.945	17.409	17:0 10-methyl	0.61	ECL deviates  0.000		
13.151	1263	0.059	----	17.497		----			
13.548	5618	0.047	0.946	17.723	Sum In Feature 5	1.84	ECL deviates  0.003	18:2 w6,9c/18:0 ante	
13.636	22278	0.053	0.946	17.772	18:1 w9c	7.30	ECL deviates  0.003		
13.727	34603	0.051	0.946	17.824	Sum In Feature 8	11.33	ECL deviates  0.001	18:1 w7c	
13.875	3131	0.054	0.946	17.908	18:1 w5c	1.03	ECL deviates -0.011		
14.037	7927	0.047	0.946	18.000	18:0	2.60	ECL deviates  0.000	Reference -0.003	
14.179	2418	0.041	0.946	18.082	18:1 w7c 11-methyl	0.79	ECL deviates  0.001		
14.605	7750	0.059	----	18.325		----			
14.726	12042	0.080	0.947	18.394	18:0 10-methyl, TBSA	----	> max ar/ht		
15.344	1294	0.051	0.948	18.748	Sum In Feature 6	0.42	ECL deviates -0.008	19:1 w11c/19:1 w9c	
15.620	20331	0.051	0.948	18.905	19:0 cyclo w8c	6.67	ECL deviates  0.003		
15.876	264227	0.150	----	19.053		----	> max ar/ht		
16.479	2348	0.050	0.949	19.402	20:4 w6,9,12,15c	0.77	ECL deviates  0.007		
16.620	796	0.041	----	19.483		----			
17.122	1969	0.055	0.949	19.774	20:1 w9c	0.65	ECL deviates  0.004		
17.213	471	0.038	0.950	19.827	20:1 w7c	0.15	ECL deviates -0.004		
17.513	1159	0.046	0.950	20.000	20:0	0.38	ECL deviates  0.000	Reference -0.006	
17.850	937	0.041	----	20.196		----	> max rt		
18.483	1744	0.040	----	20.563		----	> max rt		
----	34091	---	----	----	Summed Feature 3	11.19	16:1 w7c/16:1 w6c	16:1 w6c/16:1 w7c	
----	5618	---	----	----	Summed Feature 5	1.84	18:2 w6,9c/18:0 ante	18:0 ante/18:2 w6,9c	
----	1294	---	----	----	Summed Feature 6	0.42	19:1 w11c/19:1 w9c	19:1 w9c/19:1 w11c	
----	34603	---	----	----	Summed Feature 8	11.33	18:1 w7c	18:1 w6c	
----	37965	---	----	----	Summed Feature 9	12.44	17:1 iso w9c	16:0 10-methyl	

ECL Deviation: 0.004                            Reference ECL Shift: 0.003      Number Reference Peaks: 12
Total Response: 663963                         Total Named: 304488
Percent Named: 45.86%                         Total Amount: 310321
Profile Comment:   Percent named is less than 85.00.

*** No Matches found in TSBA6
