# Supplementary material for: A green garlic (Allium sativum L.) based intercropping system reduces the strain of continuous monocropping in cucumber (Cucumis sativus L.) by adjusting the micro-ecological environment of soil
Source: PeerJ. 2019 Jul 15;7:e7267. doi: 10.7717/peerj.7267 (PMC6637937; doi:10.7717/peerj.7267)
Supplement: Data S1 [file peerj-07-7267-s001.zip › supplemental_Data_S1/30 days after interplanted/CR-3.rtf]

Volume: DATA            File: E131084.29A        Samp Ctr: 9                  ID Number: 1002 
Type: Samp                   Bottle: 3                        Method: TSBA6 
Created: 1/8/2013 1:30:33 PM 
Sample ID: 47 


RT	Response	Ar/Ht	RFact	ECL	Peak Name	Percent	Comment1	Comment2	
1.646	4.532E+8	0.028	----	7.014	SOLVENT PEAK	----	< min rt		
1.778	251	0.007	----	7.273		----	< min rt		
2.285	343	0.026	----	8.267		----	< min rt		
3.059	408	0.029	----	9.787		----			
3.356	377	0.029	----	10.270		----			
4.407	279	0.032	----	11.580		----			
4.581	176	0.024	----	11.768		----			
4.907	1749	0.031	1.021	12.097	11:0 iso 3OH	0.53	ECL deviates  0.008		
5.114	3079	0.036	----	12.275		----			
5.505	648	0.042	1.003	12.613	13:0 iso	0.19	ECL deviates -0.001	Reference -0.005	
6.805	1845	0.039	0.976	13.619	14:0 iso	0.54	ECL deviates  0.000	Reference -0.003	
7.328	2248	0.036	0.969	14.000	14:0	0.65	ECL deviates  0.000	Reference -0.004	
7.778	8228	0.050	----	14.292		----			
8.008	1209	0.040	0.962	14.440	15:1 iso G	0.35	ECL deviates  0.000		
8.293	17046	0.037	0.959	14.624	15:0 iso	4.87	ECL deviates  0.001	Reference -0.002	
8.433	10111	0.039	0.958	14.715	15:0 anteiso	2.89	ECL deviates  0.002	Reference -0.002	
8.639	565	0.041	0.956	14.849	15:1 w6c	0.16	ECL deviates -0.007		
8.877	2172	0.037	0.955	15.002	15:0	----	ECL deviates  0.002		
8.962	731	0.034	----	15.053		----			
9.614	1997	0.056	0.951	15.444	16:1 iso G	0.57	ECL deviates  0.002		
9.921	8709	0.040	0.949	15.627	16:0 iso	2.47	ECL deviates  0.000	Reference -0.003	
10.158	3311	0.053	0.949	15.769	16:1 w9c	0.94	ECL deviates -0.005		
10.239	40181	0.042	0.948	15.818	Sum In Feature 3	11.36	ECL deviates -0.004	16:1 w7c/16:1 w6c	
10.390	8509	0.043	0.948	15.908	16:1 w5c	2.40	ECL deviates -0.001		
10.542	47892	0.043	0.947	15.999	16:0	13.53	ECL deviates -0.001	Reference -0.004	
10.630	629	0.035	----	16.050		----			
11.082	104009	0.060	----	16.311		----			
11.287	49356	0.084	0.946	16.429	Sum In Feature 9	13.92	ECL deviates -0.003	16:0 10-methyl	
11.447	11835	0.085	0.946	16.522	17:1 anteiso w9c	----	> max ar/ht		
11.634	12408	0.058	0.946	16.629	17:0 iso	3.50	ECL deviates -0.001	Reference -0.003	
11.795	10575	0.053	0.945	16.723	17:0 anteiso	2.98	ECL deviates  0.000	Reference -0.003	
11.918	4450	0.052	0.945	16.793	17:1 w8c	1.25	ECL deviates  0.001		
12.084	10637	0.055	0.945	16.889	17:0 cyclo	3.00	ECL deviates  0.001		
12.277	2744	0.055	0.945	17.000	17:0	0.77	ECL deviates  0.000	Reference -0.002	
12.343	3592	0.044	----	17.038		----			
12.993	2118	0.049	0.945	17.406	17:0 10-methyl	0.60	ECL deviates -0.003		
13.143	1363	0.059	----	17.492		----			
13.545	6764	0.047	0.946	17.720	Sum In Feature 5	1.91	ECL deviates  0.000	18:2 w6,9c/18:0 ante	
13.634	21495	0.050	0.946	17.770	18:1 w9c	6.06	ECL deviates  0.001		
13.723	42684	0.053	0.946	17.821	Sum In Feature 8	12.03	ECL deviates -0.002	18:1 w7c	
13.877	3421	0.050	0.946	17.908	18:1 w5c	0.96	ECL deviates -0.011		
14.034	9077	0.046	0.946	17.997	18:0	2.56	ECL deviates -0.003	Reference -0.005	
14.180	2699	0.045	0.946	18.080	18:1 w7c 11-methyl	0.76	ECL deviates -0.001		
14.601	23879	0.064	----	18.320		----			
14.722	18850	0.087	0.947	18.389	18:0 10-methyl, TBSA	----	> max ar/ht		
15.016	363	0.031	----	18.557		----			
15.344	1151	0.051	----	18.744		----			
15.619	21541	0.051	0.948	18.901	19:0 cyclo w8c	6.09	ECL deviates -0.001		
15.875	283661	0.151	----	19.047		----	> max ar/ht		
16.478	2528	0.049	0.949	19.395	20:4 w6,9,12,15c	0.72	ECL deviates  0.000		
16.600	954	0.038	----	19.466		----			
16.907	1306	0.058	0.949	19.643	20:0 iso	0.37	ECL deviates  0.008	Reference  0.009	
17.123	2685	0.069	0.949	19.768	20:1 w9c	0.76	ECL deviates -0.002		
17.516	1155	0.040	0.950	19.994	20:0	0.33	ECL deviates -0.006	Reference -0.004	
17.849	1279	0.047	----	20.187		----	> max rt		
18.173	2161	0.084	----	20.374		----	> max rt		
----	40181	---	----	----	Summed Feature 3	11.36	16:1 w7c/16:1 w6c	16:1 w6c/16:1 w7c	
----	6764	---	----	----	Summed Feature 5	1.91	18:2 w6,9c/18:0 ante	18:0 ante/18:2 w6,9c	
----	42684	---	----	----	Summed Feature 8	12.03	18:1 w7c	18:1 w6c	
----	49356	---	----	----	Summed Feature 9	13.92	17:1 iso w9c	16:0 10-methyl	

ECL Deviation: 0.004                            Reference ECL Shift: 0.004      Number Reference Peaks: 13
Total Response: 817225                         Total Named: 353661
Percent Named: 43.28%                         Total Amount: 366557
Profile Comment:   Percent named is less than 85.00.

*** No Matches found in TSBA6
