# Supplementary material for: A green garlic (Allium sativum L.) based intercropping system reduces the strain of continuous monocropping in cucumber (Cucumis sativus L.) by adjusting the micro-ecological environment of soil
Source: PeerJ. 2019 Jul 15;7:e7267. doi: 10.7717/peerj.7267 (PMC6637937; doi:10.7717/peerj.7267)
Supplement: Data S1 [file peerj-07-7267-s001.zip › supplemental_Data_S1/30 days after interplanted/GB-1.rtf]

Volume: DATA            File: E131095.94A        Samp Ctr: 5                  ID Number: 1013 
Type: Samp                   Bottle: 3                        Method: TSBA6 
Created: 1/9/2013 3:50:48 PM 
Sample ID: 68 


RT	Response	Ar/Ht	RFact	ECL	Peak Name	Percent	Comment1	Comment2	
1.646	4.501E+8	0.028	----	7.014	SOLVENT PEAK	----	< min rt		
1.778	4498	0.023	----	7.274		----	< min rt		
2.033	338	0.024	----	7.774		----	< min rt		
3.062	243	0.026	----	9.794		----			
3.359	256	0.025	----	10.278		----			
4.405	544	0.038	----	11.582		----			
4.790	376	0.032	1.022	11.997	12:0	0.13	ECL deviates -0.003	Reference -0.010	
4.907	2637	0.032	1.019	12.101	11:0 iso 3OH	0.90	ECL deviates  0.012		
5.116	1683	0.035	----	12.281		----			
5.502	430	0.030	1.000	12.613	13:0 iso	0.14	ECL deviates -0.001	Reference -0.007	
6.805	1680	0.036	0.974	13.621	14:0 iso	0.55	ECL deviates  0.002	Reference -0.002	
7.329	2101	0.035	0.966	14.002	14:0	0.68	ECL deviates  0.002	Reference -0.002	
7.783	5216	0.054	----	14.295		----			
8.009	918	0.038	0.959	14.442	15:1 iso G	0.29	ECL deviates  0.002		
8.292	15495	0.038	0.957	14.624	15:0 iso	4.96	ECL deviates  0.001	Reference -0.002	
8.432	9361	0.038	0.956	14.715	15:0 anteiso	2.99	ECL deviates  0.002	Reference -0.002	
8.876	1986	0.042	0.953	15.001	15:0	----	ECL deviates  0.001		
8.965	889	0.039	----	15.055		----			
9.627	1986	0.062	0.949	15.451	16:1 iso G	0.63	ECL deviates  0.009		
9.921	8122	0.041	0.948	15.627	16:0 iso	2.58	ECL deviates  0.000	Reference -0.003	
10.155	2783	0.053	0.947	15.767	16:1 w9c	0.88	ECL deviates -0.007		
10.240	33877	0.043	0.947	15.818	Sum In Feature 3	10.73	ECL deviates -0.004	16:1 w7c/16:1 w6c	
10.391	6951	0.041	0.947	15.908	16:1 w5c	2.20	ECL deviates -0.001		
10.542	44947	0.042	0.946	15.999	16:0	14.23	ECL deviates -0.001	Reference -0.004	
10.626	384	0.034	----	16.047		----			
11.081	73428	0.062	----	16.310		----			
11.286	41926	0.082	0.945	16.428	Sum In Feature 9	13.25	ECL deviates -0.004	16:0 10-methyl	
11.439	9376	0.086	0.945	16.517	17:1 anteiso w9c	----	> max ar/ht		
11.634	9740	0.053	0.945	16.629	17:0 iso	3.08	ECL deviates -0.001	Reference -0.004	
11.794	8412	0.052	0.945	16.721	17:0 anteiso	2.66	ECL deviates -0.002	Reference -0.004	
11.916	3907	0.057	0.945	16.792	17:1 w8c	1.23	ECL deviates  0.000		
12.084	8351	0.050	0.945	16.888	17:0 cyclo	2.64	ECL deviates  0.000		
12.277	2106	0.048	0.945	17.000	17:0	0.67	ECL deviates  0.000	Reference -0.002	
12.345	3277	0.046	0.945	17.039	16:1 2OH	1.04	ECL deviates -0.009		
12.995	1965	0.046	0.945	17.407	17:0 10-methyl	0.62	ECL deviates -0.002		
13.151	1234	0.053	----	17.495		----			
13.546	13308	0.046	0.946	17.719	Sum In Feature 5	4.21	ECL deviates -0.001	18:2 w6,9c/18:0 ante	
13.635	21567	0.052	0.946	17.770	18:1 w9c	6.82	ECL deviates  0.001		
13.725	30935	0.051	0.946	17.821	Sum In Feature 8	9.79	ECL deviates -0.002	18:1 w7c	
13.880	3087	0.055	0.946	17.909	18:1 w5c	0.98	ECL deviates -0.010		
14.036	8291	0.044	0.947	17.997	18:0	2.62	ECL deviates -0.003	Reference -0.005	
14.176	2524	0.045	0.947	18.078	18:1 w7c 11-methyl	0.80	ECL deviates -0.003		
14.604	17940	0.066	----	18.322		----			
14.727	15614	0.087	0.948	18.393	18:0 10-methyl, TBSA	----	> max ar/ht		
15.347	1020	0.051	0.949	18.747	Sum In Feature 6	0.32	ECL deviates -0.009	19:1 w11c/19:1 w9c	
15.619	18758	0.050	0.949	18.903	19:0 cyclo w8c	5.95	ECL deviates  0.001		
15.900	281248	0.148	----	19.064		----	> max ar/ht		
16.475	1181	0.038	0.950	19.396	20:4 w6,9,12,15c	0.38	ECL deviates  0.001		
17.120	1339	0.049	0.951	19.769	20:1 w9c	0.43	ECL deviates -0.001		
17.222	492	0.037	0.951	19.828	20:1 w7c	0.16	ECL deviates -0.003		
17.524	1469	0.056	0.951	20.002	20:0	0.47	ECL deviates  0.002	Reference  0.000	
17.850	1158	0.046	----	20.191		----	> max rt		
18.178	1920	0.101	----	20.380		----	> max rt		
----	33877	---	----	----	Summed Feature 3	10.73	16:1 w7c/16:1 w6c	16:1 w6c/16:1 w7c	
----	13308	---	----	----	Summed Feature 5	4.21	18:2 w6,9c/18:0 ante	18:0 ante/18:2 w6,9c	
----	1020	---	----	----	Summed Feature 6	0.32	19:1 w11c/19:1 w9c	19:1 w9c/19:1 w11c	
----	30935	---	----	----	Summed Feature 8	9.79	18:1 w7c	18:1 w6c	
----	41926	---	----	----	Summed Feature 9	13.25	17:1 iso w9c	16:0 10-methyl	

ECL Deviation: 0.004                            Reference ECL Shift: 0.004      Number Reference Peaks: 13
Total Response: 723377                         Total Named: 315321
Percent Named: 43.59%                         Total Amount: 324582
Profile Comment:   Percent named is less than 85.00.

*** No Matches found in TSBA6
