# Supplementary material for: A green garlic (Allium sativum L.) based intercropping system reduces the strain of continuous monocropping in cucumber (Cucumis sativus L.) by adjusting the micro-ecological environment of soil
Source: PeerJ. 2019 Jul 15;7:e7267. doi: 10.7717/peerj.7267 (PMC6637937; doi:10.7717/peerj.7267)
Supplement: Data S1 [file peerj-07-7267-s001.zip › supplemental_Data_S1/30 days after interplanted/GB-2.rtf]

Volume: DATA            File: E131095.94A        Samp Ctr: 6                  ID Number: 1014 
Type: Samp                   Bottle: 4                        Method: TSBA6 
Created: 1/9/2013 4:15:00 PM 
Sample ID: 69 


RT	Response	Ar/Ht	RFact	ECL	Peak Name	Percent	Comment1	Comment2	
1.645	4.52E+8	0.029	----	7.007	SOLVENT PEAK	----	< min rt		
1.777	2675	0.022	----	7.265		----	< min rt		
2.035	249	0.029	----	7.773		----	< min rt		
4.408	495	0.035	----	11.583		----			
4.908	1948	0.032	1.019	12.098	11:0 iso 3OH	0.61	ECL deviates  0.009		
5.119	1713	0.042	----	12.280		----			
5.503	413	0.029	1.000	12.612	13:0 iso	0.13	ECL deviates -0.002	Reference -0.005	
6.805	1586	0.031	0.974	13.620	14:0 iso	0.48	ECL deviates  0.001	Reference -0.003	
7.329	2136	0.035	0.966	14.000	14:0	0.64	ECL deviates  0.000	Reference -0.003	
7.788	4752	0.056	----	14.298		----			
8.010	973	0.037	0.959	14.441	15:1 iso G	0.29	ECL deviates  0.001		
8.293	16565	0.036	0.957	14.624	15:0 iso	4.89	ECL deviates  0.001	Reference -0.002	
8.432	9725	0.040	0.956	14.714	15:0 anteiso	2.87	ECL deviates  0.001	Reference -0.002	
8.877	1820	0.037	0.953	15.002	15:0	----	ECL deviates  0.002		
8.968	708	0.031	----	15.056		----			
9.632	2118	0.067	0.949	15.454	16:1 iso H	0.62	ECL deviates -0.007		
9.921	8940	0.041	0.948	15.627	16:0 iso	2.61	ECL deviates  0.000	Reference -0.003	
10.156	2077	0.038	0.947	15.768	16:1 w9c	0.61	ECL deviates -0.006		
10.239	34342	0.042	0.947	15.818	Sum In Feature 3	10.03	ECL deviates -0.004	16:1 w7c/16:1 w6c	
10.390	7114	0.041	0.947	15.908	16:1 w5c	2.08	ECL deviates -0.001		
10.542	45685	0.042	0.946	15.999	16:0	13.33	ECL deviates -0.001	Reference -0.004	
10.632	686	0.040	----	16.051		----			
11.082	49541	0.070	----	16.311		----			
11.289	41543	0.076	0.945	16.430	Sum In Feature 9	12.11	ECL deviates -0.002	16:0 10-methyl	
11.449	7958	0.079	0.945	16.523	17:1 anteiso w9c	2.32	ECL deviates -0.001		
11.634	10306	0.052	0.945	16.630	17:0 iso	3.00	ECL deviates  0.000	Reference -0.003	
11.795	9391	0.053	0.945	16.723	17:0 anteiso	2.74	ECL deviates  0.000	Reference -0.004	
11.918	3970	0.054	0.945	16.793	17:1 w8c	1.16	ECL deviates  0.001		
12.085	8997	0.052	0.945	16.890	17:0 cyclo	2.62	ECL deviates  0.002		
12.275	2537	0.050	0.945	16.999	17:0	0.74	ECL deviates -0.001	Reference -0.004	
12.344	3741	0.046	0.945	17.039	16:1 2OH	1.09	ECL deviates -0.009		
12.997	2232	0.044	0.945	17.409	17:0 10-methyl	0.65	ECL deviates  0.000		
13.145	1126	0.047	----	17.493		----			
13.546	15918	0.045	0.946	17.721	Sum In Feature 5	4.64	ECL deviates  0.001	18:2 w6,9c/18:0 ante	
13.635	21746	0.049	0.946	17.771	18:1 w9c	6.34	ECL deviates  0.002		
13.726	32232	0.051	0.946	17.823	Sum In Feature 8	9.41	ECL deviates  0.000	18:1 w7c	
13.879	3337	0.053	0.946	17.910	18:1 w5c	0.97	ECL deviates -0.009		
14.037	8675	0.047	0.947	17.999	18:0	2.53	ECL deviates -0.001	Reference -0.004	
14.179	2806	0.049	0.947	18.081	18:1 w7c 11-methyl	0.82	ECL deviates  0.000		
14.609	7154	0.070	----	18.327		----			
14.727	8363	0.056	0.948	18.394	18:0 10-methyl, TBSA	2.44	ECL deviates  0.002		
14.788	4330	0.046	----	18.429		----			
15.346	1144	0.054	0.949	18.748	Sum In Feature 6	0.33	ECL deviates -0.008	19:1 w11c/19:1 w9c	
15.618	19138	0.050	0.949	18.904	19:0 cyclo w8c	5.60	ECL deviates  0.002		
15.898	307544	0.149	----	19.064		----	> max ar/ht		
16.476	1361	0.043	0.950	19.398	20:4 w6,9,12,15c	0.40	ECL deviates  0.003		
16.602	406	0.027	----	19.471		----			
17.117	1492	0.047	0.951	19.769	20:1 w9c	0.44	ECL deviates -0.001		
17.518	1573	0.046	0.951	20.001	20:0	0.46	ECL deviates  0.001	Reference -0.004	
17.852	1093	0.043	----	20.194		----	> max rt		
----	34342	---	----	----	Summed Feature 3	10.03	16:1 w7c/16:1 w6c	16:1 w6c/16:1 w7c	
----	15918	---	----	----	Summed Feature 5	4.64	18:2 w6,9c/18:0 ante	18:0 ante/18:2 w6,9c	
----	1144	---	----	----	Summed Feature 6	0.33	19:1 w11c/19:1 w9c	19:1 w9c/19:1 w11c	
----	32232	---	----	----	Summed Feature 8	9.41	18:1 w7c	18:1 w6c	
----	41543	---	----	----	Summed Feature 9	12.11	17:1 iso w9c	16:0 10-methyl	

ECL Deviation: 0.004                            Reference ECL Shift: 0.003      Number Reference Peaks: 12
Total Response: 720538                         Total Named: 342082
Percent Named: 47.48%                         Total Amount: 326023
Profile Comment:   Percent named is less than 85.00.

*** No Matches found in TSBA6
