# Supplementary material for: A green garlic (Allium sativum L.) based intercropping system reduces the strain of continuous monocropping in cucumber (Cucumis sativus L.) by adjusting the micro-ecological environment of soil
Source: PeerJ. 2019 Jul 15;7:e7267. doi: 10.7717/peerj.7267 (PMC6637937; doi:10.7717/peerj.7267)
Supplement: Data S1 [file peerj-07-7267-s001.zip › supplemental_Data_S1/30 days after interplanted/GB-3.rtf]

Volume: DATA            File: E131095.94A        Samp Ctr: 7                  ID Number: 1015 
Type: Samp                   Bottle: 5                        Method: TSBA6 
Created: 1/9/2013 4:39:13 PM 
Sample ID: 70 


RT	Response	Ar/Ht	RFact	ECL	Peak Name	Percent	Comment1	Comment2	
1.646	4.548E+8	0.028	----	7.003	SOLVENT PEAK	----	< min rt		
1.778	2804	0.023	----	7.262		----	< min rt		
2.031	182	0.023	----	7.759		----	< min rt		
3.357	463	0.032	----	10.264		----			
4.007	215	0.025	1.061	11.152	10:0 2OH	0.07	ECL deviates -0.001		
4.778	548	0.038	----	11.982		----			
4.908	2358	0.034	1.019	12.099	11:0 iso 3OH	0.70	ECL deviates  0.010		
5.116	3611	0.039	----	12.279		----			
5.504	498	0.027	1.000	12.613	13:0 iso	0.14	ECL deviates -0.001	Reference -0.005	
6.807	1623	0.033	0.974	13.620	14:0 iso	0.46	ECL deviates  0.001	Reference -0.001	
7.330	2306	0.036	0.966	14.001	14:0	0.65	ECL deviates  0.001	Reference -0.002	
7.783	7085	0.049	----	14.293		----			
8.009	1165	0.042	0.959	14.440	15:1 iso G	0.32	ECL deviates  0.000		
8.293	17426	0.038	0.957	14.623	15:0 iso	4.83	ECL deviates  0.000	Reference -0.002	
8.432	10358	0.041	0.956	14.713	15:0 anteiso	2.87	ECL deviates  0.000	Reference -0.002	
8.874	1954	0.037	0.953	14.999	15:0	----	ECL deviates -0.001		
8.965	755	0.034	----	15.054		----			
9.627	2378	0.063	0.949	15.450	16:1 iso G	0.65	ECL deviates  0.008		
9.921	9260	0.041	0.948	15.627	16:0 iso	2.54	ECL deviates  0.000	Reference -0.003	
10.160	3004	0.050	0.947	15.769	16:1 w9c	0.82	ECL deviates -0.005		
10.240	37377	0.044	0.947	15.817	Sum In Feature 3	10.26	ECL deviates -0.005	16:1 w7c/16:1 w6c	
10.391	7901	0.042	0.947	15.908	16:1 w5c	2.17	ECL deviates -0.001		
10.544	50134	0.042	0.946	16.000	16:0	13.75	ECL deviates  0.000	Reference -0.002	
11.082	74489	0.061	----	16.310		----			
11.289	46023	0.084	0.945	16.430	Sum In Feature 9	12.61	ECL deviates -0.002	16:0 10-methyl	
11.441	10788	0.084	0.945	16.518	17:1 anteiso w9c	----	> max ar/ht		
11.635	11778	0.057	0.945	16.629	17:0 iso	3.23	ECL deviates -0.001	Reference -0.003	
11.795	10622	0.053	0.945	16.722	17:0 anteiso	2.91	ECL deviates -0.001	Reference -0.003	
11.919	4730	0.056	0.945	16.794	17:1 w8c	1.30	ECL deviates  0.002		
12.085	10631	0.056	0.945	16.889	17:0 cyclo	2.91	ECL deviates  0.001		
12.278	2263	0.039	0.945	17.001	17:0	0.62	ECL deviates  0.001	Reference -0.002	
12.345	3755	0.043	0.945	17.039	16:1 2OH	1.03	ECL deviates -0.009		
12.991	2001	0.042	0.945	17.406	17:0 10-methyl	0.55	ECL deviates -0.003		
13.146	723	0.036	----	17.494		----			
13.547	15445	0.046	0.946	17.722	Sum In Feature 5	4.23	ECL deviates  0.002	18:2 w6,9c/18:0 ante	
13.634	26174	0.057	0.946	17.771	18:1 w9c	7.17	ECL deviates  0.002		
13.725	32598	0.048	0.946	17.823	Sum In Feature 8	8.94	ECL deviates  0.000	18:1 w7c	
13.883	3770	0.066	0.946	17.913	18:1 w5c	1.03	ECL deviates -0.006		
14.036	9288	0.048	0.947	18.000	18:0	2.55	ECL deviates  0.000	Reference -0.005	
14.179	2748	0.050	0.947	18.082	18:1 w7c 11-methyl	0.75	ECL deviates  0.001		
14.602	12790	0.068	----	18.324		----			
14.726	10153	0.061	0.948	18.395	18:0 10-methyl, TBSA	2.79	ECL deviates  0.003		
14.788	4469	0.043	----	18.430		----			
15.348	1659	0.056	0.949	18.751	Sum In Feature 6	0.46	ECL deviates -0.005	19:1 w11c/19:1 w9c	
15.620	20584	0.051	0.949	18.907	19:0 cyclo w8c	5.66	ECL deviates  0.005		
15.862	289117	0.145	----	19.046		----	> max ar/ht		
16.475	1183	0.041	0.950	19.401	20:4 w6,9,12,15c	0.33	ECL deviates  0.006		
17.123	1451	0.047	0.951	19.776	20:1 w9c	0.40	ECL deviates  0.006		
17.510	1173	0.042	0.951	20.000	20:0	0.32	ECL deviates  0.000	Reference -0.009	
17.854	954	0.040	----	20.200		----	> max rt		
18.177	1029	0.065	----	20.387		----	> max rt		
----	37377	---	----	----	Summed Feature 3	10.26	16:1 w7c/16:1 w6c	16:1 w6c/16:1 w7c	
----	15445	---	----	----	Summed Feature 5	4.23	18:2 w6,9c/18:0 ante	18:0 ante/18:2 w6,9c	
----	1659	---	----	----	Summed Feature 6	0.46	19:1 w11c/19:1 w9c	19:1 w9c/19:1 w11c	
----	32598	---	----	----	Summed Feature 8	8.94	18:1 w7c	18:1 w6c	
----	46023	---	----	----	Summed Feature 9	12.61	17:1 iso w9c	16:0 10-methyl	

ECL Deviation: 0.004                            Reference ECL Shift: 0.004      Number Reference Peaks: 12
Total Response: 768873                         Total Named: 364034
Percent Named: 47.35%                         Total Amount: 357212
Profile Comment:   Percent named is less than 85.00.

*** No Matches found in TSBA6
