# Supplementary material for: A green garlic (Allium sativum L.) based intercropping system reduces the strain of continuous monocropping in cucumber (Cucumis sativus L.) by adjusting the micro-ecological environment of soil
Source: PeerJ. 2019 Jul 15;7:e7267. doi: 10.7717/peerj.7267 (PMC6637937; doi:10.7717/peerj.7267)
Supplement: Data S1 [file peerj-07-7267-s001.zip › supplemental_Data_S1/30 days after interplanted/GR-1.rtf]

Volume: DATA            File: E131074.05A        Samp Ctr: 6                  ID Number: 1004 
Type: Samp                   Bottle: 5                        Method: TSBA6 
Created: 1/7/2013 11:43:16 AM 
Sample ID: 36 


RT	Response	Ar/Ht	RFact	ECL	Peak Name	Percent	Comment1	Comment2	
1.645	4.536E+8	0.029	----	7.013	SOLVENT PEAK	----	< min rt		
1.778	3565	0.023	----	7.272		----	< min rt		
2.285	381	0.022	----	8.267		----	< min rt		
4.907	1645	0.034	1.019	12.102	11:0 iso 3OH	0.51	ECL deviates  0.013		
5.131	735	0.044	----	12.295		----			
5.499	371	0.034	1.000	12.612	13:0 iso	0.11	ECL deviates -0.002	Reference -0.012	
6.806	1458	0.035	0.973	13.621	14:0 iso	0.44	ECL deviates  0.002	Reference -0.005	
7.331	1855	0.035	0.966	14.001	14:0	0.55	ECL deviates  0.001	Reference -0.005	
7.799	2509	0.045	----	14.305		----			
8.012	1147	0.040	0.959	14.442	15:1 iso G	0.34	ECL deviates  0.002		
8.294	15155	0.037	0.957	14.624	15:0 iso	4.45	ECL deviates  0.001	Reference -0.004	
8.433	9336	0.039	0.956	14.714	15:0 anteiso	2.74	ECL deviates  0.001	Reference -0.004	
8.876	1987	0.039	0.953	15.000	15:0	----	ECL deviates  0.000		
8.967	759	0.038	----	15.055		----			
9.618	2444	0.062	0.949	15.444	16:1 iso G	0.71	ECL deviates  0.002		
9.922	9078	0.041	0.948	15.626	16:0 iso	2.64	ECL deviates -0.001	Reference -0.004	
10.160	2776	0.054	0.947	15.769	16:1 w9c	0.81	ECL deviates -0.005		
10.241	36224	0.043	0.947	15.817	Sum In Feature 3	10.52	ECL deviates -0.005	16:1 w7c/16:1 w6c	
10.392	7357	0.043	0.947	15.908	16:1 w5c	2.14	ECL deviates -0.001		
10.545	46654	0.041	0.946	15.999	16:0	13.54	ECL deviates -0.001	Reference -0.004	
10.630	619	0.036	----	16.048		----			
11.085	24535	0.071	----	16.311		----			
11.290	36870	0.076	0.945	16.429	Sum In Feature 9	10.69	ECL deviates -0.003	16:0 10-methyl	
11.449	6482	0.072	0.945	16.520	17:1 anteiso w9c	1.88	ECL deviates -0.004		
11.636	9387	0.047	0.945	16.629	17:0 iso	2.72	ECL deviates -0.001	Reference -0.004	
11.797	8711	0.047	0.945	16.722	17:0 anteiso	2.52	ECL deviates -0.001	Reference -0.004	
11.920	3628	0.051	0.945	16.792	17:1 w8c	1.05	ECL deviates  0.000		
12.085	8630	0.051	0.945	16.888	17:0 cyclo	2.50	ECL deviates  0.000		
12.276	2043	0.043	0.945	16.998	17:0	0.59	ECL deviates -0.002	Reference -0.005	
12.346	3546	0.042	----	17.038		----			
12.995	2572	0.051	0.945	17.406	17:0 10-methyl	0.75	ECL deviates -0.003		
13.150	1679	0.053	----	17.493		----			
13.388	1571	0.106	0.946	17.629	18:0 iso	----	> max ar/ht		
13.549	11232	0.048	0.946	17.720	Sum In Feature 5	3.26	ECL deviates  0.000	18:2 w6,9c/18:0 ante	
13.637	25002	0.054	0.946	17.770	18:1 w9c	7.26	ECL deviates  0.001		
13.727	40803	0.054	0.946	17.821	Sum In Feature 8	11.84	ECL deviates -0.002	18:1 w7c	
13.881	4298	0.063	0.947	17.908	18:1 w5c	1.25	ECL deviates -0.011		
14.039	9750	0.049	0.947	17.997	18:0	2.83	ECL deviates -0.003	Reference -0.005	
14.179	3205	0.050	0.947	18.078	18:1 w7c 11-methyl	0.93	ECL deviates -0.003		
14.625	4420	0.069	----	18.332		----			
14.730	7858	0.053	0.948	18.393	18:0 10-methyl, TBSA	2.28	ECL deviates  0.001		
14.789	4188	0.043	----	18.426		----			
15.026	748	0.042	----	18.562		----			
15.145	265	0.028	0.948	18.630	19:0 iso	0.08	ECL deviates -0.004	Reference -0.006	
15.343	1172	0.039	----	18.744		----		Reference  0.010	
15.620	21048	0.047	0.949	18.902	19:0 cyclo w8c	6.13	ECL deviates  0.000		
15.894	296081	0.146	----	19.059		----	> max ar/ht		
16.479	1818	0.043	0.950	19.397	20:4 w6,9,12,15c	0.53	ECL deviates  0.002		
16.615	1044	0.050	----	19.475		----			
17.124	3540	0.076	0.951	19.770	20:1 w9c	1.03	ECL deviates  0.000		
17.518	1332	0.049	0.951	19.997	20:0	0.39	ECL deviates -0.003	Reference -0.007	
17.852	1203	0.039	----	20.190		----	> max rt		
18.185	1414	0.056	----	20.382		----	> max rt		
----	36224	---	----	----	Summed Feature 3	10.52	16:1 w7c/16:1 w6c	16:1 w6c/16:1 w7c	
----	11232	---	----	----	Summed Feature 5	3.26	18:2 w6,9c/18:0 ante	18:0 ante/18:2 w6,9c	
----	40803	---	----	----	Summed Feature 8	11.84	18:1 w7c	18:1 w6c	
----	36870	---	----	----	Summed Feature 9	10.69	17:1 iso w9c	16:0 10-methyl	

ECL Deviation: 0.004                            Reference ECL Shift: 0.006      Number Reference Peaks: 14
Total Response: 687579                         Total Named: 343973
Percent Named: 50.03%                         Total Amount: 329421
Profile Comment:   Percent named is less than 85.00.

*** No Matches found in TSBA6
