# Supplementary material for: A green garlic (Allium sativum L.) based intercropping system reduces the strain of continuous monocropping in cucumber (Cucumis sativus L.) by adjusting the micro-ecological environment of soil
Source: PeerJ. 2019 Jul 15;7:e7267. doi: 10.7717/peerj.7267 (PMC6637937; doi:10.7717/peerj.7267)
Supplement: Data S1 [file peerj-07-7267-s001.zip › supplemental_Data_S1/30 days after interplanted/GR-2.rtf]

Volume: DATA            File: E131074.05A        Samp Ctr: 11                ID Number: 1008 
Type: Samp                   Bottle: 6                        Method: TSBA6 
Created: 1/7/2013 1:44:40 PM 
Sample ID: 37 


RT	Response	Ar/Ht	RFact	ECL	Peak Name	Percent	Comment1	Comment2	
1.645	4.547E+8	0.029	----	7.006	SOLVENT PEAK	----	< min rt		
1.778	-----	---	----	7.266		----	< min rt		
2.285	405	0.031	----	8.261		----	< min rt		
3.060	189	0.021	----	9.784		----			
4.409	476	0.040	----	11.588		----			
4.907	2229	0.031	1.019	12.101	11:0 iso 3OH	0.62	ECL deviates  0.012		
5.116	1645	0.035	----	12.280		----			
5.502	885	0.044	1.000	12.613	13:0 iso	0.24	ECL deviates -0.001	Reference -0.010	
6.400	485	0.033	----	13.326		----			
6.807	1817	0.038	0.973	13.621	14:0 iso	0.48	ECL deviates  0.002	Reference -0.004	
7.329	2352	0.035	0.966	14.000	14:0	0.62	ECL deviates  0.000	Reference -0.006	
7.785	5761	0.051	----	14.295		----			
8.010	1361	0.043	0.959	14.440	15:1 iso G	0.36	ECL deviates  0.000		
8.293	19770	0.040	0.957	14.623	15:0 iso	5.18	ECL deviates  0.000	Reference -0.005	
8.434	11592	0.039	0.956	14.714	15:0 anteiso	3.03	ECL deviates  0.001	Reference -0.003	
8.877	2431	0.039	0.953	15.001	15:0	----	ECL deviates  0.001		
8.967	905	0.035	----	15.054		----			
9.631	2126	0.058	0.949	15.452	16:1 iso H	0.55	ECL deviates -0.009		
9.923	11152	0.039	0.948	15.627	16:0 iso	2.90	ECL deviates  0.000	Reference -0.004	
10.160	3134	0.051	0.947	15.769	16:1 w9c	0.81	ECL deviates -0.005		
10.241	40667	0.043	0.947	15.818	Sum In Feature 3	10.55	ECL deviates -0.004	16:1 w7c/16:1 w6c	
10.392	8064	0.041	0.947	15.908	16:1 w5c	2.09	ECL deviates -0.001		
10.544	51658	0.041	0.946	15.999	16:0	13.39	ECL deviates -0.001	Reference -0.005	
10.629	509	0.029	----	16.048		----			
11.081	70181	0.059	----	16.310		----			
11.289	46581	0.078	0.945	16.430	Sum In Feature 9	12.06	ECL deviates -0.002	16:0 10-methyl	
11.450	10504	0.085	0.945	16.522	17:1 anteiso w9c	----	> max ar/ht		
11.636	12440	0.049	0.945	16.630	17:0 iso	3.22	ECL deviates  0.000	Reference -0.004	
11.797	11036	0.051	0.945	16.723	17:0 anteiso	2.86	ECL deviates  0.000	Reference -0.004	
11.919	4759	0.054	0.945	16.793	17:1 w8c	1.23	ECL deviates  0.001		
12.086	10490	0.054	0.945	16.890	17:0 cyclo	2.71	ECL deviates  0.002		
12.277	2231	0.045	0.945	17.000	17:0	0.58	ECL deviates  0.000	Reference -0.004	
12.348	4671	0.047	0.945	17.040	16:1 2OH	1.21	ECL deviates -0.008		
12.991	3649	0.052	0.945	17.405	17:0 10-methyl	0.94	ECL deviates -0.004		
13.149	1778	0.049	----	17.495		----			
13.548	8683	0.045	0.946	17.721	Sum In Feature 5	2.25	ECL deviates  0.001	18:2 w6,9c/18:0 ante	
13.637	28057	0.057	0.946	17.772	18:1 w9c	7.27	ECL deviates  0.003		
13.727	37585	0.050	0.946	17.823	Sum In Feature 8	9.74	ECL deviates  0.000	18:1 w7c	
13.877	4358	0.053	0.947	17.908	18:1 w5c	1.13	ECL deviates -0.011		
14.037	10448	0.048	0.947	17.999	18:0	2.71	ECL deviates -0.001	Reference -0.006	
14.177	2567	0.048	0.947	18.079	18:1 w7c 11-methyl	0.67	ECL deviates -0.002		
14.321	639	0.054	0.947	18.162	17:0 iso 3OH	0.17	ECL deviates  0.001		
14.607	18101	0.064	----	18.326		----			
14.728	13158	0.058	0.948	18.395	18:0 10-methyl, TBSA	3.42	ECL deviates  0.003		
14.788	6148	0.049	----	18.429		----			
15.022	651	0.044	----	18.563		----			
15.347	2110	0.053	0.949	18.750	Sum In Feature 6	0.55	ECL deviates -0.006	19:1 w11c/19:1 w9c	
15.622	18910	0.048	0.949	18.907	19:0 cyclo w8c	4.92	ECL deviates  0.005		
15.902	327601	0.149	----	19.068		----	> max ar/ht		
16.478	1650	0.043	0.950	19.401	20:4 w6,9,12,15c	0.43	ECL deviates  0.006		
16.608	442	0.033	----	19.476		----			
17.122	2174	0.058	0.951	19.774	20:1 w9c	0.57	ECL deviates  0.004		
17.227	514	0.035	0.951	19.834	20:1 w7c	0.13	ECL deviates  0.003		
17.515	1624	0.040	0.951	20.001	20:0	0.42	ECL deviates  0.001	Reference -0.009	
17.850	1410	0.043	----	20.195		----	> max rt		
18.182	1905	0.063	----	20.386		----	> max rt		
----	40667	---	----	----	Summed Feature 3	10.55	16:1 w7c/16:1 w6c	16:1 w6c/16:1 w7c	
----	8683	---	----	----	Summed Feature 5	2.25	18:2 w6,9c/18:0 ante	18:0 ante/18:2 w6,9c	
----	2110	---	----	----	Summed Feature 6	0.55	19:1 w11c/19:1 w9c	19:1 w9c/19:1 w11c	
----	37585	---	----	----	Summed Feature 8	9.74	18:1 w7c	18:1 w6c	
----	46581	---	----	----	Summed Feature 9	12.06	17:1 iso w9c	16:0 10-methyl	

ECL Deviation: 0.004                            Reference ECL Shift: 0.006      Number Reference Peaks: 12
Total Response: 830515                         Total Named: 385139
Percent Named: 46.37%                         Total Amount: 377391
Profile Comment:   Percent named is less than 85.00.

*** No Matches found in TSBA6
