# Supplementary material for: A green garlic (Allium sativum L.) based intercropping system reduces the strain of continuous monocropping in cucumber (Cucumis sativus L.) by adjusting the micro-ecological environment of soil
Source: PeerJ. 2019 Jul 15;7:e7267. doi: 10.7717/peerj.7267 (PMC6637937; doi:10.7717/peerj.7267)
Supplement: Data S1 [file peerj-07-7267-s001.zip › supplemental_Data_S1/30 days after interplanted/GR-3.rtf]

Volume: DATA            File: E131074.05A        Samp Ctr: 12                ID Number: 1009 
Type: Samp                   Bottle: 7                        Method: TSBA6 
Created: 1/7/2013 2:08:45 PM 
Sample ID: 38 


RT	Response	Ar/Ht	RFact	ECL	Peak Name	Percent	Comment1	Comment2	
1.645	4.548E+8	0.029	----	7.008	SOLVENT PEAK	----	< min rt		
1.778	3914	0.024	----	7.268		----	< min rt		
2.284	305	0.023	----	8.260		----	< min rt		
4.006	220	0.033	1.061	11.155	10:0 2OH	0.04	ECL deviates  0.002		
4.907	2577	0.033	1.019	12.101	11:0 iso 3OH	0.50	ECL deviates  0.012		
5.130	716	0.037	----	12.293		----			
5.501	317	0.026	1.000	12.612	13:0 iso	0.06	ECL deviates -0.002	Reference -0.010	
6.400	325	0.029	----	13.326		----			
6.806	1641	0.036	0.973	13.621	14:0 iso	0.31	ECL deviates  0.002	Reference -0.004	
7.330	2185	0.036	0.966	14.000	14:0	0.40	ECL deviates  0.000	Reference -0.005	
7.802	2585	0.050	----	14.306		----			
8.010	1308	0.041	0.959	14.440	15:1 iso G	0.24	ECL deviates  0.000		
8.294	17489	0.038	0.957	14.624	15:0 iso	3.20	ECL deviates  0.001	Reference -0.004	
8.434	10717	0.041	0.956	14.714	15:0 anteiso	1.96	ECL deviates  0.001	Reference -0.003	
8.641	820	0.051	0.954	14.848	15:1 w6c	0.15	ECL deviates -0.008		
8.877	2267	0.039	0.953	15.000	15:0	----	ECL deviates  0.000		
8.968	824	0.038	----	15.055		----			
9.625	2525	0.066	0.949	15.448	16:1 iso G	0.46	ECL deviates  0.006		
9.924	9999	0.040	0.948	15.627	16:0 iso	1.81	ECL deviates  0.000	Reference -0.003	
10.160	3166	0.054	0.947	15.769	16:1 w9c	0.57	ECL deviates -0.005		
10.241	40155	0.044	0.947	15.817	Sum In Feature 3	7.26	ECL deviates -0.005	16:1 w7c/16:1 w6c	
10.391	8224	0.043	0.947	15.907	16:1 w5c	1.49	ECL deviates -0.002		
10.545	51103	0.042	0.946	15.999	16:0	9.24	ECL deviates -0.001	Reference -0.004	
10.630	774	0.039	----	16.048		----			
11.085	24389	0.072	----	16.311		----			
11.289	39382	0.074	0.945	16.429	Sum In Feature 9	7.11	ECL deviates -0.003	16:0 10-methyl	
11.455	6540	0.074	0.945	16.525	17:1 anteiso w9c	1.18	ECL deviates  0.001		
11.636	9526	0.047	0.945	16.629	17:0 iso	1.72	ECL deviates -0.001	Reference -0.004	
11.796	9411	0.049	0.945	16.722	17:0 anteiso	1.70	ECL deviates -0.001	Reference -0.005	
11.920	4043	0.053	0.945	16.793	17:1 w8c	0.73	ECL deviates  0.001		
12.085	9896	0.053	0.945	16.889	17:0 cyclo	1.79	ECL deviates  0.001		
12.280	2066	0.038	0.945	17.001	17:0	0.37	ECL deviates  0.001	Reference -0.002	
12.346	4276	0.043	0.945	17.038	16:1 2OH	0.77	ECL deviates -0.010		
12.466	411	0.038	----	17.107		----			
12.994	2861	0.049	0.945	17.406	17:0 10-methyl	0.52	ECL deviates -0.003		
13.148	1394	0.050	----	17.493		----			
13.548	12478	0.045	0.946	17.721	Sum In Feature 5	2.25	ECL deviates  0.001	18:2 w6,9c/18:0 ante	
13.637	25956	0.050	0.946	17.771	18:1 w9c	4.69	ECL deviates  0.002		
13.725	43289	0.053	0.946	17.821	Sum In Feature 8	7.82	ECL deviates -0.002	18:1 w7c	
13.876	3841	0.060	----	17.907		----			
14.038	9834	0.044	0.947	17.998	18:0	1.78	ECL deviates -0.002	Reference -0.006	
14.179	3050	0.046	0.947	18.080	18:1 w7c 11-methyl	0.55	ECL deviates -0.001		
14.628	4063	0.059	----	18.336		----			
14.730	8281	0.051	0.948	18.395	18:0 10-methyl, TBSA	1.50	ECL deviates  0.003		
14.788	4149	0.041	----	18.428		----			
15.344	1961	0.054	0.949	18.747	Sum In Feature 6	0.36	ECL deviates -0.009	19:1 w11c/19:1 w9c	
15.620	22198	0.051	0.949	18.905	19:0 cyclo w8c	4.02	ECL deviates  0.003		
15.909	206329	0.087	----	19.071		----	> max ar/ht		
15.935	179038	0.070	0.950	19.086	18:1 2OH	32.47	ECL deviates -0.003		
16.476	1679	0.042	0.950	19.399	20:4 w6,9,12,15c	0.30	ECL deviates  0.004		
16.619	502	0.036	----	19.482		----			
16.774	490	0.035	----	19.571		----			
17.125	1762	0.044	0.951	19.774	20:1 w9c	0.32	ECL deviates  0.004		
17.224	332	0.028	0.951	19.831	20:1 w7c	0.06	ECL deviates  0.000		
17.518	1644	0.050	0.951	20.001	20:0	0.30	ECL deviates  0.001	Reference -0.007	
17.851	1264	0.047	----	20.193		----	> max rt		
18.183	754	0.053	----	20.386		----	> max rt		
----	40155	---	----	----	Summed Feature 3	7.26	16:1 w7c/16:1 w6c	16:1 w6c/16:1 w7c	
----	12478	---	----	----	Summed Feature 5	2.25	18:2 w6,9c/18:0 ante	18:0 ante/18:2 w6,9c	
----	1961	---	----	----	Summed Feature 6	0.36	19:1 w11c/19:1 w9c	19:1 w9c/19:1 w11c	
----	43289	---	----	----	Summed Feature 8	7.82	18:1 w7c	18:1 w6c	
----	39382	---	----	----	Summed Feature 9	7.11	17:1 iso w9c	16:0 10-methyl	

ECL Deviation: 0.004                            Reference ECL Shift: 0.005      Number Reference Peaks: 12
Total Response: 802742                         Total Named: 551951
Percent Named: 68.76%                         Total Amount: 525746
Profile Comment:   Percent named is less than 85.00.

*** No Matches found in TSBA6
