# Supplementary material for: A green garlic (Allium sativum L.) based intercropping system reduces the strain of continuous monocropping in cucumber (Cucumis sativus L.) by adjusting the micro-ecological environment of soil
Source: PeerJ. 2019 Jul 15;7:e7267. doi: 10.7717/peerj.7267 (PMC6637937; doi:10.7717/peerj.7267)
Supplement: Data S1 [file peerj-07-7267-s001.zip › supplemental_Data_S1/45 days after interplanted/CB-1.rtf]

Volume: DATA            File: E131084.29A        Samp Ctr: 10                ID Number: 1003 
Type: Samp                   Bottle: 4                        Method: TSBA6 
Created: 1/8/2013 1:54:45 PM 
Sample ID: 48 


RT	Response	Ar/Ht	RFact	ECL	Peak Name	Percent	Comment1	Comment2	
1.645	4.559E+8	0.029	----	7.010	SOLVENT PEAK	----	< min rt		
1.777	1784	0.014	----	7.270		----	< min rt		
2.569	862	0.021	----	8.823		----	< min rt		
2.789	433	0.031	----	9.255		----			
3.035	2317	0.025	----	9.739		----			
3.347	686	0.034	----	10.256		----			
3.683	1217	0.036	----	10.735		----			
4.123	362	0.041	----	11.280		----			
4.209	798	0.031	----	11.372		----			
4.407	961	0.055	----	11.585		----			
4.549	700	0.029	----	11.738		----			
4.907	2084	0.032	1.021	12.101	11:0 iso 3OH	0.56	ECL deviates  0.012		
5.313	394	0.027	----	12.451		----			
5.501	913	0.050	1.003	12.612	13:0 iso	0.24	ECL deviates -0.002	Reference -0.008	
5.642	673	0.036	----	12.734		----			
5.799	732	0.031	----	12.869		----			
6.405	530	0.030	----	13.330		----			
6.456	1603	0.042	----	13.367		----			
6.803	2247	0.039	0.976	13.619	14:0 iso	0.58	ECL deviates  0.000	Reference -0.004	
6.962	495	0.037	----	13.735		----			
7.194	232	0.027	0.970	13.903	14:1 w5c	0.06	ECL deviates  0.002		
7.329	2458	0.036	0.969	14.001	14:0	0.63	ECL deviates  0.001	Reference -0.003	
7.782	5303	0.055	----	14.294		----			
8.009	1201	0.038	0.962	14.441	15:1 iso G	0.30	ECL deviates  0.001		
8.292	19570	0.038	0.959	14.624	15:0 iso	4.94	ECL deviates  0.001	Reference -0.003	
8.433	11292	0.041	0.958	14.715	15:0 anteiso	2.85	ECL deviates  0.002	Reference -0.002	
8.622	766	0.040	----	14.837		----			
8.876	1913	0.039	0.955	15.001	15:0	----	ECL deviates  0.001		
8.966	899	0.037	----	15.055		----			
9.376	773	0.038	----	15.300		----			
9.480	2346	0.049	----	15.363		----			
9.610	3507	0.059	0.951	15.441	16:1 iso G	0.88	ECL deviates -0.001		
9.921	10676	0.045	0.949	15.627	16:0 iso	2.67	ECL deviates  0.000	Reference -0.003	
10.082	1005	0.041	0.949	15.723	16:0 anteiso	0.25	ECL deviates  0.005		
10.161	3423	0.046	0.949	15.770	16:1 w9c	0.85	ECL deviates -0.004		
10.239	32065	0.043	0.948	15.817	Sum In Feature 3	8.00	ECL deviates -0.005	16:1 w7c/16:1 w6c	
10.390	9576	0.044	0.948	15.908	16:1 w5c	2.39	ECL deviates -0.001		
10.543	41429	0.043	0.947	15.999	16:0	10.33	ECL deviates -0.001	Reference -0.003	
10.631	702	0.043	----	16.050		----			
10.881	461	0.042	----	16.195		----			
11.099	231269	0.059	----	16.320		----			
11.290	71788	0.088	0.946	16.431	Sum In Feature 9	17.87	ECL deviates -0.001	16:0 10-methyl	
11.442	18714	0.078	0.946	16.518	17:1 anteiso w9c	4.66	ECL deviates -0.006		
11.635	19559	0.065	0.946	16.630	17:0 iso	4.87	ECL deviates  0.000	Reference -0.002	
11.797	15867	0.064	0.945	16.723	17:0 anteiso	3.95	ECL deviates  0.000	Reference -0.002	
11.921	8026	0.073	0.945	16.795	17:1 w8c	2.00	ECL deviates  0.003		
12.084	13305	0.063	0.945	16.889	17:0 cyclo	3.31	ECL deviates  0.001		
12.274	2498	0.048	0.945	16.999	17:0	0.62	ECL deviates -0.001	Reference -0.004	
12.346	5384	0.048	0.945	17.039	16:1 2OH	1.34	ECL deviates -0.009		
12.898	1624	0.040	----	17.353		----			
12.992	2180	0.044	0.945	17.406	17:0 10-methyl	0.54	ECL deviates -0.003		
13.145	2067	0.061	----	17.493		----			
13.545	6649	0.046	0.946	17.720	Sum In Feature 5	1.65	ECL deviates  0.000	18:2 w6,9c/18:0 ante	
13.633	23712	0.053	0.946	17.770	18:1 w9c	5.90	ECL deviates  0.001		
13.725	28402	0.047	0.946	17.822	Sum In Feature 8	7.07	ECL deviates -0.001	18:1 w7c	
13.876	4006	0.058	0.946	17.908	18:1 w5c	1.00	ECL deviates -0.011		
14.037	8611	0.044	0.946	18.000	18:0	2.14	ECL deviates  0.000	Reference -0.003	
14.179	2383	0.043	0.946	18.081	18:1 w7c 11-methyl	0.59	ECL deviates  0.000		
14.604	36916	0.067	----	18.323		----			
14.726	23948	0.085	0.947	18.393	18:0 10-methyl, TBSA	----	> max ar/ht		
15.015	395	0.024	----	18.558		----			
15.343	1183	0.043	----	18.745		----			
15.619	22591	0.051	0.948	18.903	19:0 cyclo w8c	5.63	ECL deviates  0.001		
15.848	138909	0.076	----	19.034		----			
15.885	166850	0.088	----	19.056		----	> max ar/ht		
16.379	1277	0.045	----	19.342		----			
16.476	2620	0.050	0.949	19.398	20:4 w6,9,12,15c	0.65	ECL deviates  0.003		
16.612	1144	0.052	----	19.476		----			
17.115	1549	0.039	0.949	19.768	20:1 w9c	0.39	ECL deviates -0.002		
17.518	1188	0.049	0.950	20.001	20:0	0.30	ECL deviates  0.001	Reference -0.003	
17.849	1131	0.049	----	20.193		----	> max rt		
18.174	2092	0.080	----	20.380		----	> max rt		
18.487	771	0.033	----	20.562		----	> max rt		
----	32065	---	----	----	Summed Feature 3	8.00	16:1 w7c/16:1 w6c	16:1 w6c/16:1 w7c	
----	6649	---	----	----	Summed Feature 5	1.65	18:2 w6,9c/18:0 ante	18:0 ante/18:2 w6,9c	
----	28402	---	----	----	Summed Feature 8	7.07	18:1 w7c	18:1 w6c	
----	71788	---	----	----	Summed Feature 9	17.87	17:1 iso w9c	16:0 10-methyl	

ECL Deviation: 0.004                            Reference ECL Shift: 0.004      Number Reference Peaks: 12
Total Response: 1029446                       Total Named: 400710
Percent Named: 38.92%                         Total Amount: 404524
Profile Comment:   Percent named is less than 85.00.

*** Library match not attempted
