# Supplementary material for: A green garlic (Allium sativum L.) based intercropping system reduces the strain of continuous monocropping in cucumber (Cucumis sativus L.) by adjusting the micro-ecological environment of soil
Source: PeerJ. 2019 Jul 15;7:e7267. doi: 10.7717/peerj.7267 (PMC6637937; doi:10.7717/peerj.7267)
Supplement: Data S1 [file peerj-07-7267-s001.zip › supplemental_Data_S1/45 days after interplanted/CB-2.rtf]

Volume: DATA            File: E131084.29A        Samp Ctr: 11                ID Number: 1004 
Type: Samp                   Bottle: 5                        Method: TSBA6 
Created: 1/8/2013 2:19:09 PM 
Sample ID: 49 


RT	Response	Ar/Ht	RFact	ECL	Peak Name	Percent	Comment1	Comment2	
1.645	4.516E+8	0.029	----	7.010	SOLVENT PEAK	----	< min rt		
1.778	1080	0.012	----	7.269		----	< min rt		
2.792	250	0.022	----	9.260		----			
3.059	339	0.027	----	9.785		----			
4.408	400	0.038	----	11.588		----			
4.907	1640	0.033	1.021	12.102	11:0 iso 3OH	0.64	ECL deviates  0.013		
5.499	285	0.024	1.003	12.611	13:0 iso	0.11	ECL deviates -0.003	Reference -0.010	
6.405	400	0.039	----	13.331		----			
6.805	1453	0.036	0.976	13.621	14:0 iso	0.54	ECL deviates  0.002	Reference -0.003	
7.327	1737	0.030	0.969	14.000	14:0	0.64	ECL deviates  0.000	Reference -0.004	
7.779	3631	0.053	----	14.292		----			
8.009	1131	0.044	0.962	14.441	15:1 iso G	0.41	ECL deviates  0.001		
8.293	15330	0.039	0.959	14.625	15:0 iso	5.58	ECL deviates  0.002	Reference -0.002	
8.432	8862	0.039	0.958	14.715	15:0 anteiso	3.22	ECL deviates  0.002	Reference -0.002	
8.877	1625	0.040	0.955	15.002	15:0	----	ECL deviates  0.002		
8.962	793	0.035	----	15.053		----			
9.615	1953	0.056	0.951	15.444	16:1 iso G	0.71	ECL deviates  0.002		
9.921	7528	0.039	0.949	15.627	16:0 iso	2.71	ECL deviates  0.000	Reference -0.003	
10.156	2914	0.053	0.949	15.768	16:1 w9c	1.05	ECL deviates -0.006		
10.238	25919	0.042	0.948	15.817	Sum In Feature 3	9.33	ECL deviates -0.005	16:1 w7c/16:1 w6c	
10.390	7803	0.042	0.948	15.908	16:1 w5c	2.81	ECL deviates -0.001		
10.543	36261	0.042	0.947	15.999	16:0	13.05	ECL deviates -0.001	Reference -0.003	
11.095	206022	0.058	----	16.318		----			
11.289	54175	0.082	0.946	16.430	Sum In Feature 9	19.46	ECL deviates -0.002	16:0 10-methyl	
11.633	5941	0.040	0.946	16.629	17:0 iso	2.13	ECL deviates -0.001	Reference -0.004	
11.796	5969	0.044	0.945	16.723	17:0 anteiso	2.14	ECL deviates  0.000	Reference -0.003	
11.917	1464	0.049	0.945	16.793	17:1 w8c	0.53	ECL deviates  0.001		
12.084	6661	0.048	0.945	16.889	17:0 cyclo	2.39	ECL deviates  0.001		
12.276	1581	0.046	0.945	17.000	17:0	0.57	ECL deviates  0.000	Reference -0.002	
12.342	3361	0.045	----	17.037		----			
12.989	1621	0.042	0.945	17.405	17:0 10-methyl	0.58	ECL deviates -0.004		
13.139	1208	0.051	----	17.490		----			
13.545	5658	0.046	0.946	17.721	Sum In Feature 5	2.03	ECL deviates  0.001	18:2 w6,9c/18:0 ante	
13.629	18816	0.049	0.946	17.769	18:1 w9c	6.76	ECL deviates  0.000		
13.724	24753	0.048	0.946	17.823	Sum In Feature 8	8.89	ECL deviates  0.000	18:1 w7c	
13.879	3045	0.053	0.946	17.911	18:1 w5c	1.09	ECL deviates -0.008		
14.034	8129	0.046	0.946	17.999	18:0	2.92	ECL deviates -0.001	Reference -0.004	
14.178	2543	0.055	0.946	18.081	18:1 w7c 11-methyl	0.91	ECL deviates  0.000		
14.604	34436	0.063	----	18.325		----			
14.724	21977	0.092	0.947	18.393	18:0 10-methyl, TBSA	----	> max ar/ht		
15.340	1074	0.047	----	18.745		----			
15.617	19442	0.048	0.948	18.904	19:0 cyclo w8c	7.00	ECL deviates  0.002		
15.898	340908	0.154	----	19.065		----	> max ar/ht		
16.477	1996	0.047	0.949	19.400	20:4 w6,9,12,15c	0.72	ECL deviates  0.005		
16.606	663	0.035	----	19.475		----			
17.118	1655	0.047	0.949	19.772	20:1 w9c	0.60	ECL deviates  0.002		
17.514	1331	0.050	0.950	20.001	20:0	0.48	ECL deviates  0.001	Reference -0.005	
17.847	1216	0.049	----	20.194		----	> max rt		
----	25919	---	----	----	Summed Feature 3	9.33	16:1 w7c/16:1 w6c	16:1 w6c/16:1 w7c	
----	5658	---	----	----	Summed Feature 5	2.03	18:2 w6,9c/18:0 ante	18:0 ante/18:2 w6,9c	
----	24753	---	----	----	Summed Feature 8	8.89	18:1 w7c	18:1 w6c	
----	54175	---	----	----	Summed Feature 9	19.46	17:1 iso w9c	16:0 10-methyl	

ECL Deviation: 0.004                            Reference ECL Shift: 0.004      Number Reference Peaks: 12
Total Response: 893054                         Total Named: 277593
Percent Named: 31.08%                         Total Amount: 285695
Profile Comment:   Percent named is less than 85.00.

*** Library match not attempted
