# Supplementary material for: A green garlic (Allium sativum L.) based intercropping system reduces the strain of continuous monocropping in cucumber (Cucumis sativus L.) by adjusting the micro-ecological environment of soil
Source: PeerJ. 2019 Jul 15;7:e7267. doi: 10.7717/peerj.7267 (PMC6637937; doi:10.7717/peerj.7267)
Supplement: Data S1 [file peerj-07-7267-s001.zip › supplemental_Data_S1/45 days after interplanted/CB-3.rtf]

Volume: DATA            File: E131084.29A        Samp Ctr: 16                ID Number: 1001 
Type: Samp                   Bottle: 2                        Method: TSBA6 
Created: 1/8/2013 4:20:19 PM 
Sample ID: 50 


RT	Response	Ar/Ht	RFact	ECL	Peak Name	Percent	Comment1	Comment2	
1.646	4.558E+8	0.028	----	7.008	SOLVENT PEAK	----	< min rt		
1.778	4702	0.023	----	7.267		----	< min rt		
2.287	225	0.022	----	8.266		----	< min rt		
2.791	459	0.030	----	9.256		----			
3.060	299	0.022	----	9.784		----			
3.351	398	0.026	----	10.269		----			
4.119	300	0.030	----	11.275		----			
4.791	627	0.043	1.025	11.998	12:0	0.22	ECL deviates -0.002	Reference -0.009	
4.908	1861	0.033	1.021	12.100	11:0 iso 3OH	0.65	ECL deviates  0.011		
6.402	551	0.040	----	13.327		----			
6.806	1518	0.034	0.975	13.620	14:0 iso	0.51	ECL deviates  0.001	Reference -0.002	
7.331	2199	0.035	0.967	14.002	14:0	0.73	ECL deviates  0.002	Reference -0.002	
7.781	4037	0.050	----	14.292		----			
8.009	870	0.035	0.960	14.440	15:1 iso G	0.29	ECL deviates  0.000		
8.294	16552	0.038	0.958	14.624	15:0 iso	5.45	ECL deviates  0.001	Reference -0.002	
8.434	9468	0.040	0.957	14.715	15:0 anteiso	3.11	ECL deviates  0.002	Reference -0.001	
8.878	1573	0.037	0.953	15.002	15:0	----	ECL deviates  0.002		
8.963	681	0.032	----	15.053		----			
9.381	983	0.044	----	15.303		----			
9.617	2694	0.072	0.949	15.444	16:1 iso G	0.88	ECL deviates  0.002		
9.922	8345	0.041	0.948	15.627	16:0 iso	2.72	ECL deviates  0.000	Reference -0.002	
10.078	467	0.033	0.948	15.720	16:0 anteiso	0.15	ECL deviates  0.002		
10.158	2796	0.049	0.947	15.768	16:1 w9c	0.91	ECL deviates -0.006		
10.241	27102	0.043	0.947	15.818	Sum In Feature 3	8.82	ECL deviates -0.004	16:1 w7c/16:1 w6c	
10.392	7825	0.040	0.947	15.908	16:1 w5c	2.55	ECL deviates -0.001		
10.544	37369	0.042	0.946	15.999	16:0	12.16	ECL deviates -0.001	Reference -0.003	
11.093	176918	0.056	----	16.316		----			
11.290	55608	0.090	0.945	16.430	Sum In Feature 9	18.06	ECL deviates -0.002	16:0 10-methyl	
11.634	6092	0.040	0.944	16.629	17:0 iso	1.98	ECL deviates -0.001	Reference -0.003	
11.795	6112	0.045	0.944	16.722	17:0 anteiso	1.98	ECL deviates -0.001	Reference -0.003	
11.919	1694	0.041	0.944	16.793	17:1 w8c	0.55	ECL deviates  0.001		
12.086	6647	0.048	0.944	16.889	17:0 cyclo	2.16	ECL deviates  0.001		
12.279	1849	0.050	0.944	17.001	17:0	0.60	ECL deviates  0.001	Reference -0.001	
12.347	3831	0.044	0.944	17.039	16:1 2OH	1.24	ECL deviates -0.009		
12.997	1550	0.042	0.944	17.408	17:0 10-methyl	0.50	ECL deviates -0.001		
13.146	557	0.035	----	17.493		----			
13.549	5244	0.045	0.945	17.722	Sum In Feature 5	1.70	ECL deviates  0.002	18:2 w6,9c/18:0 ante	
13.633	19004	0.051	0.945	17.770	18:1 w9c	6.17	ECL deviates  0.001		
13.725	24064	0.047	0.945	17.822	Sum In Feature 8	7.81	ECL deviates -0.001	18:1 w7c	
13.876	3186	0.057	0.945	17.908	18:1 w5c	1.03	ECL deviates -0.011		
14.036	8899	0.044	0.945	17.998	18:0	2.89	ECL deviates -0.002	Reference -0.005	
14.179	2298	0.050	0.945	18.080	18:1 w7c 11-methyl	0.75	ECL deviates -0.001		
14.603	25051	0.063	----	18.323		----			
14.724	17298	0.078	0.946	18.392	18:0 10-methyl, TBSA	5.62	ECL deviates  0.000		
15.004	541	0.045	----	18.552		----			
15.206	706	0.041	----	18.668		----			
15.346	1135	0.043	0.946	18.748	Sum In Feature 6	0.37	ECL deviates -0.008	19:1 w11c/19:1 w9c	
15.620	16735	0.045	0.947	18.905	19:0 cyclo w8c	5.45	ECL deviates  0.003		
16.480	2237	0.046	0.947	19.402	20:4 w6,9,12,15c	0.73	ECL deviates  0.007		
16.614	671	0.047	----	19.479		----			
17.122	2700	0.070	0.948	19.773	20:1 w9c	0.88	ECL deviates  0.003		
17.517	1120	0.044	0.948	20.001	20:0	0.36	ECL deviates  0.001	Reference -0.005	
17.852	1231	0.048	----	20.195		----	> max rt		
----	27102	---	----	----	Summed Feature 3	8.82	16:1 w7c/16:1 w6c	16:1 w6c/16:1 w7c	
----	5244	---	----	----	Summed Feature 5	1.70	18:2 w6,9c/18:0 ante	18:0 ante/18:2 w6,9c	
----	1135	---	----	----	Summed Feature 6	0.37	19:1 w11c/19:1 w9c	19:1 w9c/19:1 w11c	
----	24064	---	----	----	Summed Feature 8	7.81	18:1 w7c	18:1 w6c	
----	55608	---	----	----	Summed Feature 9	18.06	17:1 iso w9c	16:0 10-methyl	

ECL Deviation: 0.004                            Reference ECL Shift: 0.004      Number Reference Peaks: 12
Total Response: 519152                         Total Named: 306998
Percent Named: 59.13%                         Total Amount: 292402
Profile Comment:   Percent named is less than 85.00.

*** No Matches found in TSBA6
