# Supplementary material for: A green garlic (Allium sativum L.) based intercropping system reduces the strain of continuous monocropping in cucumber (Cucumis sativus L.) by adjusting the micro-ecological environment of soil
Source: PeerJ. 2019 Jul 15;7:e7267. doi: 10.7717/peerj.7267 (PMC6637937; doi:10.7717/peerj.7267)
Supplement: Data S1 [file peerj-07-7267-s001.zip › supplemental_Data_S1/45 days after interplanted/CR-1.rtf]

Volume: DATA            File: E131094.42A        Samp Ctr: 5                  ID Number: 1003 
Type: Samp                   Bottle: 4                        Method: TSBA6 
Created: 1/9/2013 12:11:58 PM 
Sample ID: 62 


RT	Response	Ar/Ht	RFact	ECL	Peak Name	Percent	Comment1	Comment2	
1.645	4.525E+8	0.029	----	7.011	SOLVENT PEAK	----	< min rt		
1.778	6597	0.024	----	7.271		----	< min rt		
1.835	276	0.025	----	7.383		----	< min rt		
2.035	447	0.028	----	7.776		----	< min rt		
2.153	209	0.022	----	8.007		----	< min rt		
3.061	554	0.025	----	9.789		----			
3.350	371	0.026	----	10.261		----			
4.403	657	0.033	----	11.581		----			
4.905	2417	0.035	1.017	12.099	11:0 iso 3OH	1.00	ECL deviates  0.010		
5.111	3702	0.035	----	12.276		----			
5.501	273	0.031	0.998	12.612	13:0 iso	0.11	ECL deviates -0.002	Reference -0.007	
6.804	1342	0.035	0.972	13.620	14:0 iso	0.53	ECL deviates  0.001	Reference -0.003	
7.328	1848	0.035	0.965	14.000	14:0	0.73	ECL deviates  0.000	Reference -0.003	
7.775	9089	0.045	----	14.290		----			
8.006	820	0.038	0.958	14.439	15:1 iso G	0.32	ECL deviates -0.001		
8.292	12626	0.038	0.956	14.624	15:0 iso	4.92	ECL deviates  0.001	Reference -0.002	
8.432	7566	0.038	0.955	14.714	15:0 anteiso	2.95	ECL deviates  0.001	Reference -0.001	
8.874	1348	0.038	0.952	15.001	15:0	----	ECL deviates  0.001		
8.964	485	0.031	----	15.054		----			
9.613	1838	0.060	0.949	15.443	16:1 iso G	0.71	ECL deviates  0.001		
9.920	6122	0.041	0.948	15.626	16:0 iso	2.37	ECL deviates -0.001	Reference -0.002	
10.155	2429	0.044	0.948	15.767	16:1 w9c	0.94	ECL deviates -0.007		
10.239	24950	0.042	0.947	15.817	Sum In Feature 3	9.64	ECL deviates -0.005	16:1 w7c/16:1 w6c	
10.391	6231	0.043	0.947	15.908	16:1 w5c	2.41	ECL deviates -0.001		
10.543	31469	0.042	0.947	15.999	16:0	12.15	ECL deviates -0.001	Reference -0.002	
11.088	146261	0.057	----	16.314		----			
11.290	43185	0.087	0.946	16.431	Sum In Feature 9	16.66	ECL deviates -0.001	16:0 10-methyl	
11.440	12374	0.085	0.946	16.517	17:1 anteiso w9c	----	> max ar/ht		
11.637	11311	0.060	0.946	16.631	17:0 iso	4.36	ECL deviates  0.001	Reference  0.000	
11.797	9601	0.059	0.946	16.724	17:0 anteiso	3.70	ECL deviates  0.001	Reference -0.001	
11.918	4974	0.070	0.946	16.793	17:1 w8c	1.92	ECL deviates  0.001		
12.085	7996	0.053	0.946	16.890	17:0 cyclo	3.08	ECL deviates  0.002		
12.276	2443	0.059	0.946	17.000	17:0	0.94	ECL deviates  0.000	Reference -0.001	
12.345	3046	0.048	0.946	17.039	16:1 2OH	1.18	ECL deviates -0.009		
12.996	1305	0.039	0.947	17.408	17:0 10-methyl	0.50	ECL deviates -0.001		
13.140	883	0.046	----	17.489		----			
13.546	4937	0.044	0.948	17.720	Sum In Feature 5	1.91	ECL deviates  0.000	18:2 w6,9c/18:0 ante	
13.633	15468	0.048	0.948	17.769	18:1 w9c	5.98	ECL deviates  0.000		
13.725	22789	0.050	0.948	17.821	Sum In Feature 8	8.81	ECL deviates -0.002	18:1 w7c	
13.884	2358	0.049	0.948	17.911	18:1 w5c	0.91	ECL deviates -0.008		
14.035	6752	0.045	0.948	17.997	18:0	2.61	ECL deviates -0.003	Reference -0.005	
14.179	2115	0.046	0.949	18.079	18:1 w7c 11-methyl	0.82	ECL deviates -0.002		
14.607	43163	0.064	----	18.324		----			
14.727	23265	0.088	0.949	18.393	18:0 10-methyl, TBSA	----	> max ar/ht		
15.621	16738	0.050	0.951	18.904	19:0 cyclo w8c	6.49	ECL deviates  0.002		
15.888	313939	0.154	----	19.058		----	> max ar/ht		
16.479	1430	0.046	0.952	19.400	20:4 w6,9,12,15c	0.55	ECL deviates  0.005		
16.595	491	0.039	----	19.466		----			
17.117	1119	0.041	0.952	19.768	20:1 w9c	0.43	ECL deviates -0.002		
17.520	977	0.046	0.952	20.001	20:0	0.38	ECL deviates  0.001	Reference -0.002	
17.851	937	0.038	----	20.192		----	> max rt		
----	24950	---	----	----	Summed Feature 3	9.64	16:1 w7c/16:1 w6c	16:1 w6c/16:1 w7c	
----	4937	---	----	----	Summed Feature 5	1.91	18:2 w6,9c/18:0 ante	18:0 ante/18:2 w6,9c	
----	22789	---	----	----	Summed Feature 8	8.81	18:1 w7c	18:1 w6c	
----	43185	---	----	----	Summed Feature 9	16.66	17:1 iso w9c	16:0 10-methyl	

ECL Deviation: 0.004                            Reference ECL Shift: 0.003      Number Reference Peaks: 12
Total Response: 813707                         Total Named: 258474
Percent Named: 31.77%                         Total Amount: 280360
Profile Comment:   Percent named is less than 85.00.

*** Library match not attempted
