# Supplementary material for: A green garlic (Allium sativum L.) based intercropping system reduces the strain of continuous monocropping in cucumber (Cucumis sativus L.) by adjusting the micro-ecological environment of soil
Source: PeerJ. 2019 Jul 15;7:e7267. doi: 10.7717/peerj.7267 (PMC6637937; doi:10.7717/peerj.7267)
Supplement: Data S1 [file peerj-07-7267-s001.zip › supplemental_Data_S1/45 days after interplanted/CR-2.rtf]

Volume: DATA            File: E131094.42A        Samp Ctr: 6                  ID Number: 1004 
Type: Samp                   Bottle: 5                        Method: TSBA6 
Created: 1/9/2013 12:36:06 PM 
Sample ID: 63 


RT	Response	Ar/Ht	RFact	ECL	Peak Name	Percent	Comment1	Comment2	
1.646	4.538E+8	0.029	----	7.008	SOLVENT PEAK	----	< min rt		
1.778	1039	0.010	----	7.269		----	< min rt		
2.031	299	0.025	----	7.765		----	< min rt		
2.790	344	0.024	----	9.255		----			
3.059	195	0.018	----	9.785		----			
3.353	556	0.029	----	10.264		----			
4.408	282	0.030	----	11.584		----			
4.776	691	0.034	----	11.981		----			
4.906	1713	0.031	1.017	12.098	11:0 iso 3OH	0.68	ECL deviates  0.009		
5.114	2498	0.036	----	12.277		----			
5.503	272	0.025	0.998	12.612	13:0 iso	0.11	ECL deviates -0.002	Reference -0.006	
6.805	1504	0.034	0.972	13.619	14:0 iso	0.57	ECL deviates  0.000	Reference -0.003	
7.331	1925	0.038	0.965	14.001	14:0	0.72	ECL deviates  0.001	Reference -0.001	
7.780	6294	0.049	----	14.292		----			
8.009	812	0.036	0.958	14.440	15:1 iso G	0.30	ECL deviates  0.000		
8.292	14512	0.037	0.956	14.624	15:0 iso	5.41	ECL deviates  0.001	Reference -0.002	
8.432	8673	0.039	0.955	14.714	15:0 anteiso	3.23	ECL deviates  0.001	Reference -0.001	
8.876	1573	0.036	0.952	15.001	15:0	----	ECL deviates  0.001		
8.966	692	0.036	----	15.055		----			
9.628	1721	0.064	0.949	15.452	16:1 iso H	0.64	ECL deviates -0.009		
9.920	7214	0.040	0.948	15.627	16:0 iso	2.67	ECL deviates  0.000	Reference -0.002	
10.156	2983	0.044	0.948	15.767	16:1 w9c	1.10	ECL deviates -0.007		
10.239	28898	0.044	0.947	15.817	Sum In Feature 3	10.68	ECL deviates -0.005	16:1 w7c/16:1 w6c	
10.390	7412	0.040	0.947	15.908	16:1 w5c	2.74	ECL deviates -0.001		
10.541	35466	0.043	0.947	15.998	16:0	13.10	ECL deviates -0.002	Reference -0.003	
11.081	81243	0.057	----	16.310		----			
11.288	36045	0.077	0.946	16.430	Sum In Feature 9	13.30	ECL deviates -0.002	16:0 10-methyl	
11.635	5456	0.040	0.946	16.630	17:0 iso	2.01	ECL deviates  0.000	Reference -0.001	
11.796	5679	0.044	0.946	16.724	17:0 anteiso	2.10	ECL deviates  0.001	Reference -0.001	
11.918	1952	0.042	0.946	16.794	17:1 w8c	0.72	ECL deviates  0.002		
12.083	6707	0.046	0.946	16.889	17:0 cyclo	2.47	ECL deviates  0.001		
12.276	2016	0.053	0.946	17.001	17:0	0.74	ECL deviates  0.001	Reference -0.001	
12.343	3023	0.046	0.946	17.039	16:1 2OH	1.12	ECL deviates -0.009		
12.995	1303	0.039	0.947	17.408	17:0 10-methyl	0.48	ECL deviates -0.001		
13.148	592	0.032	----	17.495		----			
13.546	5857	0.045	0.948	17.721	Sum In Feature 5	2.16	ECL deviates  0.001	18:2 w6,9c/18:0 ante	
13.631	17430	0.050	0.948	17.769	18:1 w9c	6.44	ECL deviates  0.000		
13.725	25434	0.049	0.948	17.822	Sum In Feature 8	9.40	ECL deviates -0.001	18:1 w7c	
13.881	3030	0.061	0.948	17.910	18:1 w5c	1.12	ECL deviates -0.009		
14.035	7641	0.046	0.948	17.997	18:0	2.83	ECL deviates -0.003	Reference -0.005	
14.177	2125	0.047	0.949	18.079	18:1 w7c 11-methyl	0.79	ECL deviates -0.002		
14.603	19441	0.063	----	18.323		----			
14.723	10544	0.061	0.949	18.391	18:0 10-methyl, TBSA	3.90	ECL deviates -0.001		
14.786	4911	0.049	----	18.427		----			
15.016	317	0.035	----	18.559		----			
15.339	846	0.048	----	18.744		----			
15.620	17871	0.051	0.951	18.905	19:0 cyclo w8c	6.63	ECL deviates  0.003		
15.892	247420	0.148	----	19.061		----	> max ar/ht		
16.478	1997	0.045	0.952	19.399	20:4 w6,9,12,15c	0.74	ECL deviates  0.004		
16.610	683	0.039	----	19.476		----			
16.753	283	0.029	0.952	19.559	18:0 3OH	0.10	ECL deviates  0.009		
17.122	1557	0.056	0.952	19.772	20:1 w9c	0.58	ECL deviates  0.002		
17.518	1123	0.044	0.952	20.001	20:0	0.42	ECL deviates  0.001	Reference -0.003	
17.852	1022	0.038	----	20.194		----	> max rt		
----	28898	---	----	----	Summed Feature 3	10.68	16:1 w7c/16:1 w6c	16:1 w6c/16:1 w7c	
----	5857	---	----	----	Summed Feature 5	2.16	18:2 w6,9c/18:0 ante	18:0 ante/18:2 w6,9c	
----	25434	---	----	----	Summed Feature 8	9.40	18:1 w7c	18:1 w6c	
----	36045	---	----	----	Summed Feature 9	13.30	17:1 iso w9c	16:0 10-methyl	

ECL Deviation: 0.004                            Reference ECL Shift: 0.003      Number Reference Peaks: 12
Total Response: 637182                         Total Named: 270176
Percent Named: 42.40%                         Total Amount: 257909
Profile Comment:   Percent named is less than 85.00.

*** Library match not attempted
