# Supplementary material for: A green garlic (Allium sativum L.) based intercropping system reduces the strain of continuous monocropping in cucumber (Cucumis sativus L.) by adjusting the micro-ecological environment of soil
Source: PeerJ. 2019 Jul 15;7:e7267. doi: 10.7717/peerj.7267 (PMC6637937; doi:10.7717/peerj.7267)
Supplement: Data S1 [file peerj-07-7267-s001.zip › supplemental_Data_S1/45 days after interplanted/CR-3.rtf]

Volume: DATA            File: E131094.42A        Samp Ctr: 8                  ID Number: 1011 
Type: Samp                   Bottle: 6                        Method: TSBA6 
Created: 1/9/2013 1:24:43 PM 
Sample ID: 64 


RT	Response	Ar/Ht	RFact	ECL	Peak Name	Percent	Comment1	Comment2	
1.646	4.516E+8	0.029	----	7.008	SOLVENT PEAK	----	< min rt		
1.778	2831	0.024	----	7.267		----	< min rt		
4.904	488	0.031	1.017	12.091	11:0 iso 3OH	0.19	ECL deviates  0.002		
6.807	1288	0.033	0.972	13.619	14:0 iso	0.49	ECL deviates  0.000	Reference -0.001	
7.328	1757	0.039	0.965	13.998	14:0	0.66	ECL deviates -0.002	Reference -0.003	
7.806	2484	0.055	----	14.308		----			
8.009	1454	0.053	0.958	14.439	15:1 iso G	0.54	ECL deviates -0.001		
8.293	14022	0.037	0.956	14.624	15:0 iso	5.20	ECL deviates  0.001	Reference -0.001	
8.433	8336	0.039	0.955	14.714	15:0 anteiso	3.09	ECL deviates  0.001	Reference -0.001	
8.876	1803	0.041	0.952	15.001	15:0	----	ECL deviates  0.001		
8.966	777	0.034	----	15.055		----			
9.616	1946	0.055	0.949	15.445	16:1 iso G	0.72	ECL deviates  0.003		
9.922	7498	0.039	0.948	15.628	16:0 iso	2.76	ECL deviates  0.001	Reference -0.001	
10.160	2867	0.053	0.948	15.770	16:1 w9c	1.05	ECL deviates -0.004		
10.241	28179	0.043	0.947	15.819	Sum In Feature 3	10.35	ECL deviates -0.003	16:1 w7c/16:1 w6c	
10.392	7129	0.042	0.947	15.909	16:1 w5c	2.62	ECL deviates  0.000		
10.543	35883	0.041	0.947	16.000	16:0	13.18	ECL deviates  0.000	Reference -0.002	
10.631	499	0.041	----	16.051		----			
11.086	19192	0.074	----	16.314		----			
11.288	31949	0.079	0.946	16.430	Sum In Feature 9	11.72	ECL deviates -0.002	16:0 10-methyl	
11.449	5935	0.082	0.946	16.523	17:1 anteiso w9c	----	> max ar/ht		
11.635	7760	0.047	0.946	16.631	17:0 iso	2.85	ECL deviates  0.001	Reference -0.002	
11.797	7090	0.049	0.946	16.724	17:0 anteiso	2.60	ECL deviates  0.001	Reference -0.001	
11.919	2324	0.047	0.946	16.795	17:1 w8c	0.85	ECL deviates  0.003		
12.085	7281	0.052	0.946	16.891	17:0 cyclo	2.67	ECL deviates  0.003		
12.270	1430	0.037	0.946	16.997	17:0	0.52	ECL deviates -0.003	Reference -0.005	
12.346	3056	0.042	0.946	17.041	16:1 2OH	1.12	ECL deviates -0.007		
12.465	317	0.031	----	17.108		----			
12.995	1775	0.043	0.947	17.409	17:0 10-methyl	0.65	ECL deviates  0.000		
13.151	1217	0.045	----	17.496		----			
13.546	6352	0.045	0.948	17.720	Sum In Feature 5	2.33	ECL deviates  0.000	18:2 w6,9c/18:0 ante	
13.632	18436	0.051	0.948	17.769	18:1 w9c	6.78	ECL deviates  0.000		
13.727	26197	0.049	0.948	17.823	Sum In Feature 8	9.63	ECL deviates  0.000	18:1 w7c	
13.881	3158	0.055	0.948	17.910	18:1 w5c	1.16	ECL deviates -0.009		
14.035	7789	0.047	0.948	17.997	18:0	2.86	ECL deviates -0.003	Reference -0.005	
14.176	2867	0.052	0.949	18.078	18:1 w7c 11-methyl	1.05	ECL deviates -0.003		
14.726	8183	0.072	0.949	18.392	18:0 10-methyl, TBSA	3.01	ECL deviates  0.000		
14.784	2609	0.040	----	18.426		----			
15.351	1076	0.042	0.950	18.750	Sum In Feature 6	0.40	ECL deviates -0.006	19:1 w11c/19:1 w9c	
15.621	18590	0.050	0.951	18.904	19:0 cyclo w8c	6.85	ECL deviates  0.002		
15.899	290192	0.152	----	19.063		----	> max ar/ht		
16.478	1862	0.051	0.952	19.398	20:4 w6,9,12,15c	0.69	ECL deviates  0.003		
16.608	455	0.034	----	19.473		----			
16.902	1262	0.070	0.952	19.643	20:0 iso	0.47	ECL deviates  0.008	Reference  0.006	
17.115	1571	0.049	0.952	19.766	20:1 w9c	0.58	ECL deviates -0.004		
17.512	934	0.040	0.952	19.995	20:0	0.34	ECL deviates -0.005	Reference -0.006	
17.848	1170	0.044	----	20.188		----	> max rt		
18.172	1404	0.076	----	20.375		----	> max rt		
----	28179	---	----	----	Summed Feature 3	10.35	16:1 w7c/16:1 w6c	16:1 w6c/16:1 w7c	
----	6352	---	----	----	Summed Feature 5	2.33	18:2 w6,9c/18:0 ante	18:0 ante/18:2 w6,9c	
----	1076	---	----	----	Summed Feature 6	0.40	19:1 w11c/19:1 w9c	19:1 w9c/19:1 w11c	
----	26197	---	----	----	Summed Feature 8	9.63	18:1 w7c	18:1 w6c	
----	31949	---	----	----	Summed Feature 9	11.72	17:1 iso w9c	16:0 10-methyl	

ECL Deviation: 0.003                            Reference ECL Shift: 0.004      Number Reference Peaks: 12
Total Response: 595463                         Total Named: 271786
Percent Named: 45.64%                         Total Amount: 265167
Profile Comment:   Percent named is less than 85.00.

*** No Matches found in TSBA6
