# Supplementary material for: A green garlic (Allium sativum L.) based intercropping system reduces the strain of continuous monocropping in cucumber (Cucumis sativus L.) by adjusting the micro-ecological environment of soil
Source: PeerJ. 2019 Jul 15;7:e7267. doi: 10.7717/peerj.7267 (PMC6637937; doi:10.7717/peerj.7267)
Supplement: Data S1 [file peerj-07-7267-s001.zip › supplemental_Data_S1/45 days after interplanted/GB-1.rtf]

Volume: DATA            File: E131084.29A        Samp Ctr: 6                  ID Number: 1004 
Type: Samp                   Bottle: 5                        Method: TSBA6 
Created: 1/8/2013 12:17:47 PM 
Sample ID: 42 


RT	Response	Ar/Ht	RFact	ECL	Peak Name	Percent	Comment1	Comment2	
1.645	4.573E+8	0.029	----	7.011	SOLVENT PEAK	----	< min rt		
1.777	3307	0.023	----	7.269		----	< min rt		
1.834	202	0.021	----	7.382		----	< min rt		
1.925	244	0.032	----	7.560		----	< min rt		
3.060	568	0.026	----	9.788		----			
4.407	356	0.025	----	11.585		----			
4.907	1501	0.031	1.021	12.100	11:0 iso 3OH	0.63	ECL deviates  0.011		
5.115	1643	0.038	----	12.279		----			
5.502	257	0.026	1.003	12.613	13:0 iso	0.11	ECL deviates -0.001	Reference -0.007	
6.404	487	0.044	----	13.329		----			
6.805	1212	0.034	0.976	13.621	14:0 iso	0.48	ECL deviates  0.002	Reference -0.003	
7.330	1622	0.038	0.969	14.001	14:0	0.64	ECL deviates  0.001	Reference -0.002	
7.782	5267	0.052	----	14.294		----			
8.013	913	0.037	0.962	14.443	15:1 iso G	0.36	ECL deviates  0.003		
8.292	13358	0.037	0.959	14.623	15:0 iso	5.23	ECL deviates  0.000	Reference -0.003	
8.433	8134	0.040	0.958	14.714	15:0 anteiso	3.18	ECL deviates  0.001	Reference -0.001	
8.875	1377	0.038	0.955	15.000	15:0	----	ECL deviates  0.000		
8.964	827	0.041	----	15.054		----			
9.618	1954	0.056	0.951	15.445	16:1 iso G	0.76	ECL deviates  0.003		
9.921	7486	0.040	0.949	15.626	16:0 iso	2.90	ECL deviates -0.001	Reference -0.003	
10.158	2151	0.050	0.949	15.768	16:1 w9c	0.83	ECL deviates -0.006		
10.241	26275	0.044	0.948	15.818	Sum In Feature 3	10.17	ECL deviates -0.004	16:1 w7c/16:1 w6c	
10.391	6210	0.041	0.948	15.907	16:1 w5c	2.40	ECL deviates -0.002		
10.543	32722	0.042	0.947	15.998	16:0	12.65	ECL deviates -0.002	Reference -0.004	
10.634	487	0.038	----	16.051		----			
11.080	77016	0.058	----	16.309		----			
11.287	40059	0.081	0.946	16.428	Sum In Feature 9	15.46	ECL deviates -0.004	16:0 10-methyl	
11.450	8922	0.081	0.946	16.522	17:1 anteiso w9c	----	> max ar/ht		
11.633	10287	0.057	0.946	16.628	17:0 iso	3.97	ECL deviates -0.002	Reference -0.004	
11.796	8319	0.052	0.945	16.722	17:0 anteiso	3.21	ECL deviates -0.001	Reference -0.002	
11.916	3847	0.055	0.945	16.791	17:1 w8c	1.48	ECL deviates -0.001		
12.083	8269	0.052	0.945	16.887	17:0 cyclo	3.19	ECL deviates -0.001		
12.276	2144	0.057	0.945	16.999	17:0	0.83	ECL deviates -0.001	Reference -0.003	
12.344	3552	0.044	----	17.038		----			
12.993	1846	0.045	0.945	17.406	17:0 10-methyl	0.71	ECL deviates -0.003		
13.150	1541	0.055	----	17.495		----			
13.327	1154	0.081	----	17.595		----	> max ar/ht		
13.547	9169	0.046	0.946	17.720	Sum In Feature 5	3.54	ECL deviates  0.000	18:2 w6,9c/18:0 ante	
13.633	16309	0.049	0.946	17.769	18:1 w9c	6.29	ECL deviates  0.000		
13.725	22176	0.049	0.946	17.821	Sum In Feature 8	8.56	ECL deviates -0.002	18:1 w7c	
13.878	3452	0.059	0.946	17.908	18:1 w5c	1.33	ECL deviates -0.011		
14.035	6723	0.044	0.946	17.997	18:0	2.60	ECL deviates -0.003	Reference -0.004	
14.176	1966	0.040	0.946	18.078	18:1 w7c 11-methyl	0.76	ECL deviates -0.003		
14.604	21041	0.067	----	18.322		----			
14.726	16810	0.088	0.947	18.392	18:0 10-methyl, TBSA	----	> max ar/ht		
15.021	496	0.032	----	18.560		----			
15.339	850	0.044	----	18.742		----		Reference  0.010	
15.620	16831	0.052	0.948	18.903	19:0 cyclo w8c	6.51	ECL deviates  0.001		
15.868	265550	0.158	----	19.044		----	> max ar/ht		
16.476	1047	0.039	0.949	19.396	20:4 w6,9,12,15c	0.41	ECL deviates  0.001		
17.119	944	0.034	0.949	19.768	20:1 w9c	0.37	ECL deviates -0.002		
17.513	1176	0.052	0.950	19.996	20:0	0.46	ECL deviates -0.004	Reference -0.006	
17.850	987	0.039	----	20.191		----	> max rt		
18.179	1151	0.064	----	20.381		----	> max rt		
----	26275	---	----	----	Summed Feature 3	10.17	16:1 w7c/16:1 w6c	16:1 w6c/16:1 w7c	
----	9169	---	----	----	Summed Feature 5	3.54	18:2 w6,9c/18:0 ante	18:0 ante/18:2 w6,9c	
----	22176	---	----	----	Summed Feature 8	8.56	18:1 w7c	18:1 w6c	
----	40059	---	----	----	Summed Feature 9	15.46	17:1 iso w9c	16:0 10-methyl	

ECL Deviation: 0.004                            Reference ECL Shift: 0.004      Number Reference Peaks: 13
Total Response: 664927                         Total Named: 258359
Percent Named: 38.86%                         Total Amount: 270724
Profile Comment:   Percent named is less than 85.00.

*** Library match not attempted
