# Supplementary material for: A green garlic (Allium sativum L.) based intercropping system reduces the strain of continuous monocropping in cucumber (Cucumis sativus L.) by adjusting the micro-ecological environment of soil
Source: PeerJ. 2019 Jul 15;7:e7267. doi: 10.7717/peerj.7267 (PMC6637937; doi:10.7717/peerj.7267)
Supplement: Data S1 [file peerj-07-7267-s001.zip › supplemental_Data_S1/45 days after interplanted/GB-2.rtf]

Volume: DATA            File: E131084.29A        Samp Ctr: 7                  ID Number: 1008 
Type: Samp                   Bottle: 6                        Method: TSBA6 
Created: 1/8/2013 12:42:06 PM 
Sample ID: 43 


RT	Response	Ar/Ht	RFact	ECL	Peak Name	Percent	Comment1	Comment2	
1.646	4.553E+8	0.029	----	7.004	SOLVENT PEAK	----	< min rt		
1.777	3352	0.023	----	7.262		----	< min rt		
2.287	311	0.030	----	8.263		----	< min rt		
3.059	601	0.030	----	9.780		----			
4.405	505	0.030	----	11.578		----			
4.907	1272	0.028	1.021	12.096	11:0 iso 3OH	0.55	ECL deviates  0.007		
5.007	275	0.027	----	12.182		----			
5.124	704	0.033	----	12.283		----			
5.507	360	0.034	1.003	12.614	13:0 iso	0.15	ECL deviates  0.000	Reference -0.003	
6.406	567	0.032	----	13.328		----			
6.806	1013	0.031	0.976	13.619	14:0 iso	0.42	ECL deviates  0.000	Reference -0.002	
7.331	1385	0.035	0.969	14.000	14:0	0.57	ECL deviates  0.000	Reference -0.002	
7.796	2680	0.044	----	14.302		----			
8.011	726	0.036	0.962	14.441	15:1 iso G	0.30	ECL deviates  0.001		
8.293	12383	0.038	0.959	14.623	15:0 iso	5.03	ECL deviates  0.000	Reference -0.002	
8.434	7470	0.040	0.958	14.714	15:0 anteiso	3.03	ECL deviates  0.001	Reference -0.001	
8.813	430	0.030	0.955	14.960	unknown 14.959	----	ECL deviates  0.001		
8.877	1375	0.036	0.955	15.001	15:0	----	ECL deviates  0.001		
8.967	722	0.039	----	15.055		----			
9.624	1897	0.062	0.951	15.449	16:1 iso G	0.76	ECL deviates  0.007		
9.923	6968	0.041	0.949	15.628	16:0 iso	2.80	ECL deviates  0.001	Reference -0.002	
10.155	2109	0.046	0.949	15.767	16:1 w9c	0.85	ECL deviates -0.007		
10.240	24619	0.043	0.948	15.818	Sum In Feature 3	9.89	ECL deviates -0.004	16:1 w7c/16:1 w6c	
10.391	5899	0.041	0.948	15.908	16:1 w5c	2.37	ECL deviates -0.001		
10.543	31355	0.042	0.947	15.999	16:0	12.58	ECL deviates -0.001	Reference -0.003	
11.085	34553	0.069	----	16.312		----			
11.290	32121	0.074	0.946	16.431	Sum In Feature 9	12.87	ECL deviates -0.001	16:0 10-methyl	
11.433	7192	0.081	----	16.514		----	> max ar/ht		
11.634	7689	0.048	0.946	16.630	17:0 iso	3.08	ECL deviates  0.000	Reference -0.003	
11.797	6835	0.052	0.945	16.723	17:0 anteiso	2.74	ECL deviates  0.000	Reference -0.002	
11.918	2583	0.052	0.945	16.793	17:1 w8c	1.03	ECL deviates  0.001		
12.085	7201	0.050	0.945	16.890	17:0 cyclo	2.88	ECL deviates  0.002		
12.275	1664	0.044	0.945	17.000	17:0	0.67	ECL deviates  0.000	Reference -0.003	
12.345	3033	0.044	0.945	17.040	16:1 2OH	1.21	ECL deviates -0.008		
12.994	2044	0.048	0.945	17.408	17:0 10-methyl	0.82	ECL deviates -0.001		
13.147	995	0.038	----	17.495		----			
13.547	8481	0.044	0.946	17.722	Sum In Feature 5	3.40	ECL deviates  0.002	18:2 w6,9c/18:0 ante	
13.632	16199	0.049	0.946	17.770	18:1 w9c	6.49	ECL deviates  0.001		
13.726	22001	0.047	0.946	17.824	Sum In Feature 8	8.81	ECL deviates  0.001	18:1 w7c	
13.875	2439	0.047	0.946	17.908	18:1 w5c	0.98	ECL deviates -0.011		
14.035	6900	0.047	0.946	17.999	18:0	2.76	ECL deviates -0.001	Reference -0.004	
14.177	1917	0.039	0.946	18.080	18:1 w7c 11-methyl	0.77	ECL deviates -0.001		
14.610	7405	0.066	----	18.328		----			
14.727	7170	0.053	0.947	18.394	18:0 10-methyl, TBSA	2.88	ECL deviates  0.002		
14.784	3755	0.045	----	18.427		----			
15.344	1172	0.044	0.948	18.747	Sum In Feature 6	0.47	ECL deviates -0.009	19:1 w11c/19:1 w9c	
15.620	18821	0.053	0.948	18.905	19:0 cyclo w8c	7.56	ECL deviates  0.003		
15.881	350566	0.153	----	19.054		----	> max ar/ht		
16.475	1101	0.038	0.949	19.398	20:4 w6,9,12,15c	0.44	ECL deviates  0.003		
16.631	1097	0.049	----	19.488		----			
16.893	2784	0.104	0.949	19.640	20:0 iso	----	> max ar/ht		
17.119	1202	0.049	0.949	19.771	20:1 w9c	0.48	ECL deviates  0.001		
17.516	875	0.041	0.950	20.000	20:0	0.35	ECL deviates  0.000	Reference -0.004	
17.849	871	0.036	----	20.193		----	> max rt		
----	24619	---	----	----	Summed Feature 3	9.89	16:1 w7c/16:1 w6c	16:1 w6c/16:1 w7c	
----	8481	---	----	----	Summed Feature 5	3.40	18:2 w6,9c/18:0 ante	18:0 ante/18:2 w6,9c	
----	1172	---	----	----	Summed Feature 6	0.47	19:1 w11c/19:1 w9c	19:1 w9c/19:1 w11c	
----	22001	---	----	----	Summed Feature 8	8.81	18:1 w7c	18:1 w6c	
----	32121	---	----	----	Summed Feature 9	12.87	17:1 iso w9c	16:0 10-methyl	

ECL Deviation: 0.004                            Reference ECL Shift: 0.003      Number Reference Peaks: 12
Total Response: 663307                         Total Named: 248906
Percent Named: 37.52%                         Total Amount: 240438
Profile Comment:   Percent named is less than 85.00.

*** Library match not attempted
