# Supplementary material for: A green garlic (Allium sativum L.) based intercropping system reduces the strain of continuous monocropping in cucumber (Cucumis sativus L.) by adjusting the micro-ecological environment of soil
Source: PeerJ. 2019 Jul 15;7:e7267. doi: 10.7717/peerj.7267 (PMC6637937; doi:10.7717/peerj.7267)
Supplement: Data S1 [file peerj-07-7267-s001.zip › supplemental_Data_S1/45 days after interplanted/GB-3.rtf]

Volume: DATA            File: E131084.29A        Samp Ctr: 12                ID Number: 1009 
Type: Samp                   Bottle: 7                        Method: TSBA6 
Created: 1/8/2013 2:43:20 PM 
Sample ID: 44 


RT	Response	Ar/Ht	RFact	ECL	Peak Name	Percent	Comment1	Comment2	
1.646	4.543E+8	0.029	----	7.015	SOLVENT PEAK	----	< min rt		
1.778	3823	0.025	----	7.274		----	< min rt		
2.286	479	0.023	----	8.272		----	< min rt		
3.060	582	0.021	----	9.789		----			
4.405	603	0.036	----	11.579		----			
4.908	2275	0.034	1.021	12.099	11:0 iso 3OH	0.65	ECL deviates  0.010		
5.113	2441	0.037	----	12.276		----			
5.309	338	0.039	1.008	12.445	11:0 3OH	0.10	ECL deviates  0.007		
5.502	401	0.030	1.003	12.612	13:0 iso	0.11	ECL deviates -0.002	Reference -0.007	
6.402	483	0.042	----	13.327		----			
6.806	1893	0.035	0.976	13.621	14:0 iso	0.51	ECL deviates  0.002	Reference -0.002	
7.327	2394	0.037	0.969	14.000	14:0	0.65	ECL deviates  0.000	Reference -0.004	
7.781	7505	0.053	----	14.293		----			
8.010	1350	0.046	0.962	14.442	15:1 iso G	0.36	ECL deviates  0.002		
8.294	20669	0.038	0.959	14.625	15:0 iso	5.52	ECL deviates  0.002	Reference -0.002	
8.433	12478	0.039	0.958	14.715	15:0 anteiso	3.33	ECL deviates  0.002	Reference -0.001	
8.638	451	0.037	0.956	14.847	15:1 w6c	0.12	ECL deviates -0.009		
8.873	2214	0.038	0.955	14.999	15:0	----	ECL deviates -0.001		
8.964	929	0.031	----	15.054		----			
9.618	2733	0.062	0.951	15.445	16:1 iso G	0.72	ECL deviates  0.003		
9.921	11636	0.041	0.949	15.627	16:0 iso	3.08	ECL deviates  0.000	Reference -0.003	
10.160	3319	0.052	0.949	15.770	16:1 w9c	0.88	ECL deviates -0.004		
10.240	37062	0.042	0.948	15.818	Sum In Feature 3	9.79	ECL deviates -0.004	16:1 w7c/16:1 w6c	
10.390	8541	0.044	0.948	15.908	16:1 w5c	2.25	ECL deviates -0.001		
10.543	48199	0.042	0.947	15.999	16:0	12.72	ECL deviates -0.001	Reference -0.003	
10.625	642	0.031	----	16.047		----			
11.083	103529	0.056	----	16.311		----			
11.289	52101	0.078	0.946	16.429	Sum In Feature 9	13.73	ECL deviates -0.003	16:0 10-methyl	
11.454	12818	0.090	0.946	16.525	17:1 anteiso w9c	----	> max ar/ht		
11.636	13746	0.054	0.946	16.629	17:0 iso	3.62	ECL deviates -0.001	Reference -0.002	
11.797	12567	0.052	0.945	16.722	17:0 anteiso	3.31	ECL deviates -0.001	Reference -0.002	
11.919	5429	0.055	0.945	16.793	17:1 w8c	1.43	ECL deviates  0.001		
12.085	11589	0.053	0.945	16.888	17:0 cyclo	3.05	ECL deviates  0.000		
12.277	3400	0.056	0.945	16.999	17:0	0.89	ECL deviates -0.001	Reference -0.002	
12.346	4931	0.043	0.945	17.038	16:1 2OH	1.30	ECL deviates -0.010		
12.470	448	0.037	----	17.109		----			
12.994	3126	0.052	0.945	17.406	17:0 10-methyl	0.82	ECL deviates -0.003		
13.149	2096	0.057	----	17.494		----			
13.548	10850	0.046	0.946	17.720	Sum In Feature 5	2.86	ECL deviates  0.000	18:2 w6,9c/18:0 ante	
13.632	22291	0.048	0.946	17.768	18:1 w9c	5.87	ECL deviates -0.001		
13.727	28859	0.048	0.946	17.821	Sum In Feature 8	7.60	ECL deviates -0.002	18:1 w7c	
13.874	5236	0.059	----	17.905		----			
14.037	10326	0.045	0.946	17.997	18:0	2.72	ECL deviates -0.003	Reference -0.003	
14.177	2837	0.046	0.946	18.077	18:1 w7c 11-methyl	0.75	ECL deviates -0.004		
14.604	29431	0.064	----	18.321		----			
14.726	15727	0.059	0.947	18.390	18:0 10-methyl, TBSA	4.15	ECL deviates -0.002		
14.787	8044	0.053	----	18.425		----			
15.012	640	0.045	----	18.554		----			
15.345	2128	0.047	----	18.743		----			
15.621	20081	0.052	0.948	18.901	19:0 cyclo w8c	5.30	ECL deviates -0.001		
15.876	271740	0.154	----	19.047		----	> max ar/ht		
16.476	1521	0.045	0.949	19.393	20:4 w6,9,12,15c	0.40	ECL deviates -0.002		
16.908	1262	0.067	0.949	19.643	20:0 iso	0.33	ECL deviates  0.008	Reference  0.009	
17.120	2487	0.060	0.949	19.765	20:1 w9c	0.66	ECL deviates -0.005		
17.518	1630	0.042	0.950	19.995	20:0	0.43	ECL deviates -0.005	Reference -0.003	
17.851	1490	0.046	----	20.187		----	> max rt		
18.181	1473	0.078	----	20.378		----	> max rt		
----	37062	---	----	----	Summed Feature 3	9.79	16:1 w7c/16:1 w6c	16:1 w6c/16:1 w7c	
----	10850	---	----	----	Summed Feature 5	2.86	18:2 w6,9c/18:0 ante	18:0 ante/18:2 w6,9c	
----	28859	---	----	----	Summed Feature 8	7.60	18:1 w7c	18:1 w6c	
----	52101	---	----	----	Summed Feature 9	13.73	17:1 iso w9c	16:0 10-methyl	

ECL Deviation: 0.004                            Reference ECL Shift: 0.004      Number Reference Peaks: 13
Total Response: 827794                         Total Named: 378499
Percent Named: 45.72%                         Total Amount: 373313
Profile Comment:   Percent named is less than 85.00.

*** No Matches found in TSBA6
