# Supplementary material for: A green garlic (Allium sativum L.) based intercropping system reduces the strain of continuous monocropping in cucumber (Cucumis sativus L.) by adjusting the micro-ecological environment of soil
Source: PeerJ. 2019 Jul 15;7:e7267. doi: 10.7717/peerj.7267 (PMC6637937; doi:10.7717/peerj.7267)
Supplement: Data S1 [file peerj-07-7267-s001.zip › supplemental_Data_S1/45 days after interplanted/GR-1.rtf]

Volume: DATA            File: E131084.29A        Samp Ctr: 17                ID Number: 1002 
Type: Samp                   Bottle: 3                        Method: TSBA6 
Created: 1/8/2013 4:44:52 PM 
Sample ID: 51 


RT	Response	Ar/Ht	RFact	ECL	Peak Name	Percent	Comment1	Comment2	
1.646	4.543E+8	0.028	----	7.004	SOLVENT PEAK	----	< min rt		
1.778	2008	0.014	----	7.264		----	< min rt		
2.285	298	0.025	----	8.259		----	< min rt		
2.793	292	0.026	----	9.257		----			
3.059	314	0.022	----	9.780		----			
4.411	563	0.039	----	11.588		----			
4.548	242	0.028	----	11.735		----			
4.909	1677	0.030	1.021	12.101	11:0 iso 3OH	0.47	ECL deviates  0.012		
5.502	363	0.027	1.002	12.612	13:0 iso	0.10	ECL deviates -0.002	Reference -0.007	
6.411	361	0.031	----	13.333		----			
6.807	1728	0.037	0.975	13.621	14:0 iso	0.46	ECL deviates  0.002	Reference -0.002	
7.329	2114	0.036	0.967	14.000	14:0	0.56	ECL deviates  0.000	Reference -0.003	
7.779	4136	0.054	----	14.291		----			
8.009	1258	0.037	0.960	14.440	15:1 iso G	0.33	ECL deviates  0.000		
8.294	16462	0.037	0.958	14.624	15:0 iso	4.35	ECL deviates  0.001	Reference -0.001	
8.434	10147	0.038	0.957	14.715	15:0 anteiso	2.68	ECL deviates  0.002	Reference  0.000	
8.877	1778	0.038	0.953	15.001	15:0	----	ECL deviates  0.001		
8.965	691	0.037	----	15.054		----			
9.377	658	0.041	----	15.301		----			
9.621	2240	0.057	0.949	15.446	16:1 iso G	0.59	ECL deviates  0.004		
9.922	8885	0.040	0.948	15.626	16:0 iso	2.32	ECL deviates -0.001	Reference -0.002	
10.157	2932	0.044	0.947	15.767	16:1 w9c	0.77	ECL deviates -0.007		
10.240	36589	0.042	0.947	15.817	Sum In Feature 3	9.56	ECL deviates -0.005	16:1 w7c/16:1 w6c	
10.391	7735	0.042	0.947	15.908	16:1 w5c	2.02	ECL deviates -0.001		
10.545	41378	0.042	0.946	15.999	16:0	10.80	ECL deviates -0.001	Reference -0.002	
10.628	472	0.026	----	16.047		----			
11.099	219890	0.055	----	16.320		----			
11.292	81644	0.113	0.945	16.431	Sum In Feature 9	21.27	ECL deviates -0.001	16:0 10-methyl	
11.637	18795	0.070	0.944	16.630	17:0 iso	4.89	ECL deviates  0.000	Reference -0.002	
11.797	14400	0.061	0.944	16.723	17:0 anteiso	3.75	ECL deviates  0.000	Reference -0.002	
11.921	7388	0.066	0.944	16.794	17:1 w8c	1.92	ECL deviates  0.002		
12.086	14255	0.068	0.944	16.890	17:0 cyclo	3.71	ECL deviates  0.002		
12.276	2557	0.044	0.944	17.000	17:0	0.67	ECL deviates  0.000	Reference -0.003	
12.347	4752	0.052	0.944	17.040	16:1 2OH	1.24	ECL deviates -0.008		
12.997	2113	0.041	0.944	17.409	17:0 10-methyl	0.55	ECL deviates  0.000		
13.151	1350	0.046	----	17.496		----			
13.547	9149	0.044	0.945	17.721	Sum In Feature 5	2.38	ECL deviates  0.001	18:2 w6,9c/18:0 ante	
13.632	20402	0.048	0.945	17.769	18:1 w9c	5.31	ECL deviates  0.000		
13.726	31150	0.050	0.945	17.823	Sum In Feature 8	8.11	ECL deviates  0.000	18:1 w7c	
13.882	3107	0.057	0.945	17.912	18:1 w5c	0.81	ECL deviates -0.007		
14.034	8700	0.046	0.945	17.998	18:0	2.27	ECL deviates -0.002	Reference -0.006	
14.180	3212	0.054	0.945	18.081	18:1 w7c 11-methyl	0.84	ECL deviates  0.000		
14.606	36497	0.061	----	18.325		----			
14.726	23612	0.091	0.946	18.394	18:0 10-methyl, TBSA	----	> max ar/ht		
15.016	545	0.035	----	18.560		----			
15.343	1271	0.043	0.946	18.747	Sum In Feature 6	0.33	ECL deviates -0.009	19:1 w11c/19:1 w9c	
15.620	20949	0.048	0.947	18.906	19:0 cyclo w8c	5.47	ECL deviates  0.004		
15.887	283691	0.156	----	19.060		----	> max ar/ht		
16.478	1733	0.034	0.947	19.401	20:4 w6,9,12,15c	0.45	ECL deviates  0.006		
16.604	399	0.035	----	19.474		----			
16.773	196	0.031	----	19.572		----			
17.123	2063	0.052	0.948	19.775	20:1 w9c	0.54	ECL deviates  0.005		
17.231	585	0.047	0.948	19.837	20:1 w7c	0.15	ECL deviates  0.006		
17.514	1224	0.045	0.948	20.001	20:0	0.32	ECL deviates  0.001	Reference -0.006	
17.858	1115	0.045	----	20.200		----	> max rt		
18.187	1315	0.068	----	20.391		----	> max rt		
18.485	592	0.035	----	20.563		----	> max rt		
----	36589	---	----	----	Summed Feature 3	9.56	16:1 w7c/16:1 w6c	16:1 w6c/16:1 w7c	
----	9149	---	----	----	Summed Feature 5	2.38	18:2 w6,9c/18:0 ante	18:0 ante/18:2 w6,9c	
----	1271	---	----	----	Summed Feature 6	0.33	19:1 w11c/19:1 w9c	19:1 w9c/19:1 w11c	
----	31150	---	----	----	Summed Feature 8	8.11	18:1 w7c	18:1 w6c	
----	81644	---	----	----	Summed Feature 9	21.27	17:1 iso w9c	16:0 10-methyl	

ECL Deviation: 0.004                            Reference ECL Shift: 0.004      Number Reference Peaks: 12
Total Response: 956867                         Total Named: 382957
Percent Named: 40.02%                         Total Amount: 386666
Profile Comment:   Percent named is less than 85.00.

*** Library match not attempted
