# Supplementary material for: A green garlic (Allium sativum L.) based intercropping system reduces the strain of continuous monocropping in cucumber (Cucumis sativus L.) by adjusting the micro-ecological environment of soil
Source: PeerJ. 2019 Jul 15;7:e7267. doi: 10.7717/peerj.7267 (PMC6637937; doi:10.7717/peerj.7267)
Supplement: Data S1 [file peerj-07-7267-s001.zip › supplemental_Data_S1/45 days after interplanted/GR-2.rtf]

Volume: DATA            File: E131084.29A        Samp Ctr: 18                ID Number: 1003 
Type: Samp                   Bottle: 4                        Method: TSBA6 
Created: 1/8/2013 5:08:59 PM 
Sample ID: 52 


RT	Response	Ar/Ht	RFact	ECL	Peak Name	Percent	Comment1	Comment2	
1.646	4.567E+8	0.028	----	7.006	SOLVENT PEAK	----	< min rt		
1.778	5419	0.023	----	7.265		----	< min rt		
2.291	213	0.022	----	8.273		----	< min rt		
2.791	323	0.031	----	9.255		----			
3.058	461	0.023	----	9.779		----			
4.404	418	0.035	----	11.575		----			
4.908	2341	0.032	1.021	12.095	11:0 iso 3OH	0.76	ECL deviates  0.006		
5.507	610	0.039	1.002	12.613	13:0 iso	0.19	ECL deviates -0.001	Reference -0.003	
5.800	163	0.020	----	12.866		----			
6.807	1630	0.034	0.975	13.620	14:0 iso	0.50	ECL deviates  0.001	Reference -0.001	
7.331	2155	0.038	0.967	14.000	14:0	0.66	ECL deviates  0.000	Reference -0.002	
7.780	4160	0.051	----	14.291		----			
8.011	1011	0.043	0.960	14.441	15:1 iso G	0.31	ECL deviates  0.001		
8.294	16226	0.037	0.958	14.624	15:0 iso	4.92	ECL deviates  0.001	Reference -0.001	
8.434	9936	0.039	0.957	14.714	15:0 anteiso	3.01	ECL deviates  0.001	Reference -0.001	
8.877	1907	0.040	0.953	15.001	15:0	----	ECL deviates  0.001		
8.967	865	0.036	----	15.055		----			
9.371	680	0.037	----	15.297		----			
9.625	3108	0.069	0.949	15.449	16:1 iso G	0.93	ECL deviates  0.007		
9.923	8829	0.040	0.948	15.627	16:0 iso	2.65	ECL deviates  0.000	Reference -0.002	
10.160	2976	0.058	0.947	15.769	16:1 w9c	0.89	ECL deviates -0.005		
10.241	37056	0.042	0.947	15.817	Sum In Feature 3	11.10	ECL deviates -0.005	16:1 w7c/16:1 w6c	
10.393	7782	0.043	0.947	15.908	16:1 w5c	2.33	ECL deviates -0.001		
10.544	41832	0.042	0.946	15.999	16:0	12.52	ECL deviates -0.001	Reference -0.003	
11.103	241230	0.056	----	16.322		----			
11.292	62342	0.089	0.945	16.431	Sum In Feature 9	18.63	ECL deviates -0.001	16:0 10-methyl	
11.637	6327	0.042	0.944	16.630	17:0 iso	1.89	ECL deviates  0.000	Reference -0.002	
11.797	6518	0.045	0.944	16.723	17:0 anteiso	1.95	ECL deviates  0.000	Reference -0.002	
11.920	2128	0.044	0.944	16.794	17:1 w8c	0.64	ECL deviates  0.002		
12.086	7146	0.047	0.944	16.889	17:0 cyclo	2.13	ECL deviates  0.001		
12.278	1247	0.036	0.944	17.000	17:0	0.37	ECL deviates  0.000	Reference -0.002	
12.345	3159	0.042	0.944	17.038	16:1 2OH	0.94	ECL deviates -0.010		
12.465	423	0.043	----	17.106		----			
12.993	2271	0.050	0.944	17.406	17:0 10-methyl	0.68	ECL deviates -0.003		
13.153	1571	0.053	----	17.497		----			
13.548	8417	0.047	0.945	17.721	Sum In Feature 5	2.52	ECL deviates  0.001	18:2 w6,9c/18:0 ante	
13.631	20276	0.047	0.945	17.768	18:1 w9c	6.06	ECL deviates -0.001		
13.727	31819	0.048	0.945	17.822	Sum In Feature 8	9.51	ECL deviates -0.001	18:1 w7c	
13.882	3003	0.050	0.945	17.910	18:1 w5c	0.90	ECL deviates -0.009		
14.036	8728	0.047	0.945	17.998	18:0	2.61	ECL deviates -0.002	Reference -0.004	
14.178	2990	0.044	0.945	18.079	18:1 w7c 11-methyl	0.89	ECL deviates -0.002		
14.605	44033	0.063	----	18.323		----			
14.722	23524	0.083	0.946	18.390	18:0 10-methyl, TBSA	----	> max ar/ht		
15.347	1474	0.053	0.946	18.747	Sum In Feature 6	0.44	ECL deviates -0.009	19:1 w11c/19:1 w9c	
15.619	22506	0.051	0.947	18.902	19:0 cyclo w8c	6.74	ECL deviates  0.000		
15.887	286474	0.153	----	19.056		----	> max ar/ht		
16.481	1621	0.045	0.947	19.399	20:4 w6,9,12,15c	0.49	ECL deviates  0.004		
16.768	663	0.042	----	19.564		----			
16.898	2077	0.078	0.948	19.640	20:0 iso	0.62	ECL deviates  0.005	Reference  0.003	
17.116	2249	0.054	0.948	19.766	20:1 w9c	0.67	ECL deviates -0.004		
17.230	470	0.037	0.948	19.831	20:1 w7c	0.14	ECL deviates  0.000		
17.517	1367	0.044	0.948	19.997	20:0	0.41	ECL deviates -0.003	Reference -0.005	
17.849	1262	0.044	----	20.189		----	> max rt		
18.487	776	0.042	----	20.557		----	> max rt		
----	37056	---	----	----	Summed Feature 3	11.10	16:1 w7c/16:1 w6c	16:1 w6c/16:1 w7c	
----	8417	---	----	----	Summed Feature 5	2.52	18:2 w6,9c/18:0 ante	18:0 ante/18:2 w6,9c	
----	1474	---	----	----	Summed Feature 6	0.44	19:1 w11c/19:1 w9c	19:1 w9c/19:1 w11c	
----	31819	---	----	----	Summed Feature 8	9.51	18:1 w7c	18:1 w6c	
----	62342	---	----	----	Summed Feature 9	18.63	17:1 iso w9c	16:0 10-methyl	

ECL Deviation: 0.004                            Reference ECL Shift: 0.003      Number Reference Peaks: 13
Total Response: 938615                         Total Named: 333627
Percent Named: 35.54%                         Total Amount: 340185
Profile Comment:   Percent named is less than 85.00.

*** Library match not attempted
