# Supplementary material for: A green garlic (Allium sativum L.) based intercropping system reduces the strain of continuous monocropping in cucumber (Cucumis sativus L.) by adjusting the micro-ecological environment of soil
Source: PeerJ. 2019 Jul 15;7:e7267. doi: 10.7717/peerj.7267 (PMC6637937; doi:10.7717/peerj.7267)
Supplement: Data S1 [file peerj-07-7267-s001.zip › supplemental_Data_S1/45 days after interplanted/GR-3.rtf]

Volume: DATA            File: E131084.29A        Samp Ctr: 19                ID Number: 1004 
Type: Samp                   Bottle: 5                        Method: TSBA6 
Created: 1/8/2013 5:33:18 PM 
Sample ID: 53 


RT	Response	Ar/Ht	RFact	ECL	Peak Name	Percent	Comment1	Comment2	
1.646	4.57E+8	0.029	----	7.005	SOLVENT PEAK	----	< min rt		
1.778	-----	---	----	7.264		----	< min rt		
3.359	363	0.034	----	10.269		----			
4.908	1355	0.030	1.021	12.098	11:0 iso 3OH	0.45	ECL deviates  0.009		
6.806	1337	0.035	0.975	13.620	14:0 iso	0.43	ECL deviates  0.001	Reference -0.002	
7.330	1827	0.038	0.967	14.000	14:0	0.58	ECL deviates  0.000	Reference -0.002	
7.792	2726	0.050	----	14.299		----			
8.013	1024	0.035	0.960	14.441	15:1 iso G	0.32	ECL deviates  0.001		
8.295	15071	0.038	0.958	14.624	15:0 iso	4.71	ECL deviates  0.001	Reference -0.001	
8.434	9146	0.038	0.957	14.714	15:0 anteiso	2.85	ECL deviates  0.001	Reference  0.000	
8.644	519	0.039	0.955	14.850	15:1 w6c	0.16	ECL deviates -0.006		
8.877	1706	0.039	0.953	15.000	15:0	----	ECL deviates  0.000		
8.965	608	0.031	----	15.053		----			
9.384	476	0.035	----	15.304		----			
9.625	2230	0.057	0.949	15.448	16:1 iso G	0.69	ECL deviates  0.006		
9.922	8549	0.040	0.948	15.626	16:0 iso	2.64	ECL deviates -0.001	Reference -0.002	
10.159	2981	0.050	0.947	15.768	16:1 w9c	0.92	ECL deviates -0.006		
10.240	34231	0.045	0.947	15.816	Sum In Feature 3	10.57	ECL deviates -0.006	16:1 w7c/16:1 w6c	
10.392	7184	0.042	0.947	15.907	16:1 w5c	2.22	ECL deviates -0.002		
10.543	40600	0.042	0.946	15.998	16:0	12.53	ECL deviates -0.002	Reference -0.003	
11.082	96903	0.060	----	16.309		----			
11.289	45988	0.077	0.945	16.428	Sum In Feature 9	14.17	ECL deviates -0.004	16:0 10-methyl	
11.447	8661	0.075	0.945	16.519	17:1 anteiso w9c	2.67	ECL deviates -0.005		
11.545	2418	0.041	0.945	16.576	unknown 16.582	----	ECL deviates -0.006		
11.636	11218	0.055	0.944	16.628	17:0 iso	3.45	ECL deviates -0.002	Reference -0.003	
11.799	8742	0.051	0.944	16.722	17:0 anteiso	2.69	ECL deviates -0.001	Reference -0.001	
11.919	3873	0.056	0.944	16.792	17:1 w8c	1.19	ECL deviates  0.000		
12.085	8155	0.049	0.944	16.888	17:0 cyclo	2.51	ECL deviates  0.000		
12.278	1220	0.039	0.944	16.999	17:0	0.38	ECL deviates -0.001	Reference -0.002	
12.345	2927	0.041	----	17.037		----			
12.993	2585	0.053	0.944	17.405	17:0 10-methyl	0.80	ECL deviates -0.004		
13.147	1042	0.037	----	17.492		----			
13.547	8295	0.042	0.945	17.719	Sum In Feature 5	2.56	ECL deviates -0.001	18:2 w6,9c/18:0 ante	
13.634	20005	0.048	0.945	17.768	18:1 w9c	6.16	ECL deviates -0.001		
13.727	30779	0.047	0.945	17.821	Sum In Feature 8	9.48	ECL deviates -0.002	18:1 w7c	
13.875	3299	0.059	----	17.905		----			
14.037	8725	0.047	0.945	17.997	18:0	2.69	ECL deviates -0.003	Reference -0.004	
14.179	2894	0.044	0.945	18.078	18:1 w7c 11-methyl	0.89	ECL deviates -0.003		
14.604	11148	0.066	----	18.322		----			
14.727	8974	0.058	0.946	18.392	18:0 10-methyl, TBSA	2.77	ECL deviates  0.000		
14.785	4237	0.049	----	18.425		----			
15.025	766	0.048	----	18.563		----			
15.339	1306	0.045	----	18.742		----		Reference  0.009	
15.619	22918	0.052	0.947	18.902	19:0 cyclo w8c	7.08	ECL deviates  0.000		
15.864	276308	0.146	----	19.043		----	> max ar/ht		
16.477	2052	0.050	0.947	19.397	20:4 w6,9,12,15c	0.63	ECL deviates  0.002		
16.614	506	0.041	----	19.476		----			
17.121	1527	0.040	0.948	19.769	20:1 w9c	0.47	ECL deviates -0.001		
17.511	1123	0.046	0.948	19.995	20:0	0.35	ECL deviates -0.005	Reference -0.008	
17.849	1167	0.046	----	20.190		----	> max rt		
18.188	916	0.056	----	20.386		----	> max rt		
18.482	450	0.030	----	20.556		----	> max rt		
----	34231	---	----	----	Summed Feature 3	10.57	16:1 w7c/16:1 w6c	16:1 w6c/16:1 w7c	
----	8295	---	----	----	Summed Feature 5	2.56	18:2 w6,9c/18:0 ante	18:0 ante/18:2 w6,9c	
----	30779	---	----	----	Summed Feature 8	9.48	18:1 w7c	18:1 w6c	
----	45988	---	----	----	Summed Feature 9	14.17	17:1 iso w9c	16:0 10-methyl	

ECL Deviation: 0.003                            Reference ECL Shift: 0.004      Number Reference Peaks: 12
Total Response: 726403                         Total Named: 323788
Percent Named: 44.57%                         Total Amount: 310568
Profile Comment:   Percent named is less than 85.00.

*** No Matches found in TSBA6
